# Algorithms for reconstruction of chromosomal structures

**DOI:** 10.1186/s12859-016-0878-z

**Published:** 2016-01-19

**Authors:** Vassily Lyubetsky, Roman Gershgorin, Alexander Seliverstov, Konstantin Gorbunov

**Affiliations:** Institute for Information Transmission Problems of the Russian Academy of Sciences (Kharkevich Institute), Bolshoi Karetnyi lane, 19, 127051 Moscow, Russia

**Keywords:** Chromosomal structure, Rearrangement of chromosomal structure, Ancestral chromosomal structure, Distance between chromosomal structures, Lowest weight transformation of one chromosome structure into another, Exact linear algorithm calculating the distance and transformation, Generation of a phylogenetic tree of chromosomal structures, Exact reconstruction algorithm with cubic complexity

## Abstract

**Background:**

One of the main aims of phylogenomics is the reconstruction of objects defined in the leaves along the whole phylogenetic tree to minimize the specified functional, which may also include the phylogenetic tree generation. Such objects can include nucleotide and amino acid sequences, chromosomal structures, etc. The structures can have any set of linear and circular chromosomes, variable gene composition and include any number of paralogs, as well as any weights of individual evolutionary operations to transform a chromosome structure. Many heuristic algorithms were proposed for this purpose, but there are just a few exact algorithms with low (linear, cubic or similar) polynomial computational complexity among them to our knowledge. The algorithms naturally start from the calculation of both the distance between two structures and the shortest sequence of operations transforming one structure into another. Such calculation per se is an NP-hard problem.

**Results:**

A general model of chromosomal structure rearrangements is considered. Exact algorithms with almost linear or cubic polynomial complexities have been developed to solve the problems for the case of any chromosomal structure but with certain limitations on operation weights. The computer programs are tested on biological data for the problem of mitochondrial or plastid chromosomal structure reconstruction. To our knowledge, no computer programs are available for this model.

**Conclusions:**

Exactness of the proposed algorithms and such low polynomial complexities were proved. The reconstructed evolutionary trees of mitochondrial and plastid chromosomal structures as well as the ancestral states of the structures appear to be reasonable.

**Electronic supplementary material:**

The online version of this article (doi:10.1186/s12859-016-0878-z) contains supplementary material, which is available to authorized users.

## Background

### Calculation of the distance and shortest sequence between chromosome structures

Reconstruction of chromosome structures is considered based on the model of chromosome structure as an arbitrary set of paths and circles composed of vectors: genes denoted by the index *i* and paralogs of any gene *i* denoted by the index *i.j*. The model includes four operations transforming one structure into another referred to as *standard* as well as *accessory* operations deleting and inserting a chromosome region. Detailed description of the model is given in the Section “Definition of the model of chromosome structure”. Significant constraints were imposed on the model in [1] and many other publications: constant gene content is provided in the sequence of transformations from one structure into another, paralogs are not allowed, operation weights are equal (thus, are not used), etc.

If the weights are considered at the level of an exact algorithm, they should have a specific form considering the NP-hardness of the considered problem. Part “Exact linear algorithm calculating the distance between chromosome structures” of this work proposes an almost linear algorithm transforming one chromosome structure into another working in the absence of all these constraints, although we had to impose a condition on the proportion between operation weights. This cannot be avoided owing to the NP-hardness of the considered problem in general terms. The proposed proportion of weights differs from commonly accepted ones. The reconstruction of structures in Part “Reconstruction of chromosome structures for mitochondria of sporozoans and plastids of rhodophytic branch” had to rely on the proportion between the weights for which our algorithm is heuristic although close to exact.

The term *almost* appears due to the problem of paralogs, it is solved by means of linear programming, which is known to produce an exact solution within almost linear time [2–4].

Although the algorithm proposed here conforms to the basic concept of the algorithm in [1], it is radically more complex; while the exactness proof was essentially trivial for the algorithm in [1], the exactness here is a kind of a theory. The current work introduces a sequence of lemmas constituting this proof, although certain details were given in [5, 6]. The algorithm was implemented as a computer program available together with calculation samples, tests on artificial data, and the user manual at http://lab6.iitp.ru/en/chromoggl/.

Computer-aided comparison of our algorithm with other heuristic ones is problematic, since the model of chromosome rearrangement at the level of generalization used here was considered only in [7–10] to our knowledge. These publications offer no program implementation. It should be noted that a computer implementation in the absence of the insertion and deletion operations as well as the operation weights was presented in [11]; however, this is far from our case.

Let us recall that an algorithm is *exact* if it was proved that it always produces the minimum corresponding functional; in this case, the minimum total weight for the sequence of operations transforming one chromosome structure into another. Here we use a slightly relaxed version of this: our algorithm produces the sequence with the total weight differing from the minimum by a fixed *additive* quantity *d,* for example 0.7 (see the Section “Condition for the exactness of the algorithm and operation weight values”). One more concern pertinent to the algorithm exactness is discussed in the beginning of Part “Algorithm for the reconstruction of chromosome structures with cubic complexity and sufficient approximation ratio” the solution produced by the algorithm can differ from the minimum by a *multiplicative* value *k*, for example 2. Apparently, an algorithm with such complexity can distort the tree topology (see Part “Reconstruction of chromosome structures for mitochondria of sporozoans and plastids of rhodophytic branch”). A *linear* algorithm has linear computation time over the input size and uses linear space.

### Reconstruction of chromosome structures

As Part “Exact linear algorithm calculating the distance between chromosome structures” shows, a matrix of pairwise distances can be easily generated for a given set of chromosome structures. Phylogenetic tree with the best conformity with this matrix is required. This means that the distance between any two leaves along the tree is the closest to the corresponding distance in the matrix; the residual is determined for each pair of leaves as the difference between these distances. Since this problem is NP-hard, a tree with an a priori unknown conformity with the matrix is generated.

Many popular algorithms for tree reconstruction require more informative input such as an alignment of nucleotide or amino acid sequences. Widely used sophisticated reconstruction programs (PhyloBayes, MrBayes, RAxML, PHYML, etc.) use a linear form of the reconstructed object and are, apparently, inapplicable in our case. For chromosome structures, a simple algorithm based on the conformity with the distance matrix rapidly generates their evolutionary tree. The data and results described in Part “Reconstruction of chromosome structures for mitochondria of sporozoans and plastids of rhodophytic branch” demonstrate that this algorithm outputs sensible trees for mitochondria and plastids. The algorithm is given at the same page http://lab6.iitp.ru/en/chromoggl/. Basically, it is a UPGMA variant, but neighbor joining or other algorithm proficient in bringing into conformity with the distance matrix can be used instead.

Part “Algorithm for the reconstruction of chromosome structures with cubic complexity and sufficient approximation ratio” considers the natural problem of reconstructing chromosome structures in internal nodes of a phylogenetic tree from the chromosome structures specified in its leaves using the tree generated by the algorithm or an existing one.

Backing away the problem of chromosome structures, for species the algorithms reconstructing evolutionary scenarios with cubic computational complexity are given in [12]. A similar problem for regulatory systems is considered, e.g., in [13, 14]. Actually, to our knowledge, many heuristic algorithms but few exact algorithms with low (linear, cubic, etc.) polynomial complexity were proposed for the reconstruction of objects specified in the leaves. This specifically applies to the problem of chromosome structure reconstruction using the model defined in the Section “Definition of the Model of Chromosome Structure”.

*Arrangement* is a function that assigns a chromosome structure to each node of the tree; hereafter, a node and an object assigned to it are *synonymous*. The functional defined for all arrangements (for a given tree) or also for all trees with specified leaves amounts to the total distance between edge ends for all edges. In this case, the breakpoint and biological *distances* are considered; the latter essentially is also the edit distance between two structures, and thus is an analog of pairwise sequence alignment.

We recall that the *breakpoint distance* is the number of gene extremity pairs that are *adjacent* (or “*glued together*”) in one structure and not neighboring or missing in the other plus the number of genes that are present in one structure and absent in the other. The *biological distance* between structures is the minimum total weight for the sequence of operations transforming one structure into the other; all operations are a priori given individual weights. The calculation of the distances is also discussed in the Sections “Calculation of the breakpoint and biological distances for structures with paralogs” and “Calculation of biological distance with paths present”.

Let us recall that the considered structures can have *any number* of linear and circular chromosomes, *variable* gene content, and *paralogs*; *all operations*, both standard and accessory, as well as *any operation weights* are allowed.

It has been proven that the result of the algorithm described in Part “Algorithm for the reconstruction of chromosome structures with cubic complexity and sufficient approximation ratio” falls within the [*a*, *ka*] segment, where *a* is the absolute minimum and *k* is a small *approximation ratio*. Algorithms with such a property are called *approximate algorithms.* Part “Exact linear algorithm calculating the distance between chromosome structures” and [1, 5–7, 12] sets the coefficient *k* equal to 1, while in Part “Algorithm for the reconstruction of chromosome structures with cubic complexity and sufficient approximation ratio” it equals 2 or 11/6. These cases are fundamentally different from heuristic algorithms, whose results are in unknown relation to the minimum (“true”) solution. Moreover, even the convergence of the computational process has not been confirmed for many heuristic algorithms, and it is terminated following a not substantiated rule. In practical terms, the approximation ratio of 2 can give rise to inadequate trees. This difficulty is concerned for our data in Part “Reconstruction of chromosome structures for mitochondria of sporozoans and plastids of rhodophytic branch”.

Part “Algorithm for the reconstruction of chromosome structures with cubic complexity and sufficient approximation ratio” follows the work in [15] presenting an algorithm of reconstruction for 0–1 sequences. It is readily applicable to biological sequences. To our knowledge, reconstruction of chromosome structures within the frames of the model described in the Section “Definition of the Model of Chromosome Structure” has never been considered as an exact problem or realized as a computer program.

Thus, the problem in Part “Algorithm for the reconstruction of chromosome structures with cubic complexity and sufficient approximation ratio” is as follows. We are given *m* chromosome structures. It is required to find a binary tree *T* with *m* leaves and the given structures assigned to them as well as the arrangement of structures at all internal nodes with the minimum total distance between edge ends for all edges. This sum is called the *weight* of tree *T* or the *weight* of the corresponding arrangement.

The case of a non-binary tree is similar to that of a binary one and presents no new difficulties. The same holds true for the case of an unrooted tree.

Finally, Part “Reconstruction of chromosome structures for mitochondria of sporozoans and plastids of rhodophytic branch” illustrates the algorithms described in Parts “Exact linear algorithm calculating the distance between chromosome structures” and “Algorithm for the reconstruction of chromosome structures with cubic complexity and sufficient approximation ratio” and one more algorithm proposed in [1] by generating phylogenetic trees and reconstructing chromosome structures of mitochondria in sporozoans and plastids of rhodophytic branch. The test on artificial data is available at http://lab6.iitp.ru/en/chromoggl/.

### Results of most relevant works

Let us briefly review the background of the algorithm in Part “Exact linear algorithm calculating the distance between chromosome structures”. Following the work by David Sankoff [16], Pavel Pevzner (reviewed in [17]) and many other researchers addressed the distance problem associated with models of chromosome structures; thousands of papers, books, and lecture courses concerning this problem are available. Analysis of the research in the field deserves a separate extensive survey accounting for fine distinctions between the studied models. Let us consider several publications most relevant to our work. Yancopoulos et al. [18] proposed a set of operations to transform chromosome structures; here they are called standard and are included in our set of operations. This work presents an algorithm to calculate the shortest distance between structures composed of paths only (linear chromosomes); the algorithm computation time tends to linear but was not explicitly evaluated. These operations applicable only to paths correspond to inversion, translocation, fusion, and fission operations defined in [19]. The distance problem for linear chromosomes only was solved in [19]. The general case for the same gene content and the same operation weights was solved in [20]. The case of different gene content requires extra operations, deletion and insertion of special gene loci, which were defined in [7].

The adjacency graph is used in [7, 8]: its nodes are adjacent extremities of genes that belong to both structures as well as extremities of the initial paths. Such nodes are connected by an edge if they include the same gene. In addition, the path extremity is considered as connected to a telomere (an empty end). A region with genes that belong to a single structure (“special” genes) can reside between adjacent extremities of common genes; such genes are assigned to the corresponding node. Such graph clearly differs from the common graph defined here. The algorithms computing the shortest sequence transforming one structure into another using the same operations as in our work proposed in [7, 8] have a linear running time. All operation weights equal 1 in [7], while the standard operation weights equal 1 and the weights of deletions and insertions are the same and do not exceed 1 in [8]. It remains unclear if the algorithms from these works can be related to our algorithm. The proof that the algorithms in [7, 8] are exact is not available to us, and the accompanying notes give no necessary details.

The same model as in our work is considered in [9]. It proposes a linear algorithm that relies on the addition of special genes to both initial structures providing that the operation weights are the same. Thus, the problem is reduced to the case with the same gene content, and the total number of genes increases by *k+t*, were *k* and *t* are the numbers of special genes in the initial structures. The used graph includes an additional pair of extremities for each special gene, which increases their graph relative to that used in our algorithm. The graph and the algorithm differ from those proposed below. The proof that their algorithm is exact is not available to us, and the accompanying considerations give no necessary details.

Compeau [10] describes the generalization of the algorithm in [9] for the case when all chromosomes are circular, standard operation weights equal 1, and the weights of deletions and insertions are the same. The proof of the algorithm exactness as well as the proper description of the algorithm need to be described in full.

## Methods, results and discussion

### Exact linear algorithm calculating the distance between chromosome structures

#### Definition of the model of chromosome structure

The model of chromosome structure is described as a finite set of paths and circles with directed edges including loops. Such set can be considered as a directed graph referred to as a *chromosome structure*. The graph edge represents a *gene*; an individual graph path or circle represents a *chromosome*. Each gene is denoted by name, usually by number *i*, which can be repeated (for paralogs) and takes the form *i.j*. As usual, this model disregards the lengths of genes and intergenic regions as well as their content. The edge direction indicates the strand on which the gene is located. Graph node connects *adjacent* genes irrespective of their orientation, i.e., it *identifies* (or *glues together*) two extremities of adjacent genes. Usual structures include many paths and circles, which leads to a sort of *interaction* between them. That is why the cases with many chromosomes in their structure are in marked contrast with those with a single chromosome.

The model includes *operations* over a chromosome structure; first four of them given in [18, 20] will be referred to as *standard*. Let us recall their definitions. *Double-cut-and-paste* is cutting two pairs of adjacent gene extremities and cut-and-pasting four extremities, which gives rise to a new structure. *Sesqui-cut-and-paste* is cutting two adjacent extremities and joining one extremity to an unconnected extremity so that the other extremity remains *free. Cut-and-join* is cutting two adjacent extremities resulting in two free extremities or, vice versa, joining two free extremities.

We are given chromosome structures *a* and *b*; a gene that belongs to both structures is called a *common* gene, while a gene that belongs to one structure only is a *special* one; an *a-*gene belongs to structure *a*; and a *b-*gene, to structure *b*. Let us introduce two *accessory* operations that transform *a* into *b*: *deletion* of a (longest continuous) region of special *a-*genes and *insertion* of a region of special *b-*genes.

If the deleted region was strictly within a path or circle, the resulting free extremities of common genes are joined; if the region terminated the path, the common gene extremity becomes free; if the region was a separate chromosome, it is naturally deleted. If a (continuous) region is inserted strictly inside a path or circle, the insertion point is cut, which is not a separate operation; insertion can occur into a path end or as a new chromosome. It is easy to prove that deletions of non-longest (continuous) regions of special *a-*genes do not decrease the distance between structures. Similarly, cutting within special *a-*regions, insertion of special *b-*genes into a special *a-*region, and double- and sesqui-cut-and-paste operations resulting in cutting a region of special *a-*genes and its circularization can also be excluded from the first three operations. Such “unnatural” operation variants are not allowed.

Thus, *six operations are given* and each of them is assigned a positive rational number referred to as its *weight*. The inclusion of weights is the key point of the model. The *objective* is to find the *shortest* sequence of these operations that transform structure *a* into structure *b*. Naturally, the shortest sequence has the minimum total weight of all its operations. Each operation in the sequence is considered together with the chromosome structure to which it is applied.

### Reduction of the problem of paralogs to linear programming

For brevity, let us denote *structures with paralogs* as “par-structures” and *structures without paralogs* as “structures.” The distance between two par-structures is defined as the minimum distance between structures resulting from a bijection between paralogs of every gene present in both initial par-structures. Specifically, gene *i* from both par-structures *a* and *b* can have a different number of paralogs that belong to sets *Р*_*а*_(*i*) and *Р*_*b*_(*i*), respectively. Let *f*_*i*_ be the bijection between parts of sets *Р*_*а*_(*i*) and *Р*_*b*_(*i*); *i.j* indices are found for all paralogs in *Р*_*а*_(*i*) and *Р*_*b*_(*i*), for which *f*_*i*_ is the identity function; all paralogs not included in the function *f*_*i*_ domain and range have different indices. Of course, the parts have no repetitive indices. The numbering has natural interpretation: if *y* = *f*_*i*_(*x*), *y* is “inherited” from *x*; other paralogs from *Р*_*а*_(*i*) and *Р*_*b*_(*i*) are independent mutually different genes, and thus they have different *j* indices. Paralogs that do not belong to the domain and range of *f*_*i*_ are considered as *lost* and *emerged*, respectively (on the edge connecting *a* and *b* on the phylogenetic tree). Now the *distance* between *a* and *b* is defined as the minimum distance between *a’* and *b’*, which are derived from *a* and *b* for all specified indices of paralogs from par-structures *a* and *b*.

The calculation of the distance between par-structures is *initially* reduced to integer linear programming (ILP), which is known to give an exact solution with close to linear time and memory complexity for random data [2–4]. This special property of linear, integer, and Boolean programming is formulated as an *almost linear* algorithm; it is considered in numerous publications and is not discussed here. Thus, the solution found by ILP specifies a set of bijections {*f*_*i*_ |*i*} between paralogs. *Then* arbitrary indices of paralogs corresponding under these bijections are selected; the result does not depend on index selection. *Finally*, the algorithm described in the Section “Definition of the common graph and its final form” is applied to calculate the distance between the obtained structures *a’* and *b’*. The reduction to ILP is described in the Sections “Calculation of the breakpoint and biological distances for structures with paralogs” and “Calculation of biological distance with paths present”.

Since the algorithm presented below is linear, the calculation of the distance between par-structures becomes almost linear. The exactness is still observed. Thus, one can assume the absence of paralogs in the remainder of Part “Exact linear algorithm calculating the distance between chromosome structures”.

### Definition of the common graph and its final form

In the common graph *a+b* of two structures *a* and *b*, the nodes are extremities of common genes as well as all longest continuous regions of special genes; each extremity is taken once. In more formal terms, gene extremities are assigned the gene name with indices 1 and 2 for its beginning and end, respectively. Nodes of the first and second types are referred to as *conventional* and *special*, respectively. An edge connects two conventional nodes if the extremities are adjacent in one of structures, i.e., neighbor each other on the chromosome. An edge connects conventional and special nodes if an extremity of the common gene is adjacent to a marginal gene in a region of special genes. The edges in the first and second cases are called *conventional* and *special*, respectively. The marginal edge with a special end in a path from *a+b* is referred to as *hanging*. Edges are denoted *a* or *b* depending on the structure where joining occurred; nodes can be connected by double edges. Special nodes are denoted *a-* or *b-*nodes depending on the source structure. A graph can contain isolated nodes – regions of special genes. If such region is a circle in the initial structure, a loop called *special* is added to the node. This yields an undirected graph referred to as *a+b*.

Analogs of five operations over structures can be applied to the common graph *a+b* as follows (see figures in #1 Section of Additional materials). (1) Delete two non-incident edges with the same index and connect four resulting ends by two new non-incident edges with the same index. (2) Delete an edge (for example, an *a-*edge) and connect one of its ends with a conventional node non-incident to the *a-*edge or with a special *a-*node with no more than one incident *a-*edge. (3) Delete any edge. (4) Use an edge (for example, an *a-*edge) to connect nodes each of which is a conventional and non-incident to the *a-*edge or a special *a-*edge with no more than one incident *a-*edge. If an operation results in two incident special nodes, they are merged (which is a part of the operation); the resulting node is given a name combining those of initial nodes. (5) Delete a special node or a special loop. If this node had two conventional nodes incident to it, they are connected with an edge. An analog of the sixth operation, insertion, is easy to define; however, it turns out that it can be omitted without loss of generality. This is a not trivial statement; see the beginning of the Section “Calculating the distance between structures”.

The final form of the common graph *a+b* is defined as a common graph consisting of isolated conventional nodes and *final 2-circles*. The latter is defined as a graph of two conventional nodes connected by conventional edges, one from *a* and one from *b*. It is easy to show that the initial objective is equivalent to transforming the graph *a+b* into the final form with certain constraints on operation weights, in particular, when operations other than insertion and deletion have the same weight.

### Calculating the distance between structures

Let us recall that this section assumes the absence of paralogs but all operations, different gene content, and any operation weights are allowed. A common graph *a+b* is trivially constructed from initial structures *a* and *b*. The algorithm goal is to transform *a+b* into the final form. Let us denote the *length* of a path or circle by the number of internal special nodes plus the number of conventional edges. For instance, a 2-circle is a circle of length 2.

Having the same framework, the algorithm depends on the proportions between operation weights, which are fixed in advance. The algorithm is the following.

- **Step 1.** Delete all special *a-*loops.
- **Step 2.** Cut out a conventional edge not included in a 2-circle and close it into the final 2-circle using a double- (internal edge) or a sesqui-cut-and-paste (extreme edge) or a join (singular edge) operation. Repeat the operation if possible. If the double-cut-and-paste weight does not exceed that of sesqui-cut-and-paste, all double operations are performed first; otherwise, all sesqui operations go first. Figures shown in #2 Section of Additional materials can be helpful for understanding the algorithm flow.
  Let us explain steps 3 and 4 prior to their formal description. Step 3 uses operations transforming certain combinations of two, three, or four paths into a single path. Each operation applied decrements the number of special nodes by 1. The combinations are specified by the *type* of a path or circle, which is defined below. Step 4 is used if the deletion of a *b-*node has a higher weight compared to all other operations. The current set of paths and circles is split into pairs, joint processing of which replaces the deletion of a *b-*node with a lower weight cut-and-paste operation joining two *b-*nodes; the total number of operations remains unaltered. We have demonstrated that the sequence procedures specified below in steps 3 and 4 provide for the optimal result.
  Let us define the *types*. A path is referred to as *odd* and *even* if its length is odd and even, respectively. *a-*Path denotes an isolated *b-*node or an odd path where extreme non-hanging edges marked as *a*; *b-*path is defined symmetrically. The paths and circles remaining after steps 1 and 2 (excluding the final 2-circles and isolated conventional nodes) are assigned the following *types*: *a-*circle, for a 2-circle containing an *a-*node but not a *b-*node; *b-*circle, vice versa; circle, for a circle including both *a-* and *b-*nodes; loop, for a special *b-*loop.
  *a-*Paths are assigned to the following types: *1а* if the path has a single hanging edge; *2а*, if it has two hanging edges; *2a’*, if it is an isolated special *b-*node; *3а*, if it has no hanging edges but has both *a-* and *b-*nodes (the path length should be strictly greater than 1 in this case); and *3a’*, if there are neither hanging edges nor *b-*nodes. *b-*Path types are defined in a similar way. Even paths are assigned to the following types: *1*, if the path has a single hanging edge and a *b-*node; *1'*, if it has one conventional node and one special *a-*node incident to it; *1"* if it has one conventional node and one special *b-*node incident to it; *2*, if it has two hanging edges and a non-hanging edge; *2'*, if it has only two hanging edges; and *3*, if it has only non-hanging edges. Type *1* is subdivided into types *1*_*a*_ and *1*_*b*_ if the extreme special node is an *a-*node and *b-*node, respectively. Type *2a* is a combination of types *2a* and *2a’*; type *3b*, *3b* and *3b’*; type *1*_*b*_, *1*_*b*_ and *1*″ and type *2*, *2* and *2'*.
  Let us introduce a special type 1_*c*_ corresponding to a *deferred choice* between path types 1_*a*_ and 1_*b*_, which are possible results of the operation. The algorithm stores both results up to steps 4.15–4.23 when a decision on either of two results is made, and thus the whole sequence of operations becomes unambiguously defined.
  In the description of step 3 below, a combination of path types (separated by ‘+’) on the left of ‘=’ is transformed into a combination of path types on the right of ‘=’. The resulting combination omits isolated conventional nodes and final 2-circles; no type is assigned to them. Hereafter, if a substep includes several equations, actions of the first one are described; other substeps are analogous.
- **Step 3.** The algorithm performs the actions described below; each action is repeated as long as it is applicable. Figures in #2 Section of Additional materials can be helpful.
  3.1. 1*a* + 1*b* = 1_*c*_. Cut an extreme non-hanging edge in one of two paths of types 1*a* and 1*b* and join the corresponding special node with the extreme special node of the other path (sesqui-cut-and-paste operation).
  3.2. 2*a* + 3*b* = 1_*b*_, 2*b* + 3*a* = 1_*a*_, 2*b’* + 3*a* = 1_*a*_, 2*b* + 3*a’* = 1_*a*_, and 2*b’* + 3*a’* = 1*'*. Cut an external edge in the 3*b-*path and join the special node with the extreme special node of the 2*a-*path.
  3.3. 2 + 3 = 1_*c*_. Cut an external edge in the 3-path and join the special node with the extreme special node of the 2-path. This results in a path of type 1_*a*_ or 1_*b*_ depending on which of two external edges was cut.
  3.4. 1*b* + 2*a* + 3 = 2 + 3 = 1_*c*_, 1*a* + 2*b* + 3 = 2 + 3 = 1_*c*_, and 1*a* + 2*b’* + 3 = 2 + 3 = 1_*c*_. First carry out the 1*b* + 2*a* = 2 operation (see below) and then the 2 + 3 = 1_*c*_ one.
  3.5. 1*a* + 3*b* + 2 = 3 + 2 = 1_*c*_, 1*b* + 3*a* + 2 = 3 + 2 = 1_*c*_, and 1*b* + 3*a’* + 2 = 3 + 2 = 1_*c*_. First carry out the 1*a* + 3*b* = 3 operation (see below) and then the 2 + 3 = 1_*c*_ one.
  3.6. 1*a* + 2 = 2*a* and 1*b* + 2 = 2b. Cut an external edge in the 1*a-*path and join the special node with the extreme special node of the 2-path.
  3.7. 1*a* + 3 = 3*a* and 1*b* + 3 = 3*b*. Cut an external *b-*edge in the 3-path and join the special node with the extreme special node in the 1*a-*path.
  3.8. 1*a* + 1*a* + 2*b* + 3*b* = 2 + 3 = 1_*c*_, 1*a* + 1*a* + 2*b’* + 3*b* = 2 + 3 = 1_*c*_, 1*b* + 1*b* + 2*a* + 3*a* = 2 + 3 = 1_*c*_, and 1*b* + 1*b* + 2*a* + 3*a’* = 2 + 3 = 1_*c*_. First carry out the 1*a* + 2*b* = 2 and 1*a* + 3*b* = 3 operations; then the 2 + 3 = 1_*c*_ one.
  3.9. 1*a* + 1*a* + 2*b* = 3*a* + 2*b* = 1_*a*_, 1*a* + 1*a* + 2*b’* = 3*a* + 2*b’* = 1_*a*_, and 1*b* + 1*b* + 2*a* = 3*b* + 2*a* = 1_*b*_. First carry out the 1*a* + 1*a* = 3*a* operation (see below); then 2*b* + 3*a* = 1_*a*_ one.
  3.10. 1*a* + 1*a* + 3*b* = 1*a* + 3 = 3*a*, 1*b* + 1*b* + 3*a* = 1*b* + 3 = 3*b*, and 1*b* + 1*b* + 3*a’* = 1*b* + 3 = 3*b*. First carry out the 1*a* + 3*b* = 3 operation; then the 1*a* + 3 = 3*a* one.
  3.11. 1*a* + 1*a* = 3*a* and 1*b* + 1*b* = 3*b*. Join the extreme special nodes of two 1*a-*paths.
  3.12. 1*a* + 2*b* = 2, 1*a* + 2*b’* = 2, and 1*b* + 2*a* = 2. Cut an external edge in the 1*a-*path and join the special node with the extreme special node of the 2*b-*path.
  3.13. 1*a* + 3*b* = 3, 1*b* + 3*a* = 3, and 1*b* + 3*a’* = 3. Cut an external edge in the 3*b-*path and join the special node with the extreme special node of the 1*a-*path.
  3.14. 2*a* + 2*b* + 3 + 3 = 2 + 3 = 1_*c*_ and 2*a* + 2*b’* + 3 + 3 = 2 + 3 = 1_*c*_. First carry out the 2*a* + 2*b* + 3 = 2 operation (hereafter, the descriptions are given below); then the 2 + 3 = 1_*c*_ one.
  3.15. 3*a* + 3*b* + 2 + 2 = 3 + 2 = 1_*c*_ and 3*a’* + 3*b* + 2 + 2 = 3 + 2 = 1_*c*_. First carry out the 3*a* + 3*b* + 2 = 3 operation; then the 2 + 3 = 1_*c*_ one.
  3.16. 2*a* + 3 + 3 = 1*a* + 3 = 3*a*, 2*b* + 3 + 3 = 1*b* + 3 = 3*b*, and 2*b’* + 3 + 3 = 1*b* + 3 = 3*b*. First carry out the 2*a* + 3 = 1*a* operation; then the 1*a* + 3 = 3*a* one.
  3.17. 3*b* + 2 + 2 = 1*b* + 2 = 2*b*, 3*a* + 2 + 2 = 1*a* + 2 = 2*a*, and 3*a’* + 2 + 2 = 1*a* + 2 = 2*a*. First carry out the 3*b* + 2 = 1*b* operation; then the 1*b* + 2 = 2*b* one*.*
  3.18. 2*a* + 2*b* + 3 = 2*a* + 1*b* = 2 and 2*a* + 2*b’* + 3 = 2*a* + 1*b* = 2. First carry out the 2*b* + 3 = 1*b* operation; then the 1*b* + 2*a* = 2 one.
  3.19. 3*a* + 3*b* + 2 = 3*a* + 1*b* = 3 and 3*a’* + 3*b* + 2 = 3*a’* + 1*b* = 3. First carry out the 3*b* + 2 = 1*b* operation; then the 1*b* + 3*a* = 3 one.
- **Step 4.** If the weight of *double-cut-and-paste is greater than that of sesqui-cut-and-paste*, actions 4.1–4.24 are sequentially performed whenever possible; otherwise actions 4.1*'*–4.24*'* specified when they differ from the corresponding actions 4.1–4.24 are performed. Figures in #2 Section of Additional materials illustrating all actions can be helpful.
  4.1. “Loop” + any type *t* with a *b-*node = type *t.* Join the *b-*node of the loop with the *b-*node of *t-*type chromosome by double-cut-and-paste (if this path is not an isolated *b-*node) or by sesqui-cut-and-paste (otherwise).
  4.2. “Circle” + any type *t* with a *b-*node and an *a-*node = type *t.* Insert the circle (by double-cut-and-paste combining two *b-*nodes) near the *b-*node from *t-*type chromosome on the side of the *a-*node; cut out the resulting conventional edge.
  4.3. 2*a* + 2*b* = 2 + 1*'*. Perform the sesqui-cut-and-paste with cutting out two 2*b-*path nodes (the extreme *a-*node and the neighboring conventional node) and joining the resulting extremity with the extreme special *b-*node of the 2*a-*path.
  4.3'. 2*a’* + 2*b* = 2 + 1*'*.
  4.4. 3*a* + 3*b* = 3. Cut an external edge in the 3*a-*path and join the special node with the extreme conventional node of the 3*b-*path.
  4.4'. 3*a* + 3*b’* = 3.
  4.5. 2*a* + 3 = 1*a* and 2*b* + 3 = 1*b*. Cut an external *b-*edge in the 3-path and join the special node with the extreme special node of the 2*a-*path.
  4.5'. 2*a’* + 3 = 1*a*.
  4.6. 3*a* + 2 = 1*a* and 3*b* + 2 = 1*b*. Cut an external edge in the 3*a-*path and join the special node with the extreme special node of the 2-path.
  4.6'. 3*a* + 2*'* = 1*a* and 3*b’* + 2 = 1*b*.
  4.7'. Join the extreme special nodes of the two paths.
  4.7. 2*a’* + 2*a* = 2*a*.
  4.8. 3*a* + 3*a* = 3*a* and 3*b* + 3*b* = 3*b*. Connect two extreme conventional nodes of the paths by a conventional edge, and then cut out this edge.
  4.8'. 3*b’* + 3*b* = 3*b*.
  4.9. 1*a* + 2*a* = 1*a* and 1*b* + 2*b* = 1*b*. Connect the extreme special nodes of the two paths.
  4.9'. 1*a* + 2*a’* = 1*a*.
  4.10. 1*a* + 3*a* = 1*a* and 1*b* + 3*b* = 1*b*. Connect two extreme conventional nodes of the paths by a conventional edge, and then cut out this edge.
  4.10'. 1*b* + 3*b’* = 1*b*.
  4.11. 2*a* + 2 = 2 and 2*b* + 2 = 2. Connect the extreme special nodes of the two paths.
  4.11'. 2*a’* + 2 = 2, 2*a* + 2*'* = 2, and 2*b* + 2*'* = 2.
  4.12. 3*a* + 3 = 3 and 3*b* + 3 = 3. Connect two extreme conventional nodes of the paths by a conventional edge, and then cut out this edge.
  4.12'. 3*b’* + 3 = 3.
  4.13. 2 + 2 = 2 + 1*'*. Perform the sesqui-cut-and-paste operation with cutting out two nodes of the 2-path (the extreme special *a-*node and the neighboring conventional node) and joining the resulting terminus with the extreme special *b-*node of the other 2-path.
  4.13'. 2*'* + 2 = 2 + 1*'*.
  4.14. 3 + 3 = 3. Cut an external *a-*edge in the 3-path and join the resulting extremity with the *b-*extremity of the other 3-path.
  4.14'. Null action.
  4.15. 1_*a*_ + 1_*a*_ = 1_*a*_, 1_*b*_ + 1_*b*_ = 1_*b*_, and 1_*b*_ + 1_*c*_ = 1_*b*_ (set *c* = *b*). Cut an external non-hanging edge in the 1_*a*_*-*path and join the special node with the extreme special node of the other 1_*a*_*-*path.
  4.15'. 1*"* + 1_*b*_ = 1_*b*_ and 1*"* + 1_*c*_ = 1_*b*_ (set *c* = *b*).
  4.16. 1*a* + 1_*b*_ = 1*a*, 1*b* + 1_*a*_ = 1*b*, and 1*a* + 1_*c*_ = 1*a* (set *c* = *b*). Cut an external non-hanging edge in the 1_*b*_*-*path and join the special node with the extreme special node of the 1*a-*path.
  4.16'. 1*a* + 1*"* = 1*a*.
  4.17. 1*a* + 1_*a*_ = 1*a*, 1*b* + 1_*b*_ = 1*b*, and 1*b* + 1_*с*_ = 1*b* (set *c* = *b*). Cut an external edge in the 1*a-*path and join the special node with the extreme special node of the 1_*a*_*-*path.
  4.17'. 1*b* + 1*"* = 1*b*.
  4.18. 2*a* + 1_*b*_ = 2*a*, 2*b* + 1_*a*_ = 2*b*, and 2*a* + 1_*c*_ = 2*a* (set *c* = *b*). Cut an external non-hanging edge in the 1_*b*_*-*path and join the special node with the extreme special node of the 2*a-*path.
  4.18'. 2*a’* + 1_*b*_ = 2*a*, 2*a* + 1*″* = 2*a*, and 2*a’* + 1_*c*_ = 2*a* (set *c* = *b*).
  4.19. 3*a* + 1_*a*_ = 3*a*, 3*b* + 1_*b*_ = 3*b*, and 3*b* + 1_*c*_ = 3*b* (set *c* = *b*). Cut an external edge in the 3*a-*path and join the special node with the extreme special node of the 1_*a*_*-*path.
  4.19'. 3*b’* + 1_*b*_ = 3*b*, 3*b* + 1*"* = 3*b*, and 3*b’* + 1_*c*_ = 3*b* (set *c* = *b*).
  4.20. 2 + 1_*a*_ = 2, 2 + 1_*b*_ = 2, and 2 + 1_*c*_ = 2 (set *c* = *b*). Cut an external non-hanging edge in the 1_*a*_*-*path and join the special node with the extreme special node of the 2-path.
  4.20'. 2*'* + 1_*a*_ = 2, 2*'* + 1_*b*_ = 2, 2 + 1*"* = 2, and 2*'* + 1_*c*_ = 2 (set c = b).
  4.21. 3 + 1_*a*_ = 3, 3 + 1_*b*_ = 3, and 3 + 1_*c*_ = 3 (set *c* = *b*). Cut an external edge in the 3-path and join the special node with the extreme special node of the 1_*a*_*-*path.
  4.21'. 3 + 1*"* = 3.
  4.22. 1_*a*_ + 1_*c*_ = 1_*a*_ (set *c* = *a*), 1*b* + 1_*c*_ = 1*b* (set *c* = *a*), 1*a* + 1_*с*_ = 1*a* (set *c* = *a*), 2*b* + 1_*c*_ = 2*b* (set *c* = *a*), and 3*a* + 1_*c*_ = 3*a* (set *c* = *a*).
  4.22'. Null action.
  4.23. For the remaining paths of type 1_*c*_, set *c* = *b* and perform the 1_*b*_ + 1_*b*_ = 1_*b*_ operation.
  4.23'. Null action.
  4.24. Paths with a non-hanging edge are closed into circles by joining (path types 2*a*, 2*b*, 3*a*, and 3*b*), sesqui-cut-and-paste with merging the special nodes (path types 1_*a*_, 1_*b*_, 1_*c*_, and 2) or without it (path types 1*a*, 1*b*, and 3). Set *c* = *b* after closing a path of type 1_*c*_. When closing a path of type 2, select the variant with merging two *b-*nodes and delete the *a-*node from the resulting path of type 1*'*. Cut out conventional edges from the circles resulting from closing paths of type 3*a* or 3*b*. Then execute step 4.2 again.
- **Step 5.** Delete isolated special nodes and loops. Delete special notes from the remaining paths. Cut out 2-circles from circles longer than 2 so that two *b-*nodes merge (thus, the *a-*node is included into the 2-circle; see figure in #2 Section of Additional materials). Delete special nodes from 2-circles.

### Proof of the algorithm exactness

Clearly, the time and memory complexity of the algorithm are linear. Its exactness is demonstrated using the following sequence of lemmas 1–8 and the constraint presented in the following subsection; certain details can be found in [5, 6].

For initial structures *a* and *b*, the shortest sequence of operations transforming *a* into *b* is referred to as the *shortest sequence*.

1. There exists the shortest sequence with no operations that include cutting a region of *a-*special genes. This is proved by induction on the weight of such sequence.
2. There exists the shortest sequence subject to the previous condition where all deletions precede all insertions. Accordingly, the common graph transformation into the final form can be done without the insertion operation. This is proved by displacement of all operations decreasing the number of special nodes to the beginning of the sequence.
3. The sequence of operations specified in step 3 provides the greatest possible saving in the number of operations relative to processing each path individually. This is proved by induction on actions in step 3: the resulting sequence can be continued upon the completion of each action as long as such saving remains possible. Thereafter, the path or circle is called a *component*.
4. After an action in step 4 is executed, it cannot be applied again (except 4.2 in 4.24), i.e., further actions do not introduce any components to which this action can be applied. This is proved by enumerating actions in step 4.
5. After step 4, there are 0, 1, or 2 components with a *b-*node excluding the initial circles with a *b-*node but no *a-*node. This is proved by contradiction: if there were more than two such components, they would be subject to one of actions in step 4, which contradicts the previous lemma.
6. The sequence of actions specified in step 4 provides for the greatest possible number (with an accuracy of 1) of replacements of high-weight *b-*node deletion operation with another one. This follows from the previous lemma and from the opportunity to transform any component with *b-*nodes into the final form using a sequence containing exactly one operation of *b-*node deletion.
7. The total weight *C* of a sequence of operations generated by the algorithm is derived from readily calculated properties of the initial common graph. Namely, C = *B* + *S* + *D*–*P* + *ε*(*B’* + *n*), where *B* is the number of special nodes, *S* is the sum of integer parts of half-lengths of the longest regions composed of conventional edges plus the number of such boundary regions of odd length minus the number of such circular regions, *D* is the number of operations not decreasing the number of special nodes that are required for the transformation of odd chromosomes individually into the final form excluding the operations in step 2 (this number can be determined for any chromosome from its type), *P* is the number of operations saved in step 3, *ε* is the weight of *b-*node deletion minus 1, *B’* is the number of circles with a *b-*node but with no *a-*nodes, and *n* is 0, 1, or 2.
8. Induction on the total weight *M* of the shortest sequence is used to prove that *C* = *M*. Inductive step: for any operation *o* applied to any common graph *G*, its weight is at least *C*(*G*)–*C*(*o*(*G*)), where *C*(*G*) is defined for a given *G* as in the previous lemma. This is proved by enumerating all operations and types of paths and circles to which they are applied.

### Condition for the exactness of the algorithm and operation weight values

In practical computations we considered two weight patterns referred to as *circular* and *linear* applied to plastids and mitochondria, respectively. When passing from *a* to *b*, the patterns are defined by any inexact descent in operation weights in the following order. *Circular* pattern: *b-*node deletion, sesqui-cut-and-paste, *a-*edge insertion or *b-*edge deletion, double-cut-and-paste, and *a-*node deletion. *Linear* pattern: *b-*node deletion, double-cut-and-paste, sesqui-cut-and-paste, *a-*edge deletion or *b-*edge insertion, and *a-*node deletion. The sequence of transformations from *a* to *b* has the *minimum length* when the length is minimal among all possible sequences of transformations from *a* to *b*.

The proposed algorithm is exact in two cases: (1) when the given structures have the same gene content and the weight pattern is cyclic or linear (the algorithm is exact for sequences of minimal length) and (2) when inequality *d*≤*c* ≤2*d* is satisfied for the weight *c* of *b-*node deletion and identical weights *d* of other operations.

In case (2), the total weight for the sequence of operations generated by the above algorithm differs from the total weight of the shortest sequence by no more than *d*; this uncertainty stems from the accuracy to 1 to which the number of operation replacements is maximized at step 4.

In the case of arbitrary weights in the range from 0.8 to 1.5, which we actually used, the algorithm becomes heuristic although the deflection of its result from the minimum solution is no more than 1.5 times according to our tests (data not shown).

Part “Reconstruction of chromosome structures for mitochondria of sporozoans and plastids of rhodophytic branch” considers circular and linear patterns of operation weights with the following weight values (the order of operations is pattern-specific): 1.5, 1.2, 1.1, 1, 0.9, and 0.8. Clearly, computations for other weight values and routine analysis of the obtained results are of interest; however, this will generate an enormous volume of data.

### Algorithm simulation example

**Fig. 1:**
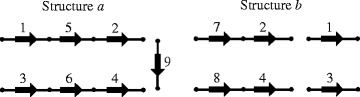
Two chromosome structures

Let us consider structures *a* and *b* shown in Fig. 1.

**Fig. 2:**
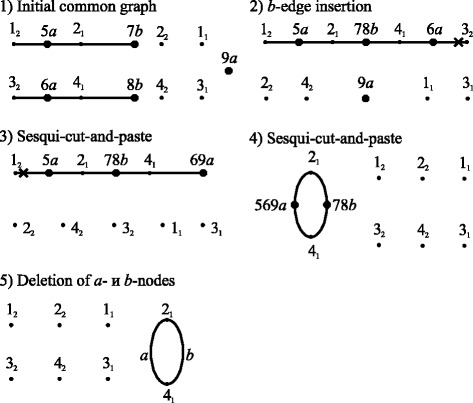
Sequence of structures and operations generated by the algorithm for the example shown in Fig. 1

Figure 2 shows the sequence of structures and operations generated by the algorithm to transform the common graph *a+b* of structures *a* and *b* shown in Fig. 1 into the final form.

**Fig. 3:**
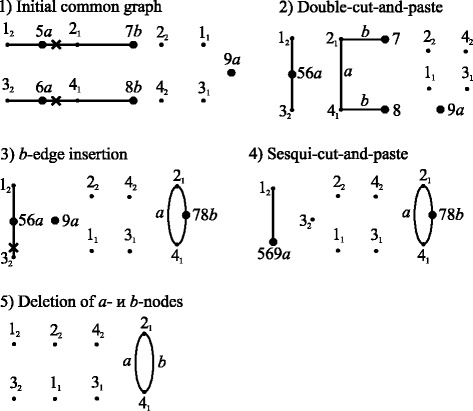
Shortest sequence of structures and operations in the case of circular weights for the example shown in Fig. 1

The algorithm generates this sequence for any operation weights. This sequence is the shortest for linear weights with the total weight of 5.4. For circular weights, this sequence is not the shortest; its total weight is 5.7, while that of operations shown in Fig. 3 is 5.4.

### Algorithm for the reconstruction of chromosome structures with cubic complexity and sufficient approximation ratio

Approximate algorithms solving the reconstruction problem with the approximation ratio of 2 and 11/6 have been developed for the breakpoint and biological distances between chromosome structures. The two developed algorithms have a cubic and a quintic polynomial complexities, respectively. These two algorithms and the respective proofs are presented below. Let *n* be the total number of genes (in other words, gene names or numbers) in a given set of *m* structures.

Just as Alon et al. [15], we reduce the reconstruction problem to the minimum Steiner tree problem. Let us remind it. Given an undirected graph *G* with non-negative numbers (“weights”) assigned to its edges; *m* nodes of the graph called *terminals* are fixed. It is necessary to find a connected tree *S* (possibly unrooted and non-binary) in *G* that includes all terminals and has the minimum sum of numbers assigned to all edges in *S*. This sum is called the *weight of tree S*.

In order to *solve the reconstruction problem, let us consider G* as a complete graph of all chromosome structures with no more than *n* genes, where each edge is assigned the distance between two structures at its ends. The nodes corresponding to *m* given structures will be taken as terminals. The following lemma establishes a correspondence between the reconstruction problem and the Steiner problem for the specified *G*.

#### Lemma

Any solution *T* of the reconstruction problem with linear complexity can be transformed into a solution *S* of the Steiner problem, and the weight of *S* is no more than the weight of *T*. Any solution *S* of the Steiner problem with the same complexity can be transformed into a solution (the proper *T* and the arrangement for it) of the reconstruction problem, and the weight of *T* is no more than the weight of *S*.

#### Proof

Forward. Let us join every set of *T*-nodes with identical labels into a single node. Eliminate circles, e.g., by generating any spanning tree in the derived graph, and the result is isomorphically embedded in *G*.

Backward. If there is a node of degree 2 in *S*, let us take it as a root; otherwise let us add a root node to one edge and assign a structure of one of its ends to it. Let us add an incident edge to each terminal non-leaf node in *S* with the same structure at the end, which gives us a leaf in *T*; this ensures that all given structures are present in the leaves of *T*. To generate the proper *T*, let us remove non-terminal leaves in *S* together with edges incident to them and non-root nodes of degree 2, and then join the edges. Each non-binary node is arbitrarily binarized, and the structure assigned to it is assigned to new nodes. This gives us a binary tree *T* with the weight not exceeding that of *S*.

Two algorithms are known to solve the Steiner problem, and each of them can be transformed to solve the reconstruction problem.

### The first algorithm solving the reconstruction problem for structures without paralogs

This algorithm is faster but has the *approximation ratio* of 2 owing to the corresponding solution of the Steiner problem [21]. The second one is slower but has the *approximation ratio* of 11/6; it is considered in the Section “The Second Algorithm Solving the Reconstruction Problem for Structures without Paralogs” below.

Let us recall the first algorithm solving the Steiner problem. It generates the complete graph *G’*, whose nodes are *m* given terminals, and each edge (*u,v*) is labeled by the length of the shortest path between *u* and *v* in the initial graph *G*. A minimum spanning tree is generated for *G’*. Each edge in it is replaced with some shortest path in *G*. The circles are eliminated by removing the edges, and the resulting tree is the solution of the Steiner problem. The *approximation ratio* of the algorithm is 2, i.e., the weight of the solution differs from the minimum one by a factor of no more than 2.

Our first algorithm operates on the graph *G* that was used in the Lemma. It has a number of nodes exponential in *n*, but it does not have to be processed completely: only *m* chromosomal structures are needed. Indeed, it follows from the triangle inequality that the shortest path between the vertices in *G* is the edge connecting them. Accordingly, plotting the graph *G’* is equivalent to computing a distance matrix for initial *m* structures. Considering that the computation of the breakpoint distance between structures *without paralogs* has a linear complexity on the size of structures, the matrix can be computed in *nm*^*2*^ steps. The minimum spanning tree in a graph with *m* nodes is generated using the Prim’s algorithm in *m*^*2*^ steps. Thus, the proposed algorithm has the complexity of *nm*^*2*^ and the *approximation ratio* of 2.

The biological distance between structures *a* and *b* is asymmetrical, i.e., the distance from *a* to *b* can be not equal to the distance from *b* to *a*. Thus, hereafter, *biological distance* denotes the mean of these two distances. All properties of biological distance specified above remain unaltered in this case. If an edge to which structures *a* and *b* are assigned is directed, i.e., the edge is in a rooted tree and *a* is closer to the root, the biological distance for the edge is computed from *a* to *b* and is called the *one-way* biological distance.

According to our data (see Part “Exact linear algorithm calculating the distance between chromosome structures” and [5, 6]), the computation of the biological distance between structures without paralogs also has a linear complexity. Consequently, our first algorithm and the conclusions concerning it remain unaltered for the biological distance.

### Calculation of the breakpoint and biological distances for structures with paralogs

If there are paralogs and the breakpoint distance is considered, its computation is reduced to a Boolean linear programming as implemented in the reconstruction of chromosome structures along the tree in [22]. In this case, the tree includes a single edge with chromosome structures at its ends. From this, the algorithm complexity is easy to evaluate; the approximation ratio remains unaltered.

Assume that there are paralogs and the biological distance is considered. Let us show how its computation can be reduced to Integer linear programming *if all chromosomes are circular* and *operation weights are equal*. The first limitation is lifted in the following section, while the second one remains in effect through the end of this Part “Algorithm for the reconstruction of chromosome structures with cubic complexity and sufficient approximation ratio”.

Hence, the common graph includes only circles, and the length of the shortest path equals *B*+*S*_1_–*S*_2_, where *B* is the number of *blocks* (i.e., special nodes) in the common graph, *S*_1_ is the sum of integer parts of half of lengths of maximum (by inclusion) regions (in circles) composed of conventional edges, and *S*_2_ is the number of circles composed of conventional edges [1, 5]. The following reduction to the specified programming is used to compute the summands *B*, *S*_1_, and *S*_2_. We will call a pair of adjacent gene extremities an *adjacent pair*.

Let *a* and *b* be two given chromosomal structures. Let us define a Boolean variable *z*_*kij*_; it equals 1 if paralog *i* of gene *k* in structure *a* corresponds to paralog *j* of the same gene *k* in structure *b*, otherwise it equals 0. The constraints for these variables are that the sums of variables over the third index are no more than 1 for any fixed *k* and *i* (or *k* and *j*).

1. Calculation of *B*. Each adjacent pair *s* in structure *a* is described by a Boolean variable *x*_*as*_; it equals 1 if this pair is at the border of an *a-*block, otherwise it equals 0. A similar description holds for structure *b*. The respective constraints are that if an extremity of paralog *i*_1_ of gene *k* is adjacent to an extremity of paralog *i*_2_ of gene *l* in *s*, then ${x}_{as}\ge {\displaystyle \sum_j{z}_{ki1j}-{\displaystyle \sum_j{z}_{li2j}}}$ and ${x}_{as}\ge {\displaystyle \sum_j{z}_{li2j}-}{\displaystyle \sum_j{z}_{ki1j}}$; similar inequalities hold for pairs in *b*. These inequalities mean that if ${\displaystyle \sum_j{z}_{ki1j}}$ and ${\displaystyle \sum_j{z}_{li2j}}$ are not equal, i.e., a common and a special genes are adjacent in *s*, then *x*_*as*_ = 1.
  Let us define the minimized function as $F=0.5\cdot {\displaystyle \sum_s\left({x}_{as}+{x}_{bs}\right)+\dots }$, where other summands are described below. If ${\displaystyle \sum_j{z}_{ki1j}}$ equals ${\displaystyle \sum_j{z}_{li2j}}$, then *x*_*as*_ = 0, since *x*_*as*_ and *x*_*bs*_ are summands of *F* with the positive coefficient. Each block is assigned two boundary variables so that the sum of *x*_*as*_ and *x*_*bs*_ with coefficient of 0.5 equals *B*.
2. Calculation of *S*_1_. Each adjacent pair *s* in structure *a* is described by a Boolean variable *y*_*as*_; it equals 0 if this pair is at the border or within a block. A similar description holds for structure *b*. For adjacent pairs of common genes, variables *y*_*as*_ and *y*_*bs*_ take on alternating values of 0 and 1 within each region of conventional edges, and this alternation starts from 0 at one of borders. Let us describe the constraints.
  Two adjacent pairs are defined as *potential neighbors* (as edges in the common graph) if they belong to different structures and include the same extremity of paralogs of the same gene. The following constraints are imposed for any potentially neighboring *s*_1_ (in *a*) and *s*_2_ (in *b*): ${y}_{as1}\le 4-{z}_{kij}-{\displaystyle \sum_j{z}_{k1i1j}}-{\displaystyle \sum_j{z}_{k2ji2}-{y}_{bs2}}$ and ${y}_{bs2}\ge {z}_{kij}+{\displaystyle \sum_j{z}_{k1i1j}}+{\displaystyle \sum_j{z}_{k2ji2}-2-{y}_{as1}}$, where paralog *i* of gene *k* is adjacent to paralog *i*_1_ of gene *k*_1_ in *s*_1_, and paralog *j* of gene *k* is adjacent to paralog *i*_2_ of gene *k*_2_ in *s*_2_. These inequalities mean that the values of *y*_*as*_ and *y*_*bs*_ alternate at each region of conventional edges.
  Let us continue the definition: $F=\cdots +{\displaystyle \sum_s\Big({y}_{as}+{y}_{bs}}\Big)+\cdots$, where other summands are described above and below. At the borders of regions with odd length composed of conventional edges as well as within or at the borders of a block, variables *y*_*as*_ and *y*_*bs*_ equal 0, since they are summands of *F* with the positive coefficient. Thus, the sum of all variables *y*_*as*_ and *y*_*bs*_ equals *S*_1_.
3. Calculation of *S*_2_. Each adjacent pair *s* in structures *a* and *b* is described by an integer (rather than Boolean) variable *u*_*s*_ limited by the inequality *u*_*s*_ ≤ *m*_*s*_, where *m*_*s*_ takes values from 1 to the total number of adjacent pairs in *a* and *b*. Let us also introduce a Boolean variable *p*_*s*_ limited by the inequality *p*_*s*_*m*_*s*_ ≤ *u*_*s*_, which indicates whether *u*_*s*_ takes the (maximum possible) value of *m*_*s*_.

Let us continue the definition: $F=\dots -{\displaystyle \sum_s{p}_s}$, where other summands are described above; *p*_*s*_ variables are terms of *F* with the negative coefficient; hence, if *u*_*s*_ equals *m*_*s*_, then *p*_*s*_ = 1. Let us add the constraints for each adjacent pair *s* with a paralog *i* of gene *k* in *a*: ${u}_s\le {m}_s{\displaystyle \sum_j{z}_{kij}}$ as well as similar constraints ${u}_s\le {m}_s{\displaystyle \sum_j{z}_{kji}}$ for adjacent pairs in *b*. These inequalities ensure that *u*_*s*_ = 0 if *s* is within or at the border of a block. Let us add the constraints for any potentially neighboring adjacent pairs *s*_1_ in *a* and *s*_2_ in *b*: *u*_*s*1_ ≤ *u*_*s*2_ + *m*_*s*1_∙(1–*z*_*kij*_) and *u*_*s*2_ ≤ *u*_*s*1_ + *m*_*s*2_∙(1–*z*_*kij*_), where *s*_1_ includes paralog *i* of gene *k* and *s*_2_ includes paralog *j* of gene *k*. These inequalities ensure that *u*_*s*1_ = *u*_*s*2_ for two neighboring edges *s*_1_ and *s*_2_ of the common graph. Accordingly, variables *u*_*s*_ take the same value, and exactly one of these variables reaches its maximum for each circle of conventional edges. For circles that contain blocks, these variables equal zero so neither of them reaches its maximum. Thus, the number of variables *u*_*s*_ that reach their maximum (and equal to the sum of variables *p*_*s*_) equals *S*_2_.

Let us evaluate the number of variables and the number of limitations by an example: let each structure include genes with numbers from 1 to 200 and each gene has 5 paralogs; thus, each structure has 1000 genes. Then the number of variables *z*_*kij*_ is 5000 and there are 2000 constraints for them. The number of variables *x*_*as*_ does not exceed 1000 and there are no more than 2000 constraints for them. The same is true for *x*_*bs*_. The total number of variables *y*_*as*_ and *y*_*bs*_ does not exceed 2000 and there are no more than 4000 constraints for them. The total number of variables *u*_*s*_ and *p*_*s*_ does not exceed 4000 and there are no more than 10000 constraints for them. Overall, no more than 13000 variables and no more than 20000 constraints are introduced, so that the volume of data can be processed by integer linear programming packages. In particular, such task is executed by the Lomonosov supercomputer at Moscow State University.

### Calculation of biological distance with paths present

If structures *a* and *b* include paths in addition to circles, the following approximate (possibly with a high *approximation ratio*) algorithm is used. Let us close all paths in *a* into circles; the resulting structure will be referred to as *a’*. Apply the above algorithm to calculate the distance between structures *a’* and *b’*. The obtained distance added to the total number of paths in *a* and *b* is the algorithm output, which should be close to the distance between the initial *a* and *b*.

Let us evaluate the accuracy of this algorithm. Suppose *n*_1_ and *n*_2_ are the numbers of paths in *a* and *b*, respectively; while *t* and *t’* are the minimum numbers of operations to transform *a* into *b* and *a’* into *b’*, respectively. It follows from the triangle inequality that *n*_1_ + *t* + *n*_2_ ≥ *t’* and *n*_1_ + *t’* + *n*_2_ ≥ *t*, then *t’* ≤ *n*_1_ + *n*_2_ + *t* and *n*_1_ + *t’* + *n*_2_ ≤ *t* + 2(*n*_1_ + *n*_2_). The number of operations that the algorithm needs exceeds the minimum number of operations by no more than 2(*n*_1_ + *n*_2_).

#### Remark

Martinez et al. [23] presented the calculation of the biological distance between two chromosome structures when chromosomal deletions and insertions are not allowed. It is reduced to integer linear programming, and it is assumed that two structures can have different gene content but special genes are ignored after bijections *f*_*i*_ are established between paralogs. Each pair of paralogs *k.i* and *k.j* of gene *k* from different structures is given a similarity value *s*(*k.i*,*k.j*), which ranges from 0 to 1. The minimized function is augmented with a penalty for incomplete similarity between paralogs, which equals the sum of 1–*s*(*k.i*,*k.j*) for all pairs of paralogs *k.i* and *k.j* with established bijections. Paralogs can correspond to each other only if the similarity between them is strictly positive, and there are no two free (i.e., with no correspondence) paralogs with a positive similarity. In our terms, these ideas can be easily realized by supplementing *F* with the sum of *z*_*kij*_∙(1–*s*(*k.i*,*k.j*)) for all pairs of paralogs *k.i* and *k.j*, and by adding the constraints that *z*_*kij*_ = 0 if *s*(*k.i*,*k.j*) = 0 and that ${\displaystyle \sum_{j\hbox{'}}{z}_{kij\hbox{'}}+{\displaystyle \sum_{i\hbox{'}}{z}_{ki\hbox{'}j}\ge 1}}$ if *s*(*k.i*,*k.j*) > 0.

### The second algorithm solving the reconstruction problem for structures without paralogs

Essentially, it is the Zelikovsky’s algorithm, which has the approximation ratio of 11/6 [24] as completely proved elsewhere [25]. First, recall the Zelikovsky’s algorithm. For an arbitrary graph *G*, define *t*(*G*) as the weight of minimum spanning tree in it, and *G*[*z*] is a graph derived from *G* by zeroing the numbers on any two out of three edges connecting the nodes that belong to *z*, where *z* is a set of three nodes in *G*.

- **Step 1**. Build a complete graph *G’* where *m* given terminals are the nodes and each edge (*u*,*v*) is labeled by the length of the shortest path from *u* to *v* in graph *G.*
- **Step 2**. For each triplet *z* = {*a*,*b*,*c*} of different terminals in graph *G*, a node *v*(*z*) with the minimum sum *d*(*z*) of labels on edges (*z*,*a*), (*z*,*b*), and (*z*,*c*) is sought.
  An empty set *A* is defined.
- **Step 3**. It is required to find triplet *z* of terminals with the maximum value of *w* = *t*(*G’*)–*t*(*G’*[*z*])–*d*(*z*). If *w* ≤ 0, then go to step 4. Otherwise take graph *G’*[*z*] as *G’*, add node *v*(*z*) to *A*, and return to step 3.
- **Step 4**. Build a complete graph *G”* that extends *G’*. Its nodes include *m* given terminals and all nodes from *A*, and each edge (*u*,*v*) is labeled by the shortest distance from *u* to *v* in graph *G.* A minimum spanning tree is generated in *G”*. Each edge in it is replaced with some shortest path in graph *G*. Circles are eliminated by removing edges; and the resulting tree is a solution of the Steiner problem.

Let us follow these steps to describe our algorithm solving the reconstruction problem for the breakpoint distance. The distance matrix is computed at steps 1 and 4. At step 2, the structure *v*(*z*) is generated and *d*(*z*) is calculated using our algorithm [1], which arranges chromosome structures at the ancestral nodes of the given tree to minimize the sum of breakpoint distances between structures for all edges of the tree. Namely, it is applied to the tree with three leaves originating from a common ancestor (root). The structures from the triplet *z* are identified among leaves. The structure *v*(*z*) is assigned to the root. The time of calculation of *v*(*z*) and *d*(*z*) for a single triplet *z* is linear in *n* [1].

Let us evaluate the maximum cardinality of the set *A* that does not exceed the maximum number of iterations at step 3 and also evaluate the number of these iterations.

It is easy to show that if graph *G*_2_ is produced from graph *G*_1_ by zeroing the label at edge *e*, the minimum spanning tree *T*_2_ for *G*_2_ can be derived from the minimum spanning tree *T*_1_ for *G*_1_ in the following way. If *e* belongs to *T*_1_ or already has a zero label, then *T*_2_ = *T*_1_. Otherwise, let us consider the only circle in *T*_1_ + *e*, select the edge *e’* with the greatest label, and replace the edge *e’* with *e* in *T*_1_, which gives us the tree *T*_2_; this circle is identified by depth-first traversal of *T*_1_ within time linear in *m*. At each iteration, the labels of two edges of the graph are zeroed, and at least one of these edges has a positive label (since *w* > 0). Thus, each iteration adds at least one edge to the spanning tree of graph *G’*, which remains there until the end of step 3. Since the number of edges in the spanning tree does not exceed the number of nodes *m*, |*A*| ≤ *m* and the number of iterations does not exceed *m*.

Each next spanning tree is derived from the previous one by edge replacement described above. Consequently, we have the following estimates of the proposed algorithm complexity: *nm*^2^ at step 1, *nm*^3^ at step 2, *m*^5^ at step 3, and *nm*^2^ at step 4. The total complexity evaluates to *m*^3^∙max(*n*,*m*^2^). Since the modification concerned the way how steps are taken but not their results, the approximation ratio of 11/6 is preserved.

If the biological distance is used instead of the breakpoint one, steps 1 and 4 are executed in the same manner due to our linear algorithm calculating the distance between two structures; step 3 also remains unaltered.

In this case, a problem emerges at step 2. We are unaware of an exact polynomial algorithm finding structure *d* for structures *a*, *b*, and *c* with the minimum sum of distances between *d* and these structures even for the same gene content. That is why the node *v*(*z*) and the value *d*(*z*) are calculated heuristically, which makes the whole algorithm heuristic. This is the first heuristic algorithm proposed in this paper. Specifically, the shortest path of operations is found to transform *a* into *b* and to transform every intermediate structure into *c*. Symmetry operations are executed for pairs (*a*,*c*) and (*b*,*c*). The structure with the minimum total distance to *a*, *b*, and *c* is chosen as *d* among all encountered structures.

### The case of paralogs

For the breakpoint distance, the problem of searching the node *v*(*z*) as well as the problem of calculating the distance between two structures is reduced to the problem of Boolean linear programming in a manner similar to reconstruction of chromosome structures along the tree [22]. In the present case, the tree consists of three edges going from the root, and chromosome structures *a*, *b*, and *c* are assigned to the leaves. The algorithm generates the ancestral structure *v*(*z*) in the root.

For the biological distance, the problem of searching the node *v*(*z*) becomes more complex. The result obtained for the breakpoint distance can be improved by local rearrangements using the descent algorithm in [1]. But we start from the exact breakpoint decision [22].

To our knowledge, these are the first algorithms with such exactness and polynomial complexity. The test on artificial data is available at http://lab6.iitp.ru/en/chromoggl/.

### Reconstruction of chromosome structures for mitochondria of sporozoans and plastids of rhodophytic branch

This part illustrates the algorithms from Parts “Exact linear algorithm calculating the distance between chromosome structures” and “Algorithm for the reconstruction of chromosome structures with cubic complexity and sufficient approximation ratio” as well as the descent algorithm from [1] using the data on mitochondrial and plastid chromosome structures. The algorithm from Part “Exact linear algorithm calculating the distance between chromosome structures” works adequately with any data, while the algorithm from Part “Algorithm for the reconstruction of chromosome structures with cubic complexity and sufficient approximation ratio” presumably does not always give the correct answer with complex data. This can be attributed to the twofold difference from the minimum solution, which can distort the tree topology in some cases. We used the descent algorithm mentioned above as an alternative to the algorithm from Part “Algorithm for the reconstruction of chromosome structures with cubic complexity and sufficient approximation ratio”. It is of interest that, despite the absence of many usual tools (removal of long branches and less informative columns in the supermatrix, stochastic evolutionary models, bootstrap, etc.), these algorithms generate sensible reconstructions. We considered relatively close mitochondrial genomes as an example of simple data; and quite distant plastid genomes with intricate organization, as complex data.

Let us recall the descent algorithm from [1]. An evolutionary tree is generated based on its similarity to the matrix of pairwise distances as described in the beginning of the Section “Reconstruction of chromosome structures”. The proper reconstruction first finds the arrangement of chromosome structures at internal nodes of the generated tree with the minimum sum *F* of breakpoint distances between the structures at the edge ends for all edges. This is performed using the exact algorithm described in detail in [1], in the absence of paralogs. Boolean linear programming is used in the presence of paralogs [22]. Then, the obtained arrangement is sequentially improved with reference to the biological distance. Namely, all operations applicable to a given structure assigned to an internal tree node are searched through to find the operation and structure that provide for the greatest decrease in the sum (for all edges) *G* of biological distances between structures at the edge ends, and the result replaces the initial structure at the node. This yields the next arrangement. The process is repeated until the sum *G* reaches the minimum, and the algorithm outputs the final arrangement. Its computer implementation is available at http://lab6.iitp.ru/en/chromoggl/.

In the case of paralogs, their coordination among all edges is used for the calculation of tree distance as well as for tree reconstruction.

### Protein clustering algorithm and data

All data were obtained from GenBank. Proteins were clustered using the algorithm described elsewhere [26–28] with the parameters *E* = 0.001, *L* = 0, and *H* = 0.6. Orthology of genes was determined from thus obtained clustering. The database of plastid and mitochondrial protein clusters is available at http://lab6.iitp.ru/ppc/redline67/. Genome compositions were checked by BLAST alignments and Rfam database search.

The evolution of *mitochondrial chromosome structure* was studied in the sporozoan class Aconoidasida composed of subclasses Haemosporida and Piroplasmida (Table 1). Here, even closely related species can have linear and circular chromosomes (Table 1, column 4). No paralogs were found in the considered mitochondria.

**Table 1:** Mitochondrial chromosome structures in the class Aconoidasida

Subclass	Species	Locus in GenBank	Type	Composition
Haemosporida	*Leucocytozoon fringillinarum*	FJ168564.1	C	ss3 ls3 ls9 ss2 ls4 *cox3 ls8 ss5 ss1 cox1 cytb ls1 ss6 ls7 ss4
	*Leucocytozoon majoris*	FJ168563.1	C	ss3 ls3 ls9 ss2 ls4 *cox3 ls8 ss5 ss1 cox1 cytb ls1 ss6 ls7
	*Leucocytozoon sabrazesi*	NC_009336.1	L	ls1 ss4 ss6 ls7 ls6 ss3 ls3 ls9 ss2 ls4 ls5 *cox3 ls8 ss5 ss1 cox1 cytb ls2
	*Plasmodium berghei*	NC_015303.1	L	ls1 ss4 ss6 ls7 ls6 ss3 ls3 ls9 ss2 ls4 ls5 *cox3 ls8 ss5 ss1 cox1 cytb ls2
	*Plasmodium falciparum*	NC_002375.1	L	ss3 ls3 ls9 ss2 *cox3 ls8 ss5 ss1 cox1 cytb ls1 ss4 ss6 ls7
	*Plasmodium floridense*	NC_009961.2	L	ss3 ls3 ls9 ss2 ls4 *cox3 ls8 ss5 ss1 cox1 cytb ls1 ss4 ss6 ls7
	*Plasmodium fragile*	AY722799.1	C	ls1 ss6 ls7 ss3 ls3 ls9 ss2 ls4 *cox3 ls8 ss5 ss1 cox1 cytb
	*Plasmodium gallinaceum*	NC_008288.1	L	ls1 ss4 ss6 ls7 ls6 ss3 ls3 ls9 ss2 ls4 ls5 *cox3 ls8 ss5 ss1 cox1 cytb ls2
	*Plasmodium juxtanucleare*	NC_008279.1	L	ls1 ss4 ss6 ls7 ls6 ss3 ls3 ls9 ss2 ls4 ls5 *cox3 ls8 ss5 ss1 cox1 cytb ls2
	*Plasmodium knowlesi*	NC_007232.1	C	ls1 ss6 ls7 ss3 ls3 ls9 ss2 ls4 *cox3 ls8 ss5 ss1 cox1 cytb
	*Plasmodium mexicanum*	NC_009960.2	L	ss3 ls3 ls9 ss2 *cox3 ls8 ss5 ss1 cox1 cytb ls1 ss4 ss6 ls7
	*Plasmodium reichenowi*	NC_002235.1	L	ss3 ls3 ls9 ss2 *cox3 ls8 ss5 ss1 cox1 cytb ls1 ss4 ss6 ls7
	*Plasmodium relictum*	NC_012426.1	C	ss3 ls3 ls9 ss2 ls4 *cox3 ls8 ss5 ss1 cox1 cytb ls1 ss4 ss6 ls7
	*Plasmodium simium*	NC_007233.1	C	ls1 ss6 ls7 ss3 ls3 ls9 ss2 ls4 *cox3 cox1 cytb ls8 ss5 ss1
	*Plasmodium vivax*	NC_007243.1	C	ls1 ss6 ls7 ss3 ls3 ls9 ss2 ls4 *cox3 ls8 ss5 ss1 cox1 cytb
Piroplasmida	*Babesia bovis*	NC_009902.1	L	cox1 *cox3 ls1 *ls2 *ls3 *cytb *ls4 ls5
	*Theileria parva*	NC_011005.1	L	cox1 *cox3 ls1 *ls3 *cytb *ls5 ls4
	*Theileria annulata*	CR940346.1	L	cox1 *cox3 ls1 *ls3 *ls2 *cytb *ls5 ls4

Circular and linear chromosomes are marked by C and L, respectively. Everywhere in the list of genes asterisk indicates the complementary strand relative to that specified in GenBank. The rightmost column shows the gene order using standard gene names

The analyzed 66 species with rhodophytic plastids are listed in Table 2. In the analysis of the evolution of *plastid chromosomal structure*, the genes available in many species and encoding proteins with a certain function were used: chaperone *clpC*; subunits of photosystem I *psaA, psaB, psaC, psaD, psaE, psaF, psaI, psaJ, psaK, psaL,* and *psaM*; subunits of photosystem II *psb28, psb30, psbA, psbB, psbC, psbD, psbE, psbF, psbH, psbI, psbJ, psbK, psbL, psbN, psbT, psbV, psbX, psbY,* and *psbZ*; rubisco large subunit *rbcL*; RNA polymerase subunits *rpoA, rpoB, rpoC1, rpoC2,* and *rpoZ*; ribosomal proteins *rpl1, rpl2, rpl3,rpl4, rpl5, rpl6, rpl9, rpl11, rpl12, rpl13, rpl14, rpl16, rpl18, rpl19, rpl20, rpl21, rpl22, rpl23, rpl24, rpl27, rpl28, rpl29, rpl31, rpl32, rpl33, rpl34, rpl35, rpl36, rps1, rps2, rps20, rps3, rps4, rps5, rps6, rps7, rps8, rps9, rps10, rps11, rps12, rps13, rps14, rps16, rps17, rps18,* and *rps19*; and elongation factor *tufA*. Paralogs of the *psbY* gene can be found in *Odontella sinensis, Phaeodactylum tricornutum, Thalassiosira pseudonana, Thalassiosira oceanica, Ulnaria acus, Asterionella formosa, Asterionellopsis glacialis, Didymosphenia geminata, Lithodesmium undulatum, Eunotia naegelii, Chaetoceros simplex, Roundia cardiophora, Cerataulina daemon,* and *Thalassiosira weissflogii*. Paralogs of the *clpC* gene can be found in *Theileria parva, Babesia bovis, Chromera velia, Thalassiosira oceanica, Nannochloropsis gaditana, Nannochloropsis granulata, Nannochloropsis oculata, Nannochloropsis salina, Nannochloropsis limnetica, Nannochloropsis oceanica,* and *Rhizosolenia imbricata*. Consecutive paralogs of the *rpoC2* can be found in *Theileria parva, Leucocytozoon caulleryi,* and *Plasmodium chabaudi*. In *Rhizosolenia imbricata*, a long duplication includes the *psbA, psaC, rps6, clpC, rps10, rps7,* and *rps12* genes. Notice formal errors in gene names in GenBank annotations: *rpo* instead of *proС1* in *Nannochloropsis gaditana*, *rpoC* instead of *proС1* in *Cyanidioschyzon merolae*, and *rpoС2-n-terminal* instead of *proС2* in *Babesia bovis*.

**Table 2:** Analyzed 66 species with rhodophytic plastids

Locus in GenBank	Species	#prot	#clust	#sing
NC_024079.1	*Asterionella formosa*	134	129	0
NC_024080.1	*Asterionellopsis glacialis*	145	138	1
NC_012898.1	*Aureococcus anophagefferens*	105	105	0
NC_012903.1	*Aureoumbra lagunensis*	110	110	0
NC_011395.1	*Babesia bovis T2Bo*	32	22	7
NC_021075.1	*Calliarthron tuberculosum*	201	200	1
NC_025313.1	*Cerataulina daemon*	132	130	0
NC_025310.1	*Chaetoceros simplex*	131	128	0
NC_020795.1	*Chondrus crispus*	204	204	0
NC_026522.1	*Choreocolax polysiphoniae*	71	71	0
NC_014340.2	*Chromera velia*	78	51	24
NC_014345.1	*Chromerida sp. RM11*	81	69	5
NC_024081.1	*Coscinodiscus radiatus*	139	130	0
NC_013703.1	*Cryptomonas paramecium*	82	79	3
NC_004799.1	*Cyanidioschyzon merolae strain 10D*	207	189	18
NC_001840.1	*Cyanidium caldarium*	197	186	11
NC_024082.1	*Cylindrotheca closterium*	161	141	13
NC_024083.1	*Didymosphenia geminata*	130	128	0
NC_014287.1	*Durinskia baltica*	129	127	0
NC_013498.1	*Ectocarpus siliculosus*	148	143	1
NC_004823.1	*Eimeria tenella strain Penn State*	28	21	7
NC_007288.1	*Emiliania huxleyi*	119	112	7
NC_024928.1	*Eunotia naegelii*	160	136	2
NC_015403.1	*Fistulifera solaris*	135	130	1
NC_016735.1	*Fucus vesiculosus*	139	139	0
NC_024665.1	*Galdieria sulphuraria*	182	181	1
NC_023785.1	*Gracilaria salicornia*	202	200	2
NC_006137.1	*Gracilaria tenuistipitata var. liui*	203	201	2
NC_021618.1	*Grateloupia taiwanensis*	233	201	32
NC_000926.1	*Guillardia theta*	147	142	5
NC_010772.1	*Heterosigma akashiwo*	156	139	3
NC_014267.1	*Kryptoperidinium foliaceum*	139	132	6
NC_027093.1	*Lepidodinium chlorophorum*	62	52	7
NC_024084.1	*Leptocylindrus danicus*	132	130	0
NC_022667.1	*Leucocytozoon caulleryi*	30	30	0
NC_024085.1	*Lithodesmium undulatum*	138	129	0
NC_020014.1	*Nannochloropsis gaditana*	119	116	3
NC_022259.1	*Nannochloropsis granulata*	125	123	0
NC_022262.1	*Nannochloropsis limnetica*	124	123	0
NC_022263.1	*Nannochloropsis oceanica*	126	123	1
NC_022260.1	*Nannochloropsis oculata*	126	123	0
NC_022261.1	*Nannochloropsis salina*	123	123	0
NC_001713.1	*Odontella sinensis*	140	128	9
NC_020371.1	*Pavlova lutheri*	111	102	9
NC_016703.2	*Phaeocystis antarctica*	108	108	0
NC_021637.1	*Phaeocystis globosa*	108	108	0
NC_008588.1	*Phaeodactylum tricornutum*	132	130	0
NC_023293.1	*Plasmodium chabaudi chabaudi*	31	31	0
NC_000925.1	*Porphyra purpurea*	209	209	0
NC_023133.1	*Porphyridium purpureum*	224	183	40
NC_021189.1	*Pyropia haitanensis*	211	210	1
NC_024050.1	*Pyropia perforata*	209	207	2
NC_007932.1	*Pyropia yezoensis*	209	206	3
NC_025311.1	*Rhizosolenia imbricata*	135	123	1
NC_009573.1	*Rhodomonas salina*	146	143	3
NC_025312.1	*Roundia cardiophora*	140	126	0
NC_018523.1	*Saccharina japonica*	139	139	0
NC_014808.1	*Thalassiosira oceanica CCMP1005*	142	126	1
NC_008589.1	*Thalassiosira pseudonana*	141	127	0
NC_025314.1	*Thalassiosira weissflogii*	141	127	0
NC_007758.1	*Theileria parva strain Muguga*	44	27	12
NC_001799.1	*Toxoplasma gondii RH*	26	21	5
NC_026851.1	*Trachydiscus minutus*	137	124	8
NC_016731.1	*Ulnaria acus*	130	128	0
NC_011600.1	*Vaucheria litorea*	139	138	1
NC_026523.1	*Vertebrata lanosa*	192	191	1

#Prot, number of plastid-encoded proteins in the species; #clust, number of clusters containing at least one from the species and one out of the species; and #sing, number of plastid-encoded proteins from the species not included in any cluster

### Evolution and reconstruction of mitochondrial structures in sporozoans

The reconstructions of chromosome structures of mitochondria in sporozoan class Aconoidasida were generated using the biological distance from data shown in Table 1.

**Fig. 4:**
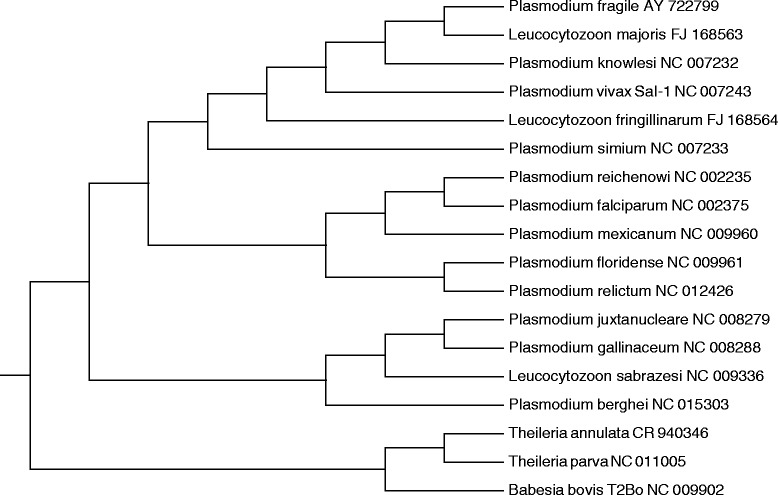
The tree of chromosome structures of mitochondria in sporozoan class Aconoidasida generated by the descent algorithm. (http://purl.org/phylo/treebase/phylows/study/TB2:S18685?x-access-code=bf7e98f7d030be83c7c2d1116c7faf0e&format=html)

The tree shown in Fig. 4 was generated by the descent algorithm. It consists of two clades including mitochondria of piroplasmids (genera *Babesia* and *Theileria*) and haemosporids (genera *Plasmodium* and *Leucocytozoon*), respectively. Two genera *Plasmodium* and *Leucocytozoon* cannot be resolved on the tree, in particular, due to the presence of linear and circular mitochondrial DNA in both of them.

The phylogenetic reconstruction generated by the descent algorithm of mitochondrial chromosome structures in Aconoidasida is shown in Table 3.

**Table 3:** Phylogenetic reconstruction of mitochondrial chromosome structures in sporozoan class Aconoidasida

Tree node	Chromosome structure
*Plasmodium fragile – Babesia bovis*	ss1 cox1 *cox3 ls1 *ls3 *ss3 *ls6 ls8 ss5 (C) | *ls7 *ss6 *ss4 ls9 ss2 ls4 ls5 cytb ls2 (L)
*Theileria annulata – Babesia bovis*	*ls4 ls5 cytb ls2 ls3 *ls1 cox3 *cox1 (L)
*Theileria annulata – Theileria parva*	*ls4 ls5 cytb ls2 ls3 *ls1 cox3 *cox1 (L)
*Theileria annulata* (*l*)	cox1 *cox3 ls1 *ls3 *cytb *ls5 ls4 (L)
*Theileria parva* (*l*)	cox1 *cox3 ls1 *ls3 *ls2 *cytb *ls5 ls4 (L)
*Babesia bovis* (*l*)	cox1 *cox3 ls1 *ls2 *ls3 *cytb *ls4 ls5 (L)
*Plasmodium fragile – Plasmodium berghei*	*ls7 *ss6 *ss4 *ls1 ls6 ss3 ls3 ls9 ss2 ls4 ls5 *cox3 ls8 ss5 ss1 cox1 cytb ls2 (L)
*Plasmodium juxtanucleare – Plasmodium berghei*	ls1 ss4 ss6 ls7 ls6 ss3 ls3 ls9 ss2 ls4 ls5 *cox3 ls8 ss5 ss1 cox1 cytb ls2 (L)
*Plasmodium juxtanucleare – Leucocytozoon sabrazesi*	ls1 ss4 ss6 ls7 ls6 ss3 ls3 ls9 ss2 ls4 ls5 *cox3 ls8 ss5 ss1 cox1 cytb ls2 (L)
*Plasmodium juxtanucleare – Plasmodium gallinaceum*	ls1 ss4 ss6 ls7 ls6 ss3 ls3 ls9 ss2 ls4 ls5 *cox3 ls8 ss5 ss1 cox1 cytb ls2 (L)
*Plasmodium juxtanucleare* (*l*)	ls1 ss4 ss6 ls7 ls6 ss3 ls3 ls9 ss2 ls4 ls5 *cox3 ls8 ss5 ss1 cox1 cytb ls2 (L)
*Plasmodium gallinaceum* (*l*)	ls1 ss4 ss6 ls7 ls6 ss3 ls3 ls9 ss2 ls4 ls5 *cox3 ls8 ss5 ss1 cox1 cytb ls2 (L)
*Leucocytozoon sabrazesi* (*l*)	ls1 ss4 ss6 ls7 ls6 ss3 ls3 ls9 ss2 ls4 ls5 *cox3 ls8 ss5 ss1 cox1 cytb ls2 (L)
*Plasmodium berghei* (*l*)	ls1 ss4 ss6 ls7 ls6 ss3 ls3 ls9 ss2 ls4 ls5 *cox3 ls8 ss5 ss1 cox1 cytb ls2 (L)
*Plasmodium fragile – Plasmodium relictum*	ss3 ls3 ls9 ss2 ls4 *cox3 ls8 ss5 ss1 cox1 cytb ls1 ss4 ss6 ls7 (L)
*Plasmodium reichenowi – Plasmodium relictum*	ss3 ls3 ls9 ss2 ls4 *cox3 ls8 ss5 ss1 cox1 cytb ls1 ss4 ss6 ls7 (L)
*Plasmodium floridense – Plasmodium relictum*	ss3 ls3 ls9 ss2 ls4 *cox3 ls8 ss5 ss1 cox1 cytb ls1 ss4 ss6 ls7 (L)
*Plasmodium floridense* (*l*)	ss3 ls3 ls9 ss2 ls4 *cox3 ls8 ss5 ss1 cox1 cytb ls1 ss4 ss6 ls7 (L)
*Plasmodium relictum* (*l*)	ss3 ls3 ls9 ss2 ls4 *cox3 ls8 ss5 ss1 cox1 cytb ls1 ss4 ss6 ls7 (C)
*Plasmodium reichenowi – Plasmodium mexicanum*	ss3 ls3 ls9 ss2 *cox3 ls8 ss5 ss1 cox1 cytb ls1 ss4 ss6 ls7 (L)
*Plasmodium reichenowi – Plasmodium falciparum*	ss3 ls3 ls9 ss2 *cox3 ls8 ss5 ss1 cox1 cytb ls1 ss4 ss6 ls7 (L)
*Plasmodium reichenowi* (*l*)	ss3 ls3 ls9 ss2 *cox3 ls8 ss5 ss1 cox1 cytb ls1 ss4 ss6 ls7 (L)
*Plasmodium falciparum* (*l*)	ss3 ls3 ls9 ss2 *cox3 ls8 ss5 ss1 cox1 cytb ls1 ss4 ss6 ls7 (L)
*Plasmodium mexicanum* (*l*)	ss3 ls3 ls9 ss2 *cox3 ls8 ss5 ss1 cox1 cytb ls1 ss4 ss6 ls7 (L)
*Plasmodium fragile – Plasmodium simium*	ss1 cox1 cytb ls1 ss4 ss6 ls7 ss3 ls3 ls9 ss2 ls4 *cox3 ls8 ss5 (C)
*Plasmodium fragile – Leucocytozoon fringillinarum*	ss1 cox1 cytb ls1 ss4 ss6 ls7 ss3 ls3 ls9 ss2 ls4 *cox3 ls8 ss5 (C)
*Plasmodium fragile – Plasmodium vivax*	ss1 cox1 cytb ls1 ss6 ls7 ss3 ls3 ls9 ss2 ls4 *cox3 ls8 ss5 (C)
*Plasmodium fragile – Plasmodium knowlesi*	ss1 cox1 cytb ls1 ss6 ls7 ss3 ls3 ls9 ss2 ls4 *cox3 ls8 ss5 (C)
*Plasmodium fragile – Leucocytozoon majoris*	ss1 cox1 cytb ls1 ss6 ls7 ss3 ls3 ls9 ss2 ls4 *cox3 ls8 ss5 (C)
*Plasmodium fragile* (*l*)	ls1 ss6 ls7 ss3 ls3 ls9 ss2 ls4 *cox3 ls8 ss5 ss1 cox1 cytb (C)
*Leucocytozoon majoris* (*l*)	ss3 ls3 ls9 ss2 ls4 *cox3 ls8 ss5 ss1 cox1 cytb ls1 ss6 ls7 (C)
*Plasmodium knowlesi* (*l*)	ls1 ss6 ls7 ss3 ls3 ls9 ss2 ls4 *cox3 ls8 ss5 ss1 cox1 cytb (C)
*Plasmodium vivax* (*l*)	ls1 ss6 ls7 ss3 ls3 ls9 ss2 ls4 *cox3 ls8 ss5 ss1 cox1 cytb (C)
*Leucocytozoon fringillinarum* (*l*)	ss3 ls3 ls9 ss2 ls4 *cox3 ls8 ss5 ss1 cox1 cytb ls1 ss6 ls7 ss4 (C)
*Plasmodium simium* (*l*)	ls1 ss6 ls7 ss3 ls3 ls9 ss2 ls4 *cox3 cox1 cytb ls8 ss5 ss1 (C)

Reconstruction was generated by the descent algorithm for the tree in Fig. 4. Circular and linear chromosomes are marked by C and L, respectively. The left column shows a non-leaf tree node by the first and the last leaves. The right column shows the chromosome structure in the node (the order of rows corresponds to the traversal of the tree in Fig. 4). The leaves are labelled by (*l*), only their chromosomal structures are feeded to the input of our algorithm

**Fig. 5:**
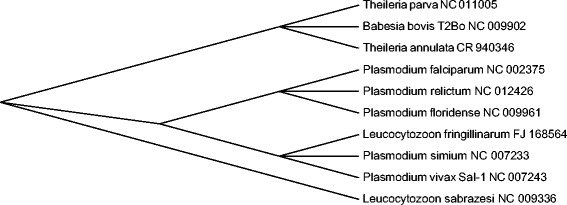
The tree of chromosome structures of mitochondria in sporozoan class Aconoidasida. The tree was generated by the algorithm from the Section “The first algorithm solving the reconstruction problem for structures without paralogs”. (http://purl.org/phylo/treebase/phylows/study/TB2:S18685?x-access-code=bf7e98f7d030be83c7c2d1116c7faf0e&format=html)

For mitochondria, the algorithm from the Section “The first algorithm solving the reconstruction problem for structures without paralogs” generated a valid unrooted non-binary tree shown in Fig. 5. The leaves with the same structures generated by the same algorithm were combined into a single leaf. Namely, the leaves of ***Plasmodium vivax***, *Leucocytozoon majoris*, *Plasmodium fragile*, and *Plasmodium knowlesi* are represented by the former (marked by the bold font); the same applies to the leaves of ***Plasmodium falciparum***, *Plasmodium reichenowi*, and *Plasmodium mexicanum* as well as of ***Leucocytozoon sabrazesi***, *Plasmodium juxtanucleare*, *Plasmodium gallinaceum*, and *Plasmodium berghei*. The tree was rooted at the node to provide the best tree as the total one-way distance along all directions from the root to edges. The proper reconstruction generated by the same algorithm is shown in Table 4. Thus, both algorithms reconstructed identical evolutionary scenario of chromosome structures.

**Table 4:** Phylogenetic reconstruction of mitochondrial chromosome structures in sporozoan class Aconoidasida

Tree node	Chromosome structure
*Theileria parva* –*Leucocytozoon sabrazesi*	ss1 cox1 *cox3 ls1 *ls3 *ss3 *ls6 ls8 ss5 (C) | *ls7 *ss6 *ss4 ls9 ss2 ls4 ls5 cytb ls2 (L)
*Theileria parva–Theileria annulata*	*ls4 ls5 cytb ls2 ls3 *ls1 cox3 *cox1 (L)
*Theileria parva* (*l*)	cox1 *cox3 ls1 *ls3 *ls2 *cytb *ls5 ls4 (L)
*Babesia bovis* (*l*)	cox1 *cox3 ls1 *ls2 *ls3 *cytb *ls4 ls5 (L)
*Theileria annulata* (*l*)	cox1 *cox3 ls1 *ls3 *cytb *ls5 ls4 (L)
*Leucocytozoon sabrazesi* (*l*)	ls1 ss4 ss6 ls7 ls6 ss3 ls3 ls9 ss2 ls4 ls5 *cox3 ls8 ss5 ss1 cox1 cytb ls2 (L)
*Plasmodium falciparum–Plasmodium vivax*	ss3 ls3 ls9 ss2 ls4 *cox3 ls8 ss5 ss1 cox1 cytb ls1 ss4 ss6 ls7 (L)
*Plasmodium falciparum–Plasmodium floridense*	ss3 ls3 ls9 ss2 ls4 *cox3 ls8 ss5 ss1 cox1 cytb ls1 ss4 ss6 ls7 (L)
*Plasmodium falciparum* (*l*)	ss3 ls3 ls9 ss2 *cox3 ls8 ss5 ss1 cox1 cytb ls1 ss4 ss6 ls7 (L)
*Plasmodium relictum* (*l*)	ss3 ls3 ls9 ss2 ls4 *cox3 ls8 ss5 ss1 cox1 cytb ls1 ss4 ss6 ls7 (C)
*Plasmodium floridense* (*l*)	ss3 ls3 ls9 ss2 ls4 *cox3 ls8 ss5 ss1 cox1 cytb ls1 ss4 ss6 ls7 (L)
*Leucocytozoon fringillinarum–Plasmodium vivax*	ss1 cox1 cytb ls1 ss4 ss6 ls7 ss3 ls3 ls9 ss2 ls4 *cox3 ls8 ss5 (C)
*Leucocytozoon fringillinarum* (*l*)	ss3 ls3 ls9 ss2 ls4 *cox3 ls8 ss5 ss1 cox1 cytb ls1 ss6 ls7 ss4 (C)
*Plasmodium simium* (*l*)	ls1 ss6 ls7 ss3 ls3 ls9 ss2 ls4 *cox3 cox1 cytb ls8 ss5 ss1 (C)
*Plasmodium vivax* (*l*)	ls1 ss6 ls7 ss3 ls3 ls9 ss2 ls4 *cox3 ls8 ss5 ss1 cox1 cytb (C)

Reconstruction was generated by the algorithm from the Section “The first algorithm solving the reconstruction problem for structures without paralogs” for the tree in Fig. 5

### Evolution of chromosome structures in plastids of rhodophytic branch

The tree (not shown) generated by the algorithm from the Sections “Calculation of the breakpoint and biological distances for structures with paralogs” and “Calculation of biological distance with paths present” for the data shown in Table 2 is not reasonable. This can be attributed to the twofold difference between the weights of trees reconstructed by this algorithm and the minimum tree.

**Fig. 6:**
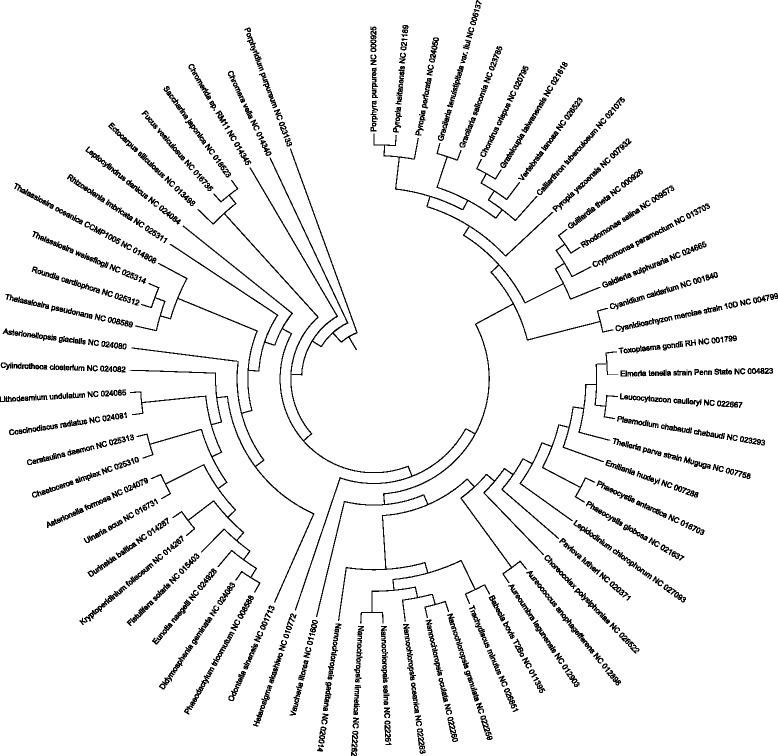
Tree of chromosomal structures of rhodophytic plastids generated by the descent algorithm. The data were obtained from GenBank for chromosomes listed in Table 2. The chromosome structures that were fed to our algorithm are shown in Additional file 1, #3, Tables S3*a–*S3*b* in rows denoted by (*l*). (http://purl.org/phylo/treebase/phylows/study/TB2:S18685?x-access-code=bf7e98f7d030be83c7c2d1116c7faf0e&format=html)

The tree of plastids generated by the descent algorithm is shown in Fig. 6, and it seems quite possible. We discuss it below.

This tree is in good agreement with previously published data, in particular, with the corresponding trees of species. The most significant distinctions are special tree positions of photosynthetic alveolate *Chromera velia* and rhodophytic alga *Porphyridium purpureum*, whose order of genes substantially differs from that in related species. This has been mentioned previously in the study of the *moeB* gene regulation [29]. A separate clade was formed by plastids of the *Nannochloropsis* genus, which constitute an isolated portion of the large Stramenopiles phylum [30].

All diatoms composed a large clade also including certain stramenopiles as well as alveolate species *Durinskia baltica* and *Kryptoperidinium foliaceum*, whose plastids are of tertiary origin descending from diatom ones [31].

Another large clade was formed by plastids of rhodophytic algae excluding *Porphyridium purpureum*, cryptophytes, certain alveolates, haptophytes, and stramenopiles *Aureococcus anophagefferens* and *Aureoumbra lagunensis* [32] as well as raphidophyte *Heterosigma akashiwo* [33] and xanthophyte *Vaucheria litorea*.

All brown algae *Ectocarpus siliculosus*, *Fucus vesiculosus*, and *Saccharina japonica* [34, 35] composed another clade.

The alveolate species whose plastids are close to those of rhodophytic algae include all considered sporozoans as well as the photosynthetic alveolate *Chromerida* sp. RM11. The common origin of these plastids has been previously confirmed by protein alignment [36, 37]. In addition, we have predicted a uniform *ycf24* (*sufB*) expression regulation in plastids of sporozoans and certain rhodophytic algae [38], which corroborates the close positions of these species on the generated tree. A significant variation between plastids is observed among stramenopiles. The distinction of haptophytes agrees with the independent origin of plastids in Haptophyta and Stramenopiles proposed previously [39]. However, the independent origin of cryptophyte plastids is not confirmed. Overall, one can propose that plastids of rhodophytic branch are monophyletic and descend from plastids of rhodophytic algae, while this statement seems questionable for cryptophytes and sporozoans.

**Fig. 7:**
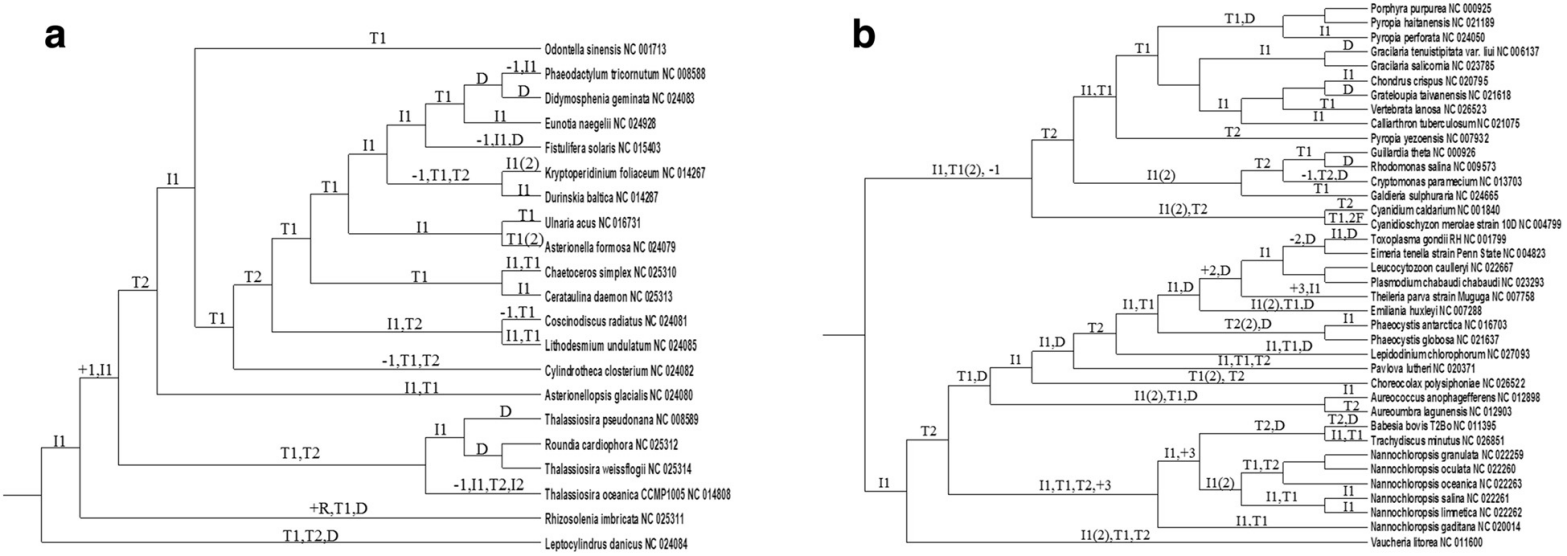
**a** Evolutionary scenario of chromosome structures along the small tree. The following events are shown on edges: −1, loss of one of two paralogs of gene *psbY*; +1, emergence of a paralog of gene *psbY*; +R, emergence of an inverted repeat of a chromosome segment; I1, inversion of a chromosome segment; T1, transversion of a chromosome segment; T2, translocation of a chromosome segment; I2, insertion of a chromosome segment, and D – disappearance of a chromosome segment. The number of the events is given in parentheses when greater than 1. For the reconstruction details, see Table S3*a* in Additional file 1, #3. **b** Evolutionary scenario of chromosome structures along the large tree. The following events are shown on edges: −1, loss of gene *psbY*; −2, loss of one of two paralogs of gene *rpoC2*; +2, emergence of a paralog of gene *rpoC2*; +3, emergence of a paralog of gene *clpC*; I1, inversion of a chromosome segment; T1, transversion of a chromosome segment; T2, translocation of a chromosome segment; 2 F, fusion of two paralogs of gene *rpoC2* into one large gene, and D, deletion of a chromosome segment. The number of the events is given in parentheses when greater than 1. For the reconstruction details, see Table S3b in Additional file 1, #3

The tree of chromosome structures in apicoplasts was reconstructed using an entirely different approach in [40], Fig. 7. It shows some similarity with the corresponding subtree of our tree of plastids. For instance, Chromerida is an early separated branch in both cases. According to the number of edges on the path, Plasmodium neighbors Toxoplasma and the both neighbor Theileria. At the same time, the trees are distinct, which can be attributed to a different number of analyzed genes and species (there are many distant species in our case).

### Reconstruction of chromosome structures in rhodophytic plastids along the tree of their evolution by the descent algorithm

For brevity, the reconstructions in two subtrees of the tree shown in Fig. 6 are presented: from the common ancestor of *Leptocylindrus danicus* and *Odontella sinensis* (hereafter, *small* tree) and from the common ancestor of *Porphyra purpurea* and *Vaucheria litorea* NC 011600 (hereafter, *large* tree). The result of reconstruction for the small tree is given in Additional file 1, #3 (Table S3*a*) and the corresponding evolutionary scenario is shown in Fig. 7*a*. The corresponding reconstruction for the large subtree is shown in Table S3*b* and Fig. 7*b*.

In Tables S3*a*–S3*b*, the genes are specified according to their order on the chromosome; everywhere asterisk marks genes on the complementary strand. Both trees were reconstructed using a Boolean linear programming with 2 millions of variable and 4 millions of linear equalities and inequalities. Two paralogs of the *рsbY*, *rpoC2*, *clpC*, and some other genes can be distinguished by the subscript index. If a structure contains several chromosomes, they are separated by vertical lines.

Note that all plastid chromosomes are circular in the reconstruction.

The result of reconstruction for the large tree is shown in Additional file 1, #3 (Table S3*b*) and the corresponding evolutionary scenario is shown at Fig. 7*b*. As previously, all chromosomes are circular; the most of ancestral structures include a single chromosome in the subtree up to the common ancestor of *Porphyra purpurea* and *Galdieria sulphuraria*; while most structures contain several chromosomes in the remaining part of the subtree. This can point to active chromosome rearrangements in ancestral species in this part of the subtree.

The presence of several circular chromosomes in a structure does not necessarily indicate the presence of an ancestral species with such structure, since it can correspond to evolutionary periods when translocations of chromosome regions occurred. Indeed, translocation is represented by two consecutive operations: cutting out and circularization of a fragment and its insertion into a different locus. Thus, reconstruction can expose intermediate states of such rearrangements. The generated scenario includes only a small number of such events.

## Conclusions

A high-level model of chromosome structure was proposed together with computer programs that allow its efficient utilization. A database of protein families encoded in rhodophytic plasmids was generated (available at http://lab6.iitp.ru/ppc/redline67/). The scenarios of chromosome rearrangements were deduced in rhodophytic plastids and sporozoan mitochondria. The scenarios, in particular, demonstrate the similarity of chromosome structures in sporozoan apicoplasts and rhodophytic plastids, which agrees with the previously proposed common origin of expression regulation in a few genes from these species, including the common pattern of translation initiation regulation for genes coding for DNA*-*directed RNA polymerase beta chain and the protein SufB involved in iron-sulfur cluster formation. The similarity of chromosome structures is observed in rhodophytic and cryptophytic plastids. On the other hand, our results indicate an early and independent segregation of diatom and haptophyte plastids.

Chromosome structures in plastids of the rhodophyte alga *Porphyridium purpureum* and the photosynthetic alveolate alga *Chromera velia* deviate considerably from those in their relatives. In such cases, chromosomes cannot be used to infer phylogenetic relationships but can provide comparative information for understanding the role of chromosome rearrangement in gene expression regulation. Such analysis was published for plastids of higher plants [41].

Chromosome rearrangements can considerably affect patterns of gene expression, particularly due to competition between RNA polymerases [42]. In chromosomes with labile structures, transcription terminators are naturally expected to occur between genes, and gene expression is largely regulated at the translation level, which was described for many plastids e.g., [26].

The model and computer programs can be used to explore the evolution of chromosome structures in other genomes.

## Declarations

### Availability of data and materials

The database of plastid and mitochondrial protein clusters is available at http://lab6.iitp.ru/ppc/redline67/. The programs below were tested on several artificial sets of data, which are available together with program distributives.

1. Chromo program:
  Project name: Chromo
  Project home page: http://lab6.iitp.ru/en/chromoggl/
  Operating system: Windows 7
  Programming language: C++
  License: Freeware
2. ChromoReconstruction program:
  Project name: ChromoReconstruction
  Project home page: http://lab6.iitp.ru/en/chromoggl/
  Operating system: Windows 7
  Programming language: Delphi
  License: Freeware

## Additional file

Additional file 1:
**#1 (Standard and extra operations allowed to transform initial structures and the joint graph); #2 (Figurs to the algorithm of transforming a joint graph into the final form: the case of different operation weights and all operations.); #3 (Reconstruction of chromosome structures in plastids of rhodophytic branch along the tree of their evolution shown in Fig.** 6 **, Tables S3** ***a*** **and S3** ***b*** **.).** (DOCX 679 kb)

## References

[1] Gorbunov KY, Gershgorin RA, Lyubetsky VA (2015). Rearrangement and inference of chromosome structures. Mol Biol (Mosk).

[2] Ed K, Newman Alexandra M (2013). Practical guidelines for solving difficult linear programs. Surveys in Operations Research and Management Science.

[3] Ed K, Newman Alexandra M (2013). Practical guidelines for solving difficult mixed integer linear programs. Surveys in Operations Research and Management Science.

[4] Schrijver A (1986). Theory of linear and integer programming.

[5] Gorbunov KYu, Lyubetsky VA. Exact linear algorithms for structure rearrangement. Problems of InformationTtransmission. 2015. in press.

[6] Gorbunov KYu., Lyubetsky VA. Exact linear algorithms for the shortest rearrangement of structures with different operation weights. Problems of InformationTtransmission. 2015. in press.

[7] Braga MDV, Willing E, Stoye J (2011). Double cut and join with insertions and deletions. J Comput Biol.

[8] da Silva PH, Machado R, Dantas S, Braga MDV (2013). DCJ-indel and DCJ-substitution distances with distinct operation costs. Algorithms Mol Biol.

[9] Compeau PEC (2013). DCJ-indel sorting revisited. Algorithms Mol Biol.

[10] Compeau PEC (2014). A generalized cost model for DCJ-indel sorting. Lect Notes Comput Sci.

[11] Hilker R, Sickinger C, Pedersen C, Stoye J (2012). UniMoG - a unifying framework for genomic distance calculation and sorting based on DCJ. Bioinformatics.

[12] Rusin LY, Lyubetskaya EV, Gorbunov KY, Lyubetsky VA (2014). Reconciliation of gene and species trees. BioMed Res Int (Current Advances in Molecular Phylogenetics).

[13] Gorbunov KY, Laikova ON, Rodionov DA, Gelfand MS, Lyubetsky VA (2010). Evolution of regulatory motifs of bacterial transcription factors. In Silico Biol.

[14] Lopatovskaya KV, Gorbunov KY, Rusin LY, Seliverstov AV, Lyubetsky VA (2010). The evolution of proline synthesis transcriptional regulation in gammaproteobacteria. Mosc Univ Biol Sci Bull.

[15] Alon N, Chor B, Pardi F, Rapoport A (2010). Approximate maximum parsimony and ancestral maximum likelihood. IEEE/ACM Trans Comput Biol Bioinf.

[16] Blanchette M, Kunisawa T, Sankoff D (1999). Gene order breakpoint evidence in animal mitochondrial phylogeny. J Mol Evol.

[17] Chauve C, El-Mabrouk N, Tannier E. Models and Algorithms for Genome Evolution. 19 volume, Computational Biology, Springer; 2013. doi: 10.1007/978-1-4471-5298-9.

[18] Yancopoulos S, Attie O, Friedberg R (2005). Efficient sorting of genomic permutations by translocation, inversion and block interchange. Bioinformatics.

[19] Hannenhalli S, Pevzner PA. Transforming Men into Mice (Polynomial Algorithm for Genomic Distance Problem). In FOCS IEEE Computer Society; 1995:581–592. doi: 10.1109/SFCS.1995.492588.

[20] Bergeron A, Mixtacki J, Stoye J (2006). A unifying view of genome rearrangements. Algorithms in Bioinformatics, LNCS.

[21] Kou L, Markowsky G, Berman L (1981). A fast algorithm for Steiner trees. Acta Inform.

[22] Gershgorin RA, Gorbunov KY, Seliverstov AV, Lyubetsky VA (2015). Evolution of Chromosome Structures.

[23] Martinez FV, Feijão P, Braga MDV, Stoye J (2015). On the family-free DCJ distance and similarity. Algorithms Mol Biol.

[24] Zelikovsky A (1993). An 11/ 6-approximation algorithm for the network Steiner problem. Algorithmica.

[25] Cheng X, Du D-Z (2001). Steiner Trees in Industry.

[26] Zverkov OA, Seliverstov AV, Lyubetsky VA (2012). Plastid-encoded protein families specific for narrow taxonomic groups of algae and protozoa. Mol Biol.

[27] Lyubetsky VA, Seliverstov AV, Zverkov OA (2013). Elaboration of the homologous plastid-encoded protein families that separate paralogs in magnoliophytes. Mathematical Biology and Bioinformatics.

[28] Lyubetsky VA, Seliverstov AV, Zverkov OA (2013). Transcription regulation of plastid genes involved in sulfate transport in Viridiplantae. BioMed Res Int.

[29] Zverkov OA, Seliverstov AV, Lyubetsky VA (2015). A database of plastid protein families from red algae and Apicomplexa and expression regulation of the moeB gene. BioMed Res Int.

[30] Wei L, Xin Y, Wang D, Jing X, Zhou Q, Su X (2013). Nannochloropsis plastid and mitochondrial phylogenomes reveal organelle diversification mechanism and intragenus phylotyping strategy in microalgae. BMC Genomics.

[31] Imanian B, Pombert JF, Keeling PJ (2010). The complete plastid genomes of the two ‘dinotoms’ Durinskia baltica and Kryptoperidinium foliaceum. PLoS ONE.

[32] Ong HC, Wilhelm SW, Gobler CJ, Bullerjahn G, Jacobs MA, McKay J (2010). Analyses of the complete chloroplast genome sequences of two members of the Pelagophyceae: Aureococcus anophagefferens CCMP1984 and Aureoumbra lagunensis CCMP1507. J Phycol.

[33] Cattolico RA, Jacobs MA, Zhou Y, Chang J, Duplessis M, Lybrand T (2009). Chloroplast genome sequencing analysis of Heterosigma akashiwo CCMP452 (West Atlantic) and NIES293 (West Pacific) strains. BMC Genomics.

[34] Wang X, Shao Z, Fu W, Yao J, Hu Q, Duan D (2013). Chloroplast genome of one brown seaweed, Saccharina japonica (Laminariales, Phaeophyta): its structural features and phylogenetic analyses with other photosynthetic plastids. Mar Genomics.

[35] Le Corguille G, Pearson G, Valente M, Viegas C, Gschloessl B, Corre E (2009). Plastid genomes of two brown algae, Ectocarpus siliculosus and Fucus vesiculosus: further insights on the evolution of red-algal derived plastids. BMC Evol Biol.

[36] Janouškovec J, Horak A, Obornik M, Lukes J, Keeling PJ (2010). A common red algal origin of the apicomplexan, dinoflagellate, and heterokont plastids. Proc Natl Acad Sci U S A.

[37] Janouškovec J, Liu SL, Martone PT, Carre W, Leblanc C, Collen J (2013). Evolution of red algal plastid genomes: ancient architectures, introns, horizontal gene transfer, and taxonomic utility of plastid markers. PLoS ONE.

[38] Sadovskaya TA, Seliverstov AV (2009). Analysis of the 5′-leader regions of several plastid genes in protozoa of the phylum apicomplexa and red algae. Mol Biol.

[39] Baurain D, Brinkmann H, Petersen J, Rodriguez-Ezpeleta N, Stechmann A, Demoulin V (2010). Phylogenomic evidence for separate acquisition of plastids in cryptophytes, haptophytes, and stramenopiles. Mol Biol Evol.

[40] Garg A, Stein A, Zhao W, Dwivedi A, Frutos R, Cornillot E (2014). Sequence and annotation of the apicoplast genome of the human pathogen babesia microti. PLoS ONE.

[41] Andreica A, Chira C (2014). Best-order crossover in an evolutionary approach to multi-mode resource-constrained project scheduling. International Journal of Computer Information System and Industrial Management Applications.

[42] Andreica A, Chira C (2014). Best-order crossover for permutation-based evolutionary algorithms. Appl Intell.

