# Supplementary material for: Algorithms for reconstruction of chromosomal structures
Source: BMC Bioinformatics. 2016 Jan 19;17:40. doi: 10.1186/s12859-016-0878-z (PMC4717669; doi:10.1186/s12859-016-0878-z)
Supplement: Additional file 1: — #1 (Standard and extra operations allowed to transform initial structures and the joint graph); #2 (Figurs to the algorithm of transforming a joint graph into the final form: the case of different operation weights and all operations.); #3 (Reconstruction of chromosome structures in plastids of rhodophytic branch along the tree of their evolution shown in Fig. 6 , Tables S3 a and S3 b .). (DOCX 679 kb) [file 12859_2016_878_MOESM1_ESM.docx]

**Supplementary Materials to the paper Lyubetsky at el.**

**#1. Standard and extra operations allowed to transform a structure; corresponding operations for the joint graph of two structures.**

**Standard operations for a structure:**

**Standard operations for the joint graph:**


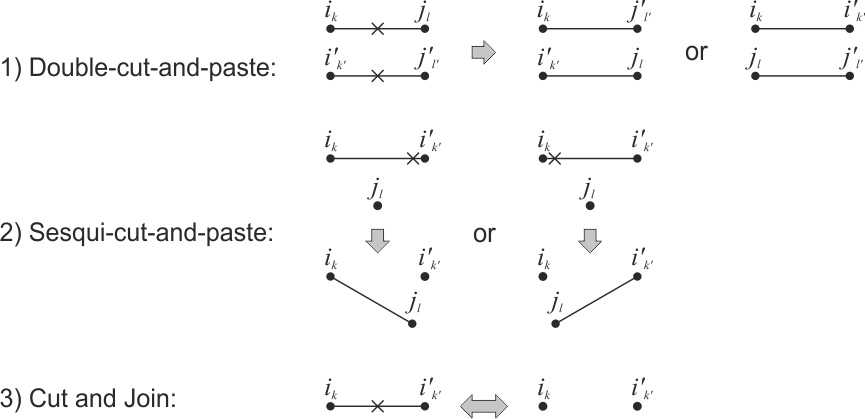


**Extra operations for a structure:**

**Extra operations for the joint graph:**

**
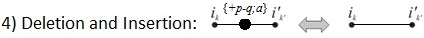
**

**#2. Figurs to the algorithm of transforming a joint graph into the final form. The case of different operation weights, the standard and extra operations of deletion and insertion.**

**Step 2.**


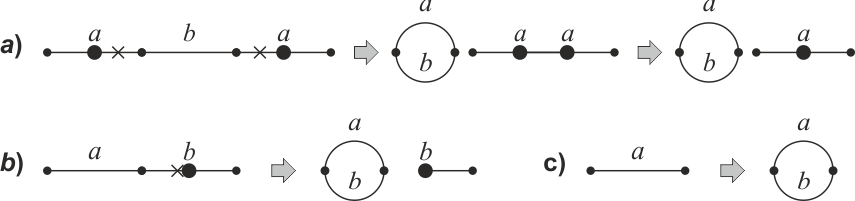


**Step 3.**

**3.1. 1*a*+1*b*=1*_c_*.**


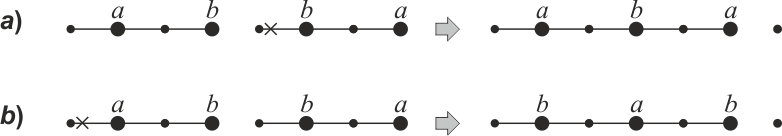


**3.2. 2*a*+3*b*=1*_b_*, 2*b*+3*a*=1*_a_*, 2*b'*+3*a*=1*_a_*, 2*b*+3*a'*=1*_a_*, and 2*b'*+3*a'*=1*'*.**


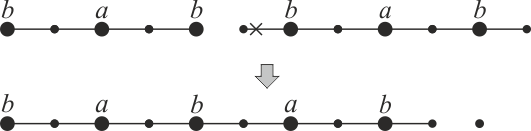


**3.3. 2+3=1*_c_*.**


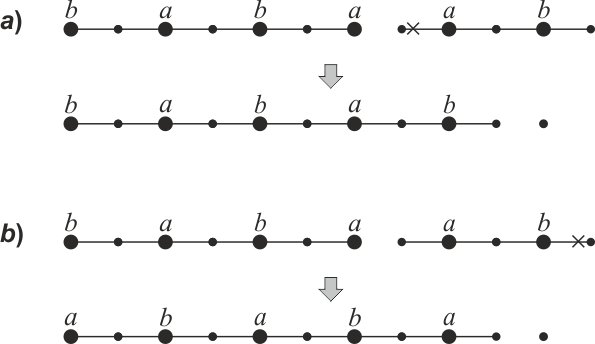


**3.6. 1*a*+2=2*a* and 1*b*+2=2b**.


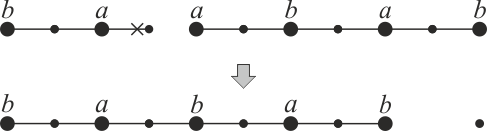


**3.7. 1*a*+3=3*a* and 1*b*+3=3*b*.**


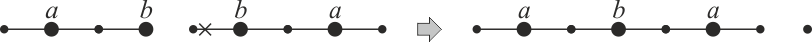


**3.11. 1*a*+1*a*=3*a* and 1*b*+1*b*=3*b*.**


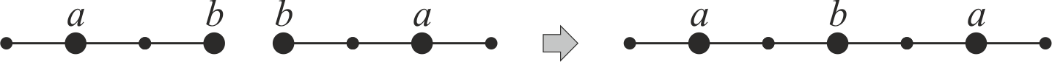


**3.12. 1*a*+2*b*=2, 1*a*+2*b'*=2, and 1*b*+2*a*=2.**


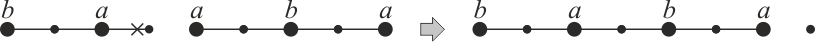


**3.13. 1*a*+3*b*=3, 1*b*+3*a*=3, and 1*b*+3*a'*=3.**


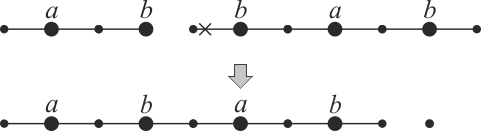


**Step 4.**

**4.1. “Loop” + (“circle” or “path *K* with a *b*-node”)** = **“circle” or “path of the same type as *K***,**”** correspondingly.


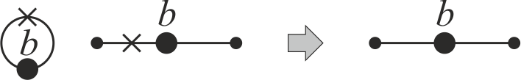


**4.2**. **“Circle” + (“circle” or “path *K* with *b*- and *a*-nodes)** = **“circle” or “path of the same type as *K.*”**


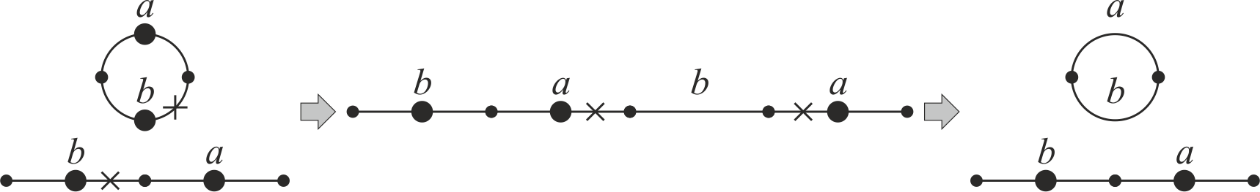


**4.3. 2*a*+2*b*=2+1*'*.**


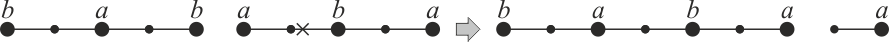


**4.4. 3*a*+3*b*=3.**


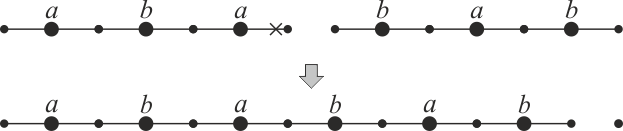


**4.5. 2*a*+3=1*a* and 2*b*+3=1*b***.


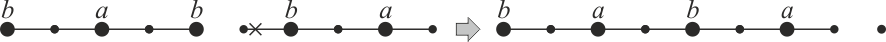


**4.6. 3*a*+2=1*a* and 3*b*+2=1*b*.**


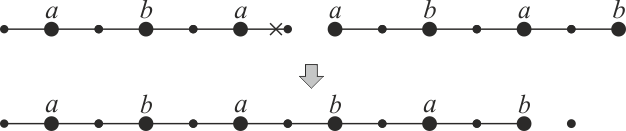


**4.7. 2*a*+2*a*=2*a* and 2*b*+2*b*=2*b*.**


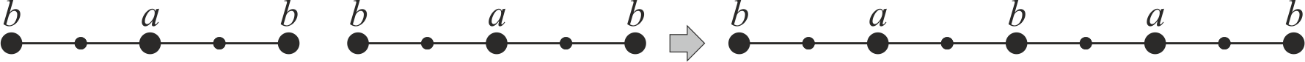


**4.8. 3*a*+3*a*=3*a* and 3*b*+3*b*=3*b*.**


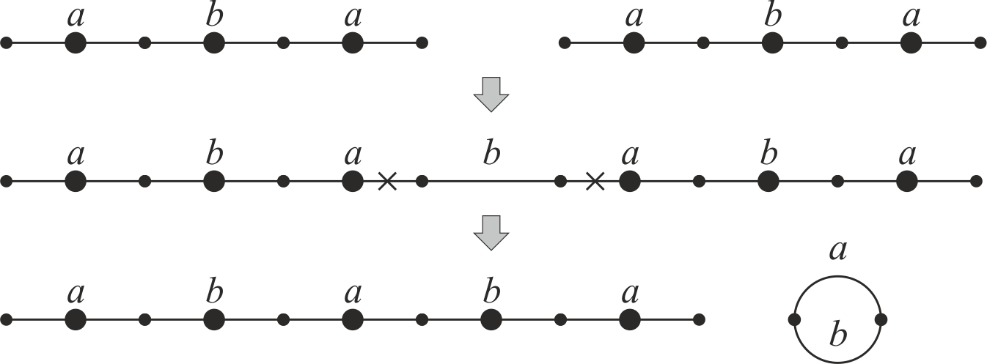


**4.9. 1*a*+2*a*=1*a* and 1*b*+2*b*=1*b*.**


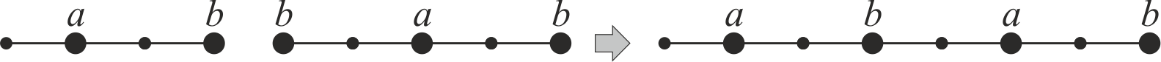


**4.10. 1*a*+3*a*=1*a* and 1*b*+3*b*=1*b*.**


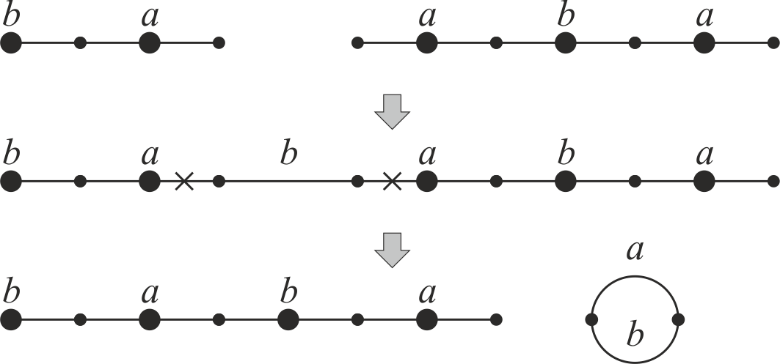


**4.11. 2*a*+2=2 and 2*b*+2=2.**


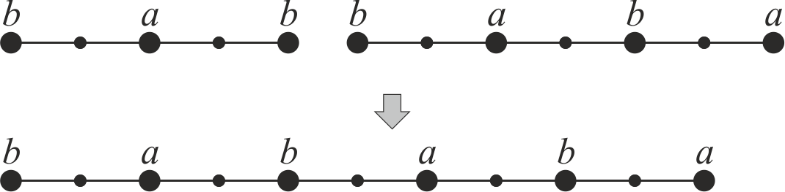


**4.12. 3*a*+3=3, 3*b*+3=3.**


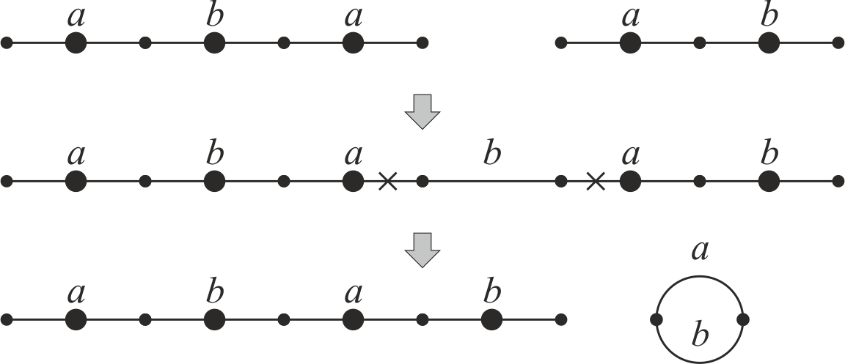


**4.13. 2+2=2+1'.**


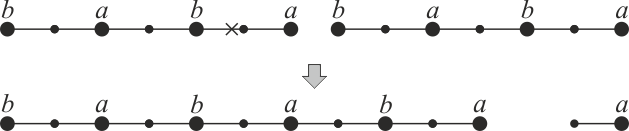


**4.14. 3+3=3.**


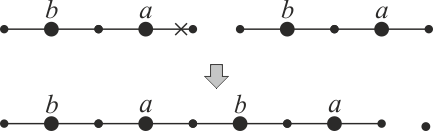


**4.15. 1*_a_*+1*_a_*=1*_a_*, 1*_b_*+1*_b_*=1*_b_*, and 1*_b_*+1*_c_*=1*_b_* (set *c*=*b*)**.


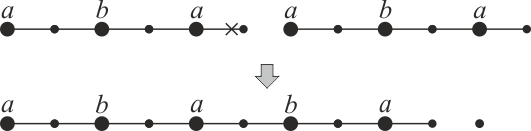


**4.16. 1*a*+1*_b_*=1*a*, 1*b*+1*_a_*=1*b*, and 1*a*+1*_c_*=1*a* (set *c*=*b*)**.


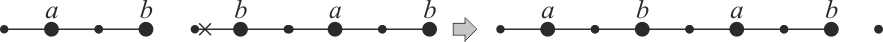


**4.17. 1*a*+1*_a_*=1*a*, 1*b*+1*_b_*=1*b*, and 1*b*+1*_с_*=1*b* (set *c*=*b*).**


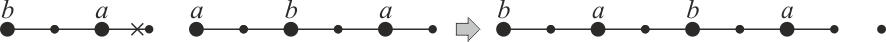


**4.18. 2*a*+1*_b_*=2*a*, 2*b*+1*_a_*=2*b*, and 2*a*+1*_c_*=2*a* (set *c*=*b*)**.


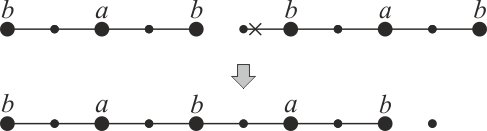


**4.19. 3*a*+1*_a_*=3*a*, 3*b*+1*_b_*=3*b*, and 3*b*+1*_c_*=3*b* (set *c*=*b*).**


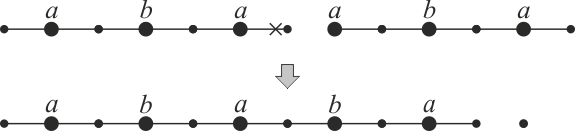


**4.20. 2+1*_a_*=2, 2+1*_b_*=2, and 2+1*_c_*=2 (set *c*=*b*).**


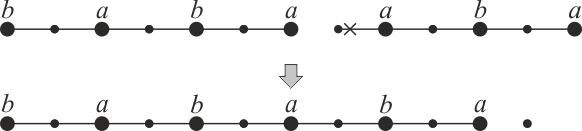


**4.21. 3+1*_a_*=3, 3+1*_b_*=3, and 3+1*_c_*=3 (set *c*=*b*).**


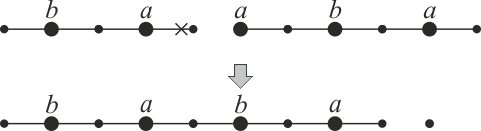


**4.24.**


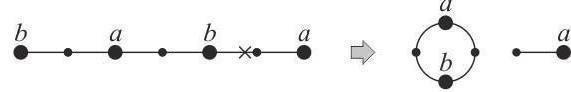


**Step 5.**


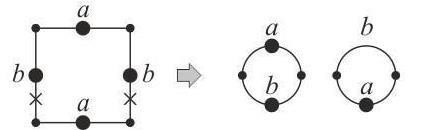


**#3. Reconstruction of chromosome structures in plastids of rhodophytic branch along the tree of their evolution.**

**Table S3*a***. **Reconstruction of chromosome structures in plastids of rhodophytic branch along the small tree.** The rows indicated by (*l*) correspond to the leaves of the tree and include the initial chromosome structures (whose accession numbers are given in Table 1); they are given as input to the algorithm. The left column specifies internal tree nodes by the first and the last leaves in the tree in Fig. 7*a*. The right column shows the resulting chromosome structure obtained for this node.

| *Leptocylindrus danicus – Odontella sinensis* | rbcl *psbz rpl35_1 rpl20_1 psam psad *rpl12 *rpl1 *rpl11 *rps14 psae_1 psbx psbv rpl19 *psab *psaa *psaj *psaf *psb1 *ycf39 *psal psbe psbf psbl psbj *psbc *psbd *rps2 *rpoc2_1 *rpoc1 *rpob *rps20 rpl33 rps18 ycf3 ycf33 *psa1 psbk *psbh psbn *psbt *psbb *ycf31 *psbh psbn *psbt *psbb *rpoc1 \| rpl32_1 rpl21_1 rpl27_1 rpl34_1 psba_1 psby_1 psac_1 rps6_1 *clpc_1 *rps10_1 *tufa *rps7 *rps12 *rpl31 *rps9 *rpl13 *rpoa *rps11 *rps13 *rpl36 *rps5 *rpl18 *rpl6 *rps8 *rpl5 *rpl24 *rpl14 *rps17 *rpl29 *rpl16 *rps3 *rpl22 *rps19 *rpl2 *rpl23 *rpl4 *rpl3 psb28_2 rps4_2 rps16_2 ycf35_2 *psba_2 *rpl34_2 *rpl27_2 *rpl21_2 *rpl32_2 *psby_2 *rpl20_2 *rpl35_2 *psae_2 *ycf35_1 *rps16_1 *rps4_1 *psb28_1 psac_2 psbw |
| --- | --- |
| *Leptocylindrus danicus* (*l*) | rbcl *psbz rpl35_1 rpl20_1 psam psad *rpl12 *rpl1 *rpl11 *rps14 psae_1 psbx psbv rpl19 *ycf3 *rps18 *rpl33 rps20 rpob rpoc1 rpoc2_1 rps2 *psbk psa1 psbd psbc psaa psab psbb psbt *psbn psbh ycf33 *psbj *psbl *psbf *psbe psal ycf39 psb1 psaf psaj psac rps6_1 *rpl34_1 *rpl27_1 *rpl21 rpl3 rpl4 rpl23 rpl2 rps19 rpl22 rps3 rpl16 rpl29 rps17 rpl14 rpl24 rpl5 rps8 rpl6 rpl18 rps5 rpl36 rps13 rps11 rpoa rpl13 rps9 rpl31 rps12 rps7 tufa rps10_1 clpc_1 *rps16_1 psb28_1 rps4_1 psby_1 rpl32_1 ycf35_1 psba_1 *psac_1 |
| *Rhizosolenia imbricata – Odontella sinensis* | *psab *psaa *psaj *psaf *psb1 *ycf39 *psal psbe psbf psbl psbj *psad *psbc *psbd *psbz *rpl12 *rpl1 *rpl11 psbx psbv rpl19 rps14 psam *rps2 *rpoc2_1 *rpoc1 *rpob *rps20 rpl33 rps18 ycf3 psae_1 ycf33 *psa1 psbk *psbh psbn *psbt *psbb *ycf31 *psbh psbn *psbt *psbb rbcl *rpoc1 \| rpl32_1 rpl21_1 rpl27_1 rpl34_1 psba_1 rpl35_1 rpl20_1 psby_1 psac_1 rps6_1 *clpc_1 *rps10_1 *tufa *rps7 *rps12 *rpl31 *rps9 *rpl13 *rpoa *rps11 *rps13 *rpl36 *rps5 *rpl18 *rpl6 *rps8 *rpl5 *rpl24 *rpl14 *rps17 *rpl29 *rpl16 *rps3 *rpl22 *rps19 *rpl2 *rpl23 *rpl4 *rpl3 psb28_2 rps4_2 rps16_2 ycf35_2 *psba_2 *rpl34_2 *rpl27_2 *rpl21_2 *rpl32_2 *psby_2 *rpl20_2 *rpl35_2 *psae_2 *ycf35_2 *rps16_2 *rps4_2 *psb28_2 psac_2 psbw |
| *Rhizosolenia imbricata* (*l*) | *psab *psaa *psaj *psaf *psb1 *ycf39 *psal psbe psbf psbl psbj *psad *psbc *psbd *psbz *rpl12 *rpl1 *rpl11 psbx psbv rpl19 *ycf3 *rps18 *rpl33 rps20 rpob rpoc1 rpoc2_1 rps2 *psbk *rbcl psbb psbt *psbn psbh ycf33 *rps14 rpl35 rpl20 *psba_1 psac_1 rps6_1 *clpc_1 *rps10_1 *rps7_1 *rps12_1 *rpl34_1 *rpl27_1 *rpl21_1 *rpl32_1 *psby_1 *ycf33 *rps16 *rps4 *psb28_1 rpl3 rpl4 rpl23 rpl2 rps19 rpl22 rps3 rpl16 rpl29 rps17 rpl14 rpl24 rpl5 rps8 rpl6 rpl18 rps5 rpl36 rps13 rps11 rpoa rpl13 rps9 rpl31 rps12_2 rps7_2 rps10_2 clpc_2 *rps6_2 *psac_2 psba_2 |
| *Thalassiosira oceanica – Odontella sinensis* | *psaj *psaf rps14 psam rpl11 rpl1 rpl12 *rps2 *rpoc2_1 *rpoc1 *rpob *rps20 rpl33 rps18 ycf3 *psb1 *ycf39 *psal psbe psbf psbl psaa psab psae_1 ycf33 *psbc *psbd *psa1 psbk psbz *psbj *psbh psbn *psbt *psbb *ycf31 *psbh psbn *psbt *psbb rbcl *rpoc1 *rpl19 *psbv *psbx \| rpl32_1 rpl21_1 rpl27_1 rpl34_1 psba_1 rpl35_1 rpl20_1 psby_1 psac_1 rps6_1 *clpc_1 *rps10_1 *tufa *rps7 *rps12 *rpl31 *rps9 *rpl13 *rpoa *rps11 *rps13 *rpl36 *rps5 *rpl18 *rpl6 *rps8 *rpl5 *rpl24 *rpl14 *rps17 *rpl29 *rpl16 *rps3 *rpl22 *rps19 *rpl2 *rpl23 *rpl4 *rpl3 psb28_2 rps4_2 rps16_2 ycf35_2 *psba_2 *rpl34_2 *rpl27_2 *rpl21_2 *rpl32_2 *psby_2 *rpl20_2 *rpl35_2 *psae_2 *psad *ycf35_1 *rps16_1 *rps4_1 *psb28_1 psac_2 psbw |
| *Thalassiosira oceanica – Thalassiosira pseudonana* | *psaj *psaf rps14 psam rpl11 rpl1 rpl12 *rps2 *rpoc2_1 *rpoc1 *rpob *rps20 rpl33 rps18 ycf3 *psb1 *ycf39 *psal psbe psbf psbl psaa psab *rpl19 *psbv *psbx *rbcl psbb psbt *psbn psbh psae_1 rpl35_1 rpl20 *ycf33 *psbk psa1 psbd psbc psbz psad *psbj psby_2 rpl32_2 rpl21_2 rpl27_2 rpl34_2 psba_2 ycf35_2 *psac_2 *rps16 *rps4 *psbw *psb28_2 rpl3 rpl4 rpl23 rpl2 rps19 rpl22 rps3 rpl16 rpl29 rps17 rpl14 rpl24 rpl5 rps8 rpl6 rpl18 rps5 rpl36 rps13 rps11 rpoa rpl13 rps9 rpl31 rps12 rps7 tufa rps10_1 clpc_1 *rps6_1 psac_1 *ycf35_1 *psba_1 *rpl34_1 *rpl27_1 *rpl21_1 *rpl32_1 *psby_1 |
| *Thalassiosira oceanica* (*l*) | *psaj *psaf rps14 psam rpl11 rpl1 rpl12 *rps2 *rpoc2_1 *rpoc1 *rpob *rps20 rpl33 rps18 ycf3 *psb1 *ycf39 *psal psbe psbf psbl psbj *psad psbd psbc psbz *psa1 psbk ycf33 psbx psbv psbb psbt *psbn psbh psae rpl35 rpl20 *rbcl rpl19 *psab *psaa *psby_2 rpl32_2 rpl21_2 rpl27_2 rpl34_2 psac *psba_1 ycf35_1 clpc_1 psb28_1 rps4_1 rps16_1 rps6_1 rpl3 rpl4 rpl23 rpl2 rps19 rpl22 rps3 rpl16 rpl29 rps17 rpl14 rpl24 rpl5 rps8 rpl6 rpl18 rps5 rpl36 rps13 rps11 rpoa rpl13 rps9 rpl31 rps12 rps7 tufa rps10_1 *clpc' *ycf35' psba' *psac' *rpl34' *rpl27' *rpl21' *rpl32' psby' |
| *Thalassiosira weissflogii – Thalassiosira pseudonana* | psaa psab *rpl19 *psbv *psbx *rbcl psbb psbt *psbn psbh psae_1 rpl35_1 rpl20 rpl11 rpl1 rpl12 *rps2 *rpoc2_1 *rpoc1 *rpob *rps20 rpl33 rps18 ycf3 *psam *rps14 *ycf33 *psbk psa1 psbd psbc psbz psad *psbj *psbl *psbf *psbe psal ycf39 psb1 psaf psaj psby_2 rpl32_2 rpl21_2 rpl27_2 rpl34_2 psba_2 ycf35 *psac *rps16 *rps4 *psbw *psb28_2 rpl3 rpl4 rpl23 rpl2 rps19 rpl22 rps3 rpl16 rpl29 rps17 rpl14 rpl24 rpl5 rps8 rpl6 rpl18 rps5 rpl36 rps13 rps11 rpoa rpl13 rps9 rpl31 rps12 rps7 tufa rps10_1 clpc_1 *rps6_1 psac_1 *ycf35_1 *psba_1 *rpl34_1 *rpl27_1 *rpl21_1 *rpl32_1 *psby_1 |
| *Thalassiosira weissflogii – Roundia cardiophora* | psaa psab *rpl19 *psbv *psbx *rbcl psbb psbt *psbn psbh psae_1 rpl35_1 rpl20 rpl11 rpl1 rpl12 *rps2 *rpoc2_1 *rpoc1 *rpob *rps20 rpl33 rps18 ycf3 *psam *rps14 *ycf33 *psbk psa1 psbd psbc psbz psad *psbj *psbl *psbf *psbe psal ycf39 psb1 psaf psaj psby_2 rpl32_2 rpl21_2 rpl27_2 rpl34_2 psba_2 ycf35 *psac *rps16 *rps4 *psb28 rpl3 rpl4 rpl23 rpl2 rps19 rpl22 rps3 rpl16 rpl29 rps17 rpl14 rpl24 rpl5 rps8 rpl6 rpl18 rps5 rpl36 rps13 rps11 rpoa rpl13 rps9 rpl31 rps12 rps7 tufa rps10_1 clpc_1 *rps6_1 psac_1 *ycf35_1 *psba_1 *rpl34_1 *rpl27_1 *rpl21_1 *rpl32_1 *psby_1 |
| *Thalassiosira weissflogii* (*l*) | psaa psab *rpl19 *psbv *psbx *rbcl psbb psbt *psbn psbh psae_1 rpl35_1 rpl20 rpl11 rpl1 rpl12 *rps2 *rpoc2_1 *rpoc1 *rpob *rps20 rpl33 rps18 ycf3 *psam *rps14 *ycf33 *psbk psa1 psbd psbc psbz psad *psbj *psbl *psbf *psbe psal ycf39 psb1 psaf psaj psby_2 rpl32_2 rpl21_2 rpl27_2 rpl34_2 psba_2 ycf35_2 *psac_2 *rps16_2 *rps4_2 *psb28_2 rpl3 rpl4 rpl23 rpl2 rps19 rpl22 rps3 rpl16 rpl29 rps17 rpl14 rpl24 rpl5 rps8 rpl6 rpl18 rps5 rpl36 rps13 rps11 rpoa rpl13 rps9 rpl31 rps12 rps7 tufa rps10_1 clpc_1 *rps6_1 psac_1 *ycf35_1 *psba_1 *rpl34_1 *rpl27_1 *rpl21_1 *rpl32_1 *psby_1 |
| *Roundia cardiophora* (*l*) | psaa psab *rpl19 *psbv *psbx *rbcl psbb psbt *psbn psbh psae_1 rpl35_1 rpl20 rpl11 rpl1 rpl12 *rps2 *rpoc2_1 *rpoc1 *rpob *rps20 rpl33 rps18 ycf3 *psam *rps14 *ycf33 *psbk psa1 psbd psbc psbz psad *psbj *psbl *psbf *psbe psal ycf39 psb1 psaf psaj psby_2 rpl32_2 rpl21_2 rpl27_2 rpl34_2 psba_2 ycf35_2 *psac_2 *rps16_2 *rps4_2 *psb28_2 rpl3 rpl4 rpl23 rpl2 rps19 rpl22 rps3 rpl16 rpl29 rps17 rpl14 rpl24 rpl5 rps8 rpl6 rpl18 rps5 rpl36 rps13 rps11 rpoa rpl13 rps9 rpl31 rps12 rps7 tufa rps10_1 clpc_1 *rps6_1 psac_1 *ycf35_1 *psba_1 *rpl34_1 *rpl27_1 *rpl21_1 *rpl32_1 *psby_1 |
| *Thalassiosira pseudonana* (*l*) | psaa psab *rpl19 *psbv *psbx *rbcl psbb psbt *psbn psbh psae_1 rpl35_1 rpl20 rpl11 rpl1 rpl12 *rps2 *rpoc2_1 *rpoc1 *rpob *rps20 rpl33 rps18 ycf3 *psam *rps14 *ycf33 *psbk psa1 psbd psbc psbz psad *psbj *psbl *psbf *psbe psal ycf39 psb1 psaf psaj psby_2 rpl32_2 rpl21_2 rpl27_2 rpl34_2 psba_2 ycf35_2 *psac_2 *rps16_2 *rps4_2 *psbw rpl3 rpl4 rpl23 rpl2 rps19 rpl22 rps3 rpl16 rpl29 rps17 rpl14 rpl24 rpl5 rps8 rpl6 rpl18 rps5 rpl36 rps13 rps11 rpoa rpl13 rps9 rpl31 rps12 rps7 tufa rps10_1 clpc_1 *rps6_1 psac_1 *ycf35_1 *psba_1 *rpl34_1 *rpl27_1 *rpl21_1 *rpl32_1 *psby_1 |
| *Asterionellopsis glacialis – Odontella sinensis* | psaa psab psaf psaj psae_1 *ycf33 *psbc *psbd *psa1 psbk psbz *psbj *psbl *psbf *psbe psal ycf39 psb1 *psbh psbn *psbt *psbb *ycf31 *psbh psbn *psbt *psbb rbcl *rps2 *rpoc2_1 *rpoc1 *rpob *rps20 rpl33 rps18 ycf3 *rpl19 *psbv *psbx rpl11 rpl1 rpl12 \| rpl32_1 rpl21_1 rpl27_1 rpl34_1 psba_1 rpl35_1 rpl20_1 psby_1 psac_1 rps6_1 *clpc_1 *rps10_1 *tufa *rps7 *rps12 *rpl31 *rps9 *rpl13 *rpoa *rps11 *rps13 *rpl36 *rps5 *rpl18 *rpl6 *rps8 *rpl5 *rpl24 *rpl14 *rps17 *rpl29 *rpl16 *rps3 *rpl22 *rps19 *rpl2 *rpl23 *rpl4 *rpl3 psb28_2 rps4_2 rps16_2 ycf35_2 *psba_2 *rpl34_2 *rpl27_2 *rpl21_2 *rpl32_2 *psby_2 *rpl20_2 *rpl35_2 *psae_2 rps14 psam *psad *ycf35_1 *rps16_1 *rps4_1 *psb28_1 psac_2 psbw |
| *Asterionellopsis glacialis* (*l*) | psaa psab psaf psaj psae_1 ycf33 *psbc *psbd *psa1 psbk psbz *psbj *psbl *psbf *psbe psal ycf39 psb1 *psbh psbn *psbt *psbb rps14 psam psad *rpl12 *rpl1 *rpl11 psbx psbv rpl19 *rps2 *rpoc2_1 *rpoc1 *rpob *rps20 rpl33 rps18 ycf3 *rbcl rpl35_2 rpl20_2 psby_2 rpl32_2 *psac_2 rpl3 rpl4 rpl23 rpl2 rps19 rpl22 rps3 rpl16 rpl29 rps17 rpl14 rpl24 rpl5 rps8 rpl6 rpl18 rps5 rpl36 rps13 rps11 rpoa rpl13 rps9 rpl31 rps12 rps7 tufa rps10_1 rpl21_1 rpl27_1 rpl34_1 psba_1 psb28_1 rps4_1 rps16_1 ycf35_1 clpc_1 *rps6_1 *rpl32_1 *psby_1 |
| *Cylindrotheca closterium – Odontella sinensis* | psbz *psbj *psbl *psbf *psbe psal ycf39 psb1 psaf psaj *ycf31 *psbh psbn *psbt *psbb rbcl ycf33 *rps2 *rpoc2_1 *rpoc1 *rpob *rps20 rpl33 rps18 ycf3 *rpl19 *psbv *psbx rpl11 rpl1 rpl12 *psbk psa1 psbd psbc \| rpl32_1 rpl21_1 rpl27_1 rpl34_1 psba_1 psae_1 rpl35_1 rpl20_1 psby_1 psac_1 rps6_1 *clpc_1 *rps10_1 *tufa *rps7 *rps12 *rpl31 *rps9 *rpl13 *rpoa *rps11 *rps13 *rpl36 *rps5 *rpl18 *rpl6 *rps8 *rpl5 *rpl24 *rpl14 *rps17 *rpl29 *rpl16 *rps3 *rpl22 *rps19 *rpl2 *rpl23 *rpl4 *rpl3 psb28_2 rps4_2 rps16_2 ycf35_2 *psba_2 *rpl34_2 *rpl27_2 *rpl21_2 *rpl32_2 *psby_2 *rpl20_2 *rpl35_2 *psae_2 rps14 psam *psad *psab *psaa *ycf35_1 *rps16_1 *rps4_1 *psb28_1 psac_2 psbw |
| *Cylindrotheca closterium – Phaeodactylum tricornutum* | *psaa ycf33 *rpoc2_1 *rpoc1 *rpob *rps20 rpl33 rps18 ycf3 *rpl19 *psbv *psbx rpl11 rpl1 rpl12 psad *psam *rps14 psbb psbt *psbn psbh psab *psaj *psaf *psb1 *ycf39 *psal psbe psbf psbl psbj *psbz *psbc *psbd *psa1 psbk rpl32_1 rpl21_1 rpl27_1 rpl34_1 psba_1 *ycf35_1 *rps16_1 *rps4_1 *psb28_1 \| psb28_2 rps4_2 rps16_2 ycf35_2 *psba_2 *rpl34_2 *rpl27_2 *rpl21_2 *rpl32_2 *psby_2 *rpl20_2 *rpl35_2 *psae_2 rps2 *rbcl psae_1 rpl35_1 rpl20_1 psby_1 psac_1 rps6_1 *clpc_1 *rps10_1 *tufa *rps7 *rps12 *rpl31 *rps9 *rpl13 *rpoa *rps11 *rps13 *rpl36 *rps5 *rpl18 *rpl6 *rps8 *rpl5 *rpl24 *rpl14 *rps17 *rpl29 *rpl16 *rps3 *rpl22 *rps19 *rpl2 *rpl23 *rpl4 *rpl3 |
| *Cylindrotheca closterium* (*l*) | *psaa ycf33 *rpoc2_1 *rpoc1 *rpob *rps20 rpl33 rps18 ycf3 *rpl19 *psbv *psbx rpl11 rpl1 rpl12 psad *psam *rps14 psbb psbt *psbn psbh *psaj *psaf *psb1 *ycf39 *psal psbe psbf psbl psbj *psbz *psbk psa1 psbd psbc *rbcl *psae_1 rps2 rpl35_1 rpl20_1 *clpc_1 *rps6_1 rpl3 rpl4 rpl23 rpl2 rps19 rpl22 rps3 rpl16 rpl29 rps17 rpl14 rpl24 rpl5 rps8 rpl6 rpl18 rps5 rpl36 rps13 rps11 rpoa rpl13 rps9 rpl31 rps12 rps7 tufa rps10_1 *ycf35_1 *rps16_1 *rps4_1 *psb28_1 *psba_1 *rpl34_1 *rpl27_1 *rpl21_1 *rpl32_1 *psac_1 *psby_1 *psab |
| *Lithodesmium undulatum – Phaeodactylum tricornutum* | psaa psab *psaj *psaf *psb1 *ycf39 *psal psbe psbf psbl psbj *psbz *psbc *psbd *psa1 psbk rps14 psam psad *psbv *psbx rpl32_1 rpl21_1 rpl27_1 rpl34_1 psba_1 *ycf35_1 *rps16_1 *rps4_1 *psb28_1 \| psb28_2 rps4_2 rps16_2 ycf35_2 *psba_2 *rpl34_2 *rpl27_2 *rpl21_2 *rpl32_2 *psby_2 *rpl20_2 *rpl35_2 *psae_2 *ycf33 *psbh psbn *psbt *psbb *rpl12 *rpl1 *rpl11 rpl19 *ycf3 *rps18 *rpl33 rps20 rpob rpoc1 rpoc2_1 rps2 *rbcl psae_1 rpl35_1 rpl20_1 psby_1 psac_1 rps6_1 *clpc_1 *rps10_1 *tufa *rps7 *rps12 *rpl31 *rps9 *rpl13 *rpoa *rps11 *rps13 *rpl36 *rps5 *rpl18 *rpl6 *rps8 *rpl5 *rpl24 *rpl14 *rps17 *rpl29 *rpl16 *rps3 *rpl22 *rps19 *rpl2 *rpl23 *rpl4 *rpl3 |
| *Lithodesmium undulatum – Coscinodiscus radiates* | psaa psab *psaj *psaf *psb1 *ycf39 *psal psbe psbf psbl psbj *psbz *psbc *psbd *psa1 psbk rps14 psam psad *psbv *psbx rpl11 rpl1 rpl12 rpl19 *ycf3 *rps18 *rpl33 rps20 rpob rpoc1 rpoc2_1 rps2 *ycf33 *psbh psbn *psbt *psbb rbcl psae_2 psby_2 rpl35_2 rpl20_2 *psba_2 *rpl34_2 *rpl27_2 *rpl21_2 *rpl32_2 *psby_2 ycf35_2 *rps16_2 *rps4_2 *psb28_2 rpl3 rpl4 rpl23 rpl2 rps19 rpl22 rps3 rpl16 rpl29 rps17 rpl14 rpl24 rpl5 rps8 rpl6 rpl18 rps5 rpl36 rps13 rps11 rpoa rpl13 rps9 rpl31 rps12 rps7 tufa rps10_1 clpc_1 *rps6_1 *psac_1 psba_1 *rpl20_1 *rpl35_1 *psae_1 |
| *Lithodesmium undulatum* (*l*) | psaa psab *psaj *psaf *psb1 *ycf39 *psal psbe psbf psbl psbj *psbz *psbc *psbd *psa1 psbk rps14 psam psad *psbv *psbx rpl11 rpl1 rpl12 rpl19 *ycf3 *rps18 *rpl33 rps20 rpob rpoc1 rpoc2_1 rps2 *ycf33 *psbh psbn *psbt *psbb rbcl *rpl20_2 *rpl35_2 *psae_2 psby_2 *rps16_2 *rps4_2 *psb28_2 rpl3 rpl4 rpl23 rpl2 rps19 rpl22 rps3 rpl16 rpl29 rps17 rpl14 rpl24 rpl5 rps8 rpl6 rpl18 rps5 rpl36 rps13 rps11 rpoa rpl13 rps9 rpl31 rps12 rps7 tufa rps10_1 clpc_1 *rps6_1 *psac_1 ycf35_1 rpl32_1 rpl21_1 rpl27_1 rpl34_1 psba_1 *psby_1 psae_1 rpl35_1 rpl20_1 |
| *Coscinodiscus radiates* (*l*) | psaa psab *psaj *psaf *psb1 *ycf39 *psal psbe psbf psbl psbj *psbz *psbc *psbd psam psad *rpl12 *rpl1 *rpl11 psbx psbv rpl19 *ycf3 *rps18 *rpl33 rps20 rpob rpoc1 rpoc2_1 rps2 *ycf33 *psbh psbn *psbt *psbb rbcl *psa1 psbk *rps14 psae_2 rpl35_2 rpl20_2 *psba_2 *rpl34_2 *rpl27_2 *rpl21_2 *rpl32_2 *psby_2 ycf35_2 *rps16_2 *rps4_2 *psb28_2 rpl3 rpl4 rpl23 rpl2 rps19 rpl22 rps3 rpl16 rpl29 rps17 rpl14 rpl24 rpl5 rps8 rpl6 rpl18 rps5 rpl36 rps13 rps11 rpoa rpl13 rps9 rpl31 rps12 rps7 tufa rps10_1 clpc_1 *rps6_1 *psac_1 psba_1 *rpl20_1 *rpl35_1 *psae_1 |
| *Cerataulina daemon – Phaeodactylum tricornutum* | rpl32_1 rpl21_1 rpl27_1 rpl34_1 psba_1 *ycf35_1 *rps16_1 *rps4_1 *psb28_1 \| psb28_2 rps4_2 rps16_2 ycf35_2 *psba_2 *rpl34_2 *rpl27_2 *rpl21_2 *rpl32_2 *psby_2 *rpl20_2 *rpl35_2 *psae_2 *psab *psaa *psaj *psaf *psb1 *ycf39 *psal psbe psbf psbl psbj *psbz *psbc *psbd *psa1 psbk *ycf33 *psbh psbn *psbt *psbb rps14 psam *psad *rpl12 *rpl1 *rpl11 psbx psbv rpl19 *ycf3 *rps18 *rpl33 rps20 rpob rpoc1 rpoc2_1 rps2 *rbcl psae_1 rpl35_1 rpl20_1 psby_1 psac_1 rps6_1 *clpc_1 *rps10_1 *tufa *rps7 *rps12 *rpl31 *rps9 *rpl13 *rpoa *rps11 *rps13 *rpl36 *rps5 *rpl18 *rpl6 *rps8 *rpl5 *rpl24 *rpl14 *rps17 *rpl29 *rpl16 *rps3 *rpl22 *rps19 *rpl2 *rpl23 *rpl4 *rpl3 |
| *Cerataulina daemon – Chaetoceros simplex* | ycf35_1 psb28_1 rps4_1 rps16_1 \| psb28_2 rps4_2 rps16_2 ycf35_2 *psba_2 *rpl34_2 *rpl27_2 *rpl21_2 *rpl32_2 *psby_2 *rpl20_2 *rpl35_2 *psae_2 psaa psab ycf33 psad *psam *rps14 psae_1 rpl35_1 rpl20_1 psby_1 psac_1 rps6_1 *clpc_1 *rps10_1 *tufa *rps7 *rps12 *rpl31 *rps9 *rpl13 *rpoa *rps11 *rps13 *rpl36 *rps5 *rpl18 *rpl6 *rps8 *rpl5 *rpl24 *rpl14 *rps17 *rpl29 *rpl16 *rps3 *rpl22 *rps19 *rpl2 *rpl23 *rpl4 *rpl3 \| rbcl *rps2 *rpoc2_1 *rpoc1 *rpob *rps20 rpl33 rps18 ycf3 *rpl19 *psbv *psbx rpl11 rpl1 rpl12 *psbk psa1 psbd psbc psbz *psbj *psbl *psbf *psbe psal ycf39 psb1 psaf psaj *psbh psbn *psbt *psbb C |
| *Cerataulina daemon* (*l*) | psaa psab ycf33 *rps2 *rpoc2_1 *rpoc1 *rpob *rps20 rpl33 rps18 ycf3 *rpl19 *psbv *psbx rpl11 rpl1 rpl12 *psbk psa1 psbd psbc psbz *psbj *psbl *psbf *psbe psal ycf39 psb1 psaf psaj *psbh psbn *psbt *psbb rbcl *psad *psam *rps14 psae_2 rpl35_2 rpl20_2 psby_2 rpl32_2 rpl21_2 rpl27_2 rpl34_2 psba_2 *ycf35_2 *rps16_2 *rps4_2 *psb28_2 rpl3 rpl4 rpl23 rpl2 rps19 rpl22 rps3 rpl16 rpl29 rps17 rpl14 rpl24 rpl5 rps8 rpl6 rpl18 rps5 rpl36 rps13 rps11 rpoa rpl13 rps9 rpl31 rps12 rps7 tufa rps10_1 clpc_1 *rps6_1 *psac_1 *psby_1 |
| *Chaetoceros simplex* (*l*) | *psad *rps2 *rpoc2_1 *rpoc1 *rpob *rps20 rpl33 rps18 psaa psab ycf3 *rpl19 *psbv *psbx rpl11 rpl1 rpl12 *psbk psa1 psbd psbc psbz *psb1 *ycf39 *psal psbe psbf psbl psbj psaf psaj *psbh psbn *psbt *psbb rbcl *ycf33 *psam *rps14 psae_1 rpl35_1 rpl20_1 psby_1 *ycf35_1 *rps16_1 *rps4_1 *psb28_1 rpl3 rpl4 rpl23 rpl2 rps19 rpl22 rps3 rpl16 rpl29 rps17 rpl14 rpl24 rpl5 rps8 rpl6 rpl18 rps5 rpl36 rps13 rps11 rpoa rpl13 rps9 rpl31 rps12 rps7 tufa rps10_2 clpc_2 *rps6_2 *psac_2 *psba_2 *rpl34_2 *rpl27_2 *rpl21_2 *rpl32_2 *psby_2 |
| *Asterionella Formosa – Phaeodactylum tricornutum* | rpl32_1 rpl21_1 rpl27_1 rpl34_1 psba_1 *ycf35_1 *rps16_1 *rps4_1 *psb28_1 \| psb28_2 rps4_2 rps16_2 ycf35_2 *psba_2 *rpl34_2 *rpl27_2 *rpl21_2 *rpl32_2 *psby_2 *rpl20_2 *rpl35_2 *psae_2 *psab *psaa *psaj *psaf *psb1 *ycf39 *psal psbe psbf psbl psbj *psbz *psbc *psbd *psa1 psbk *ycf33 *psbh psbn *psbt *psbb rps14 psam *psad *rpl12 *rpl1 *rpl11 psbx psbv rpl19 *ycf3 *rps18 *rpl33 rps20 rpob rpoc1 rpoc2_1 rps2 *rbcl psae_1 rpl35_1 rpl20_1 psby_1 psac_1 rps6_1 *clpc_1 *rps10_1 *tufa *rps7 *rps12 *rpl31 *rps9 *rpl13 *rpoa *rps11 *rps13 *rpl36 *rps5 *rpl18 *rpl6 *rps8 *rpl5 *rpl24 *rpl14 *rps17 *rpl29 *rpl16 *rps3 *rpl22 *rps19 *rpl2 *rpl23 *rpl4 *rpl3 |
| *Asterionella Formosa – Ulnaria acus* | psb28_2 rps4_2 rps16_2 ycf35_2 *psba_2 *rpl34_2 *rpl27_2 *rpl21_2 *rpl32_2 *psby_2 *rpl20_2 *rpl35_2 *psae_2 *psab *psaa *psaj *psaf *psb1 *ycf39 *psal psbe psbf psbl psbj *psbz *psbc *psbd *psa1 psbk *ycf33 *psbh psbn *psbt *psbb rps14 psam psad *rpl12 *rpl1 *rpl11 psbx psbv rpl19 *ycf3 *rps18 *rpl33 rps20 rpob rpoc1 rpoc2_1 rps2 rbcl psae_1 rpl35_1 rpl20_1 psby_1 psac_1 rps6_1 *clpc_1 *rps10_1 *tufa *rps7 *rps12 *rpl31 *rps9 *rpl13 *rpoa *rps11 *rps13 *rpl36 *rps5 *rpl18 *rpl6 *rps8 *rpl5 *rpl24 *rpl14 *rps17 *rpl29 *rpl16 *rps3 *rpl22 *rps19 *rpl2 *rpl23 *rpl4 *rpl3 |
| *Asterionella Formosa* (*l*) | psaa psab *rbcl *rps2 *rpoc2_1 *rpoc1 *rpob *rps20 rpl33 rps18 ycf3 *rpl19 *psbv *psbx rpl11 rpl1 rpl12 *psbh psbn *psbt *psbb rps14 psam psad ycf33 *psbk psa1 psbd psbc psbz *psbj *psbl *psbf *psbe psal ycf39 psb1 psaf psaj psae_2 rpl35_2 rpl20_2 psby_2 psac_2 rpl32_2 rpl21_2 rpl27_2 rpl34_2 psba_2 *ycf35_2 *rps16_2 *rps4_2 *psb28_2 rpl3 rpl4 rpl23 rpl2 rps19 rpl22 rps3 rpl16 rpl29 rps17 rpl14 rpl24 rpl5 rps8 rpl6 rpl18 rps5 rpl36 rps13 rps11 rpoa rpl13 rps9 rpl31 rps12 rps7 tufa rps10_1 rps6_1 *clpc_1 *psac_1 *psby_1 |
| *Ulnaria acus* (*l*) | psaa psab *psaj *psaf *psb1 *ycf39 *psal psbe psbf psbl psbj *psbz *ycf33 psbd psbc *psbk psa1 *psbh psbn *psbt *psbb rps14 psam psad *rpl12 *rpl1 *rpl11 psbx psbv rpl19 *ycf3 *rps18 *rpl33 rps20 rpob rpoc1 rpoc2_1 rps2 rbcl psae_1 rpl35_1 rpl20_1 psby_1 psac_1 rps6_1 *clpc_1 *rps10_1 *tufa *rps7 *rps12 *rpl31 *rps9 *rpl13 *rpoa *rps11 *rps13 *rpl36 *rps5 *rpl18 *rpl6 *rps8 *rpl5 *rpl24 *rpl14 *rps17 *rpl29 *rpl16 *rps3 *rpl22 *rps19 *rpl2 *rpl23 *rpl4 *rpl3 psb28_2 rps4_2 rps16_2 ycf35_2 *psba_2 *rpl34_2 *rpl27_2 *rpl21_2 *rpl32_2 *psby_2 |
| *Durinskia baltica – Phaeodactylum tricornutum* | rpl32_1 rpl21_1 rpl27_1 rpl34_1 psba_1 *ycf35_1 *rps16_1 *rps4_1 *psb28_1 \| psb28_2 rps4_2 rps16_2 ycf35_2 *psba_2 *rpl34_2 *rpl27_2 *rpl21_2 *rpl32_2 *psby_2 *rpl20_2 *rpl35_2 *psae_2 *psab *psaa *psaj *psaf *psb1 *ycf39 *psal psbe psbf psbl psbj *psbz *psbc *psbd *psa1 psbk *ycf33 *psbh psbn *psbt *psbb rps14 psam *psad *rpl12 *rpl1 *rpl11 psbx psbv rpl19 *ycf3 *rps18 *rpl33 rps20 rpob rpoc1 rpoc2_1 rps2 *rbcl psae_1 rpl35_1 rpl20_1 psby_1 psac_1 rps6_1 *clpc_1 *rps10_1 *tufa *rps7 *rps12 *rpl31 *rps9 *rpl13 *rpoa *rps11 *rps13 *rpl36 *rps5 *rpl18 *rpl6 *rps8 *rpl5 *rpl24 *rpl14 *rps17 *rpl29 *rpl16 *rps3 *rpl22 *rps19 *rpl2 *rpl23 *rpl4 *rpl3 |
| *Durinskia baltica – Kryptoperidinium foliaceum* | rpl32_1 rpl21_1 rpl27_1 rpl34_1 psba_1 *ycf35_1 *rps16_1 *rps4_1 *psb28_1 rps6_1 *clpc_1 *rps10_1 *tufa *rps7 *rps12 *rpl31 *rps9 *rpl13 *rpoa *rps11 *rps13 *rpl36 *rps5 *rpl18 *rpl6 *rps8 *rpl5 *rpl24 *rpl14 *rps17 *rpl29 *rpl16 *rps3 *rpl22 *rps19 *rpl2 *rpl23 *rpl4 *rpl3 *rpl20_1 *rpl35_1 *psae_1 rbcl *rps2 *rpoc2_1 *rpoc1 *rpob *rps20 rpl33 rps18 ycf3 *rpl19 *psbv *psbx rpl11 rpl1 rpl12 psad *psam *rps14 psbb psbt *psbn psbh ycf33 *psbk psa1 psbd psbc psbz *psbj *psbl *psbf *psbe psal ycf39 psb1 psaf psaj psaa psab psby_1 psac_1 |
| *Durinskia baltica* (*l*) | *psab *psaa *psaj *psaf *psb1 *ycf39 *psal psbe psbf psbl psbj *psbz *psbc *psbd *psa1 psbk *ycf33 *psbh psbn *psbt *psbb rps14 psam *psad *rpl12 *rpl1 *rpl11 psbx psbv rpl19 *ycf3 *rps18 *rpl33 rps20 rpob rpoc1 rpoc2_1 rps2 *rbcl psae_1 *rpl20_1 *rpl35_1 rps6_1 psby_1 psac_1 rpl32_1 rpl21_1 rpl27_1 rpl34_1 psba_1 *ycf35_1 *rps16_1 *rps4_1 *psb28_1 rpl3 rpl4 rpl23 rpl2 rps19 rpl22 rps3 rpl16 rpl29 rps17 rpl14 rpl24 rpl5 rps8 rpl6 rpl18 rps5 rpl36 rps13 rps11 rpoa rpl13 rps9 rpl31 rps12 rps7 tufa rps10_1 clpc_1 |
| *Kryptoperidinium foliaceum* (*l*) | *psbj *psbl *psbf *psbe psal ycf39 psb1 psaf psaj psaa psab *psbz *psbc *psbd *psa1 psbk *ycf33 *psbh psbn *psbt *psbb rps14 psam *psad *rpl12 *rpl1 *rpl11 psbx psbv rpl19 *ycf3 *rps18 *rpl33 rps20 rpob rpoc1 rpoc2_1 rps2 *rbcl psae_1 rpl35_1 rpl20_1 rpl3 rpl4 rpl23 rpl2 rps19 rpl22 rps3 rpl16 rpl29 rps17 rpl14 rpl24 rpl5 rps8 rpl6 rpl18 rps5 rpl36 rps13 rps11 rpoa rpl13 rps9 rpl31 rps12 rps7 tufa rps10_1 clpc_1 *rps6_1 psb28_1 rps4_1 rps16_1 ycf35_1 *psba_1 *rpl34_1 *rpl27_1 *rpl21_1 *rpl32_1 *psac_1 *psby_1 |
| *Fistulifera solaris – Phaeodactylum tricornutum* | rpl32_1 rpl21_1 rpl27_1 rpl34_1 psba_1 *ycf35_1 *rps16_1 *rps4_1 *psb28_1 psac_1 *rps6_1 *clpc_1 *rps10_1 *tufa *rps7 *rps12 *rpl31 *rps9 *rpl13 *rpoa *rps11 *rps13 *rpl36 *rps5 *rpl18 *rpl6 *rps8 *rpl5 *rpl24 *rpl14 *rps17 *rpl29 *rpl16 *rps3 *rpl22 *rps19 *rpl2 *rpl23 *rpl4 *rpl3 psb28_2 rps4_2 rps16_2 ycf35_2 *psba_2 *rpl34_2 *rpl27_2 *rpl21_2 *rpl32_2 *psby_2 *rpl20_2 *rpl35_2 *psae_2 *psab *psaa *psaj *psaf *psb1 *ycf39 *psal psbe psbf psbl psbj *psbz *psbc *psbd *psa1 psbk *ycf33 *psbh psbn *psbt *psbb rps14 psam *psad *rpl12 *rpl1 *rpl11 psbx psbv rpl19 *ycf3 *rps18 *rpl33 rps20 rpob rpoc1 rpoc2_1 rps2 *rbcl psae_1 rpl35_1 rpl20_1 psby_1 |
| *Fistulifera solaris* (*l*) | *rpob *rps20 rpl33 rps18 ycf3 *rpl19 *psbv *psbx rpl11 rpl1 rpl12 psad *psam *rps14 psae_1 rpl35_1 rpl20_1 psby_1 psac_1 *rps6_1 *clpc_1 *rps10_1 *tufa *rps7 *rps12 *rpl31 *rps9 *rpl13 *rpoa *rps11 *rps13 *rpl36 *rps5 *rpl18 *rpl6 *rps8 *rpl5 *rpl24 *rpl14 *rps17 *rpl29 *rpl16 *rps3 *rpl22 *rps19 *rpl2 *rpl23 *rpl4 *rpl3 psb28_2 rps4_2 rps16_2 ycf35_2 *psba_2 *rpl34_2 *rpl27_2 *rpl21_2 *rpl32_2 *psab *psaa *psaj *psaf *ycf39 *psal psbe psbf psbl psbj *psbz *psbc *psbd *psa1 psbk *ycf33 *psbh psbn *psbt *psbb *rbcl *rps2 *rpoc2_1 *rpoc1 |
| *Eunotia naegelii – Phaeodactylum tricornutum* | rpl32_1 rpl21_1 rpl27_1 rpl34_1 psba_1 *ycf35_1 *rps16_1 *rps4_1 *psb28_1 psac_1 *rps6_1 *clpc_1 *rps10_1 *tufa *rps7 *rps12 *rpl31 *rps9 *rpl13 *rpoa *rps11 *rps13 *rpl36 *rps5 *rpl18 *rpl6 *rps8 *rpl5 *rpl24 *rpl14 *rps17 *rpl29 *rpl16 *rps3 *rpl22 *rps19 *rpl2 *rpl23 *rpl4 *rpl3 psb28_2 rps4_2 rps16_2 ycf35_2 *psba_2 *rpl34_2 *rpl27_2 *rpl21_2 *rpl32_2 *psby_2 *rpl20_2 *rpl35_2 *psae_2 rbcl *rps2 *rpoc2_1 *rpoc1 *rpob *rps20 rpl33 rps18 ycf3 *rpl19 *psbv *psbx rpl11 rpl1 rpl12 psad *psam *rps14 psbb psbt *psbn psbh ycf33 *psbk psa1 psbd psbc psbz *psbj *psbl *psbf *psbe psal ycf39 psb1 psaf psaj psaa psab psae_1 rpl35_1 rpl20_1 psby_1 |
| *Eunotia naegelii* (*l*) | psaa psab *psaj *psaf *psb1 *ycf39 *psal psbe psbf psbl psbj *psbz *psbc *psbd *psa1 psbk *ycf33 *psbh psbn *psbt *psbb rps14 psam *psad *rpl12 *rpl1 *rpl11 psbx psbv rpl19 *ycf3 *rps18 *rpl33 rps20 rpob rpoc1 rpoc2_1 rps2 *rbcl psae_2 rpl35_2 rpl20_2 psby_2 rpl32_2 rpl21_2 rpl27_2 rpl34_2 psba_2 *ycf35_2 *rps16_2 *rps4_2 *psb28_2 rpl3 rpl4 rpl23 rpl2 rps19 rpl22 rps3 rpl16 rpl29 rps17 rpl14 rpl24 rpl5 rps8 rpl6 rpl18 rps5 rpl36 rps13 rps11 rpoa rpl13 rps9 rpl31 rps12 rps7 tufa rps10_1 clpc_1 *rps6_1 *psac_1 psb28_1 rps4_1 rps16_1 ycf35_1 *psba_1 *rpl34_1 *rpl27_1 *rpl21_1 *rpl32_1 *psby_1 |
| *Didymosphenia geminate – Phaeodactylum tricornutum* | psb28_2 rps4_2 rps16_2 ycf35_2 *psba_2 *rpl34_2 *rpl27_2 *rpl21_2 *rpl32_2 *psby_2 *rpl20_2 *rpl35_2 *psae_2 rbcl *rps2 *rpoc2_1 *rpoc1 *rpob *rps20 rpl33 rps18 ycf3 *rpl19 *psbv *psbx rpl11 rpl1 rpl12 psad *psam *rps14 psbb psbt *psbn psbh ycf33 *psbk psa1 psbd psbc psbz *psbj *psbl *psbf *psbe psal ycf39 psb1 psaf psaj psaa psab psae_1 rpl35_1 rpl20_1 psby_1 psac_1 *rps6_1 *clpc_1 *rps10_1 *tufa *rps7 *rps12 *rpl31 *rps9 *rpl13 *rpoa *rps11 *rps13 *rpl36 *rps5 *rpl18 *rpl6 *rps8 *rpl5 *rpl24 *rpl14 *rps17 *rpl29 *rpl16 *rps3 *rpl22 *rps19 *rpl2 *rpl23 *rpl4 *rpl3 |
| *Didymosphenia geminate* (*l*) | *psab *psaa *psaj *psaf *psb1 *ycf39 *psal psbe psbf psbl psbj *psbz *psbc *psbd *psa1 psbk *ycf33 *psbh psbn *psbt *psbb rps14 psam *psad *rpl12 *rpl1 *rpl11 psbx psbv rpl19 *ycf3 *rps18 *rpl33 rps20 rpob rpoc1 rpoc2_1 rps2 *rbcl psae_1 rpl35_1 rpl20_1 psby_1 psac_1 *rps6_1 *clpc_1 *rps10_1 *tufa *rps7 *rps12 *rpl31 *rps9 *rpl13 *rpoa *rps11 *rps13 *rpl36 *rps5 *rpl18 *rpl6 *rps8 *rpl5 *rpl24 *rpl14 *rps17 *rpl29 *rpl16 *rps3 *rpl22 *rps19 *rpl2 *rpl23 *rpl4 *rpl3 psb28_2 rps4_2 rps16_2 ycf35_2 *psba_2 *rpl34_2 *rpl27_2 *rpl21_2 *rpl32_2 *psby_2 |
| *Phaeodactylum tricornutum* (*l*) | *psab *psaa *psaj *psaf *psb1 *ycf39 *psal psbe psbf psbl psbj *psbz *psbc *psbd *psa1 psbk *ycf33 *psbh psbn *psbt *psbb rps14 psam *psad *rpl12 *rpl1 *rpl11 psbx psbv rpl19 *ycf3 *rps18 *rpl33 rps20 rpob rpoc1 rpoc2_1 rps2 *rbcl psae_2 rpl35_2 rpl20_2 psby_2 rpl32_2 rpl21_2 rpl27_2 rpl34_2 psba_2 *ycf35_2 *rps16_2 *rps4_2 *psbw rpl3 rpl4 rpl23 rpl2 rps19 rpl22 rps3 rpl16 rpl29 rps17 rpl14 rpl24 rpl5 rps8 rpl6 rpl18 rps5 rpl36 rps13 rps11 rpoa rpl13 rps9 rpl31 rps12 rps7 tufa rps10_1 clpc_1 rps6_1 *psac_1 *psby_1 |
| *Odontella sinensis* (*l*) | psby_1 rpl32_1 psac_1 rps6_1 *clpc_1 *rps10_1 *tufa *rps7 *rps12 *rpl31 *rps9 *rpl13 *rpoa *rps11 *rps13 *rpl36 *rps5 *rpl18 *rpl6 *rps8 *rpl5 *rpl24 *rpl14 *rps17 *rpl29 *rpl16 *rps3 *rpl22 *rps19 *rpl2 *rpl23 *rpl4 *rpl3 psbw rps4_2 rps16_2 ycf35_2 *psba_2 *rpl34_2 *rpl27_2 *rpl21_2 *rpl32_2 *psby_2 *rpl20_2 *rpl35_2 *psae_2 rps14 psam psad ycf33 *rbcl psbb psbt *psbn psbh ycf31 *psaj *psaf *psb1 *ycf39 *psal psbe psbf psbl psbj *psbc *psbd *psa1 psbk *rpl12 *rpl1 *rpl11 psbx psbv rpl19 *ycf3 *rps18 *rpl33 rps20 rpob rpoc1 rpoc2_1 rps2 *psab *psaa |

**Table S3*b***. **Reconstruction of chromosome structures in plastids of rhodophytic branch along the large tree.** Designations are the same as in Table S3*a*.

| *Porphyra purpyrea* *–* *Vaucheria litorea* | psac *psak *psba psby_2 rpl32 rpl21 rpl27 rps6 psbd psbc rps16 psbw rps1 *rpl12 *rpl1 *rpl11 *rpoz ycf33 *rpl19 clpc_1 *rpl9 \| rps4 *rpl28 rbcl *psbv *psbx *psaj *psaf *ycf37 *rpl34 psam psby_1 rbcl29 \| rpl33 rps18 ycf3 *ycf39 *rps2 *rpoc2_1 *rpoc1 *rpob *rps20 *psb28 ycf36 psad psb1 psal psbk *rpl20 *rpl35 *ycf35 *ycf31 *rps10 *tufa *rps12 *rpoa *rps19 *rpl2 *rpl3 *rps14 rpl23 rpl22 rps3 rpl16 rpl29 rps17 rpl14 rpl24 rpl5 rps8 rpl6 rpl18 rps5 ycf38 psbb psbt *psbn psbh *psbz psbm *psb30 psae psbe psbf psbl psbj *rpl4 *rps7 *rpl31 *rps9 *rpl13 *rps11 *rps13 *rpl36 *psa1 psaa psab |
| --- | --- |
| *Porphyra purpyrea* *–* *Cyanidioschyzon merolae* | psac *psak *psba psby_2 rpl32 rpl21 rpl27 rps6 psbd psbc rps16 psbw rps1 *rpl12 *rpl1 *rpl11 *rpoz ycf33 *rpl19 clpc_1 *rpl9 \| rps4 *rpl28 rbcl *psbv *psbx *psaj *psaf *ycf37 *rpl34 psam \| psal *psb1 *ycf39 *rps2 *rpoc2_1 *rpoc1 *rpob *rps20 rpl33 rps18 ycf3 *rpl20 *rpl35 ycf31 ycf35 psb28 ycf36 psad *psbz psbk *rps14 *psab *psaa rpl3 rpl4 rpl23 rpl2 rps19 rpl22 rps3 rpl16 rpl29 rps17 rpl14 rpl24 rpl5 rps8 rpl6 rpl18 rps5 rpl36 rps13 rps11 rpoa rpl13 rps9 rpl31 rps12 rps7 tufa rps10 ycf38 psbb psbt *psbn psbh *psae psb30 psa1 *psbj *psbl *psbf *psbe |
| *Cyanidium caldarium* –*Cyanidioschyzon merolae* | psac *psak *psba *rpl32 *psby_2 *rpl27 *rpl21 ycf39 psb1 *psal psbe psbf psbl psbj *psa1 *rps2 *rps1 *rpoc2_1 *rpoc1 *rpob *rps20 rpl33 rps18 *psbv *psbx *psaj *psaf *ycf37 psam rps4 *rpl28 rbcl ycf3 ycf33 *rpl19 clpc_1 rpl11 rpl1 rpl12 *psbw *rps16 *psbc *psbd *rps6 *rpl34 *rpoz \| rpl20 rpl3 rpl4 rpl23 rpl2 rps19 rpl22 rps3 rpl16 rpl29 rps17 rpl14 rpl24 rpl5 rps8 rpl6 rpl18 rps5 rpl36 rps13 rps11 rpoa rpl13 rps9 rpl31 rps12 rps7 tufa rps10 ycf38 psbb psbt *psbn psbh *psae psaa psab rps14 *psbk psbz *psad rpl35 |
| *Cyanidium caldarium* (*l*) | psbd psbc ycf3 *rps18 *rpl33 rpob rpoc1 rpoc2_1 rps2 psa1 *psbj *psbl *psbf *psbe psal *psb1 *ycf39 rpl21 rpl27 psby_2 rpl32 psba psak *psac rpl34 rps6 psae *psbh psbn *psbt *psbb *rpl19 clpc_1 rpl11 rpl1 rpl12 *psbw *rps16 rps4 *rpl28 rbcl *psam ycf37 psaf psaj psbv *rps10 *tufa *rps7 *rps12 *rpl31 *rps9 *rpl13 *rpoa *rps11 *rps13 *rpl36 *rps5 *rpl18 *rpl6 *rps8 *rpl5 *rpl24 *rpl14 *rps17 *rpl29 *rpl16 *rps3 *rpl22 *rps19 *rpl2 *rpl23 *rpl4 *rpl3 *rpl20 *rpl35 psad psbk *rps14 *psab *psaa |
| *Cyanidioschyzon merolae* (*l*) | rps4 *rpl28 rbcl psam rpl21 rpl27 psby_2 rpl32 psba *psak *psac rpoz rpl34 rps6 psbd psbc rps16 psbw rps1 rpl11 rpl1 rpl12 *clpc_1 rpl19 *ycf33 psbb psbt *psbn psbh *psae psaa psab rps14 *psbk psbz *psad rpl35 rpl20 rpl3 rpl4 rpl23 rpl2 rps19 rpl22 rps3 rpl16 rpl29 rps17 rpl14 rpl24 rpl5 rps8 rpl6 rpl18 rps5 rpl36 rps13 rps11 rpoa rpl13 rps9 rpl31 rps12 rps7 tufa rps10 ycf38 *ycf3 psaf psaj psbx psbv *rps18 *rpl33 rps20 rpob rpoc1 rpoc2_1 rps2 psa1 *psbj *psbl *psbf *psbe psal *psb1 *ycf39 |
| *Porphyra purpyrea* *–* *Galdieria sulphuraria* | psac *psak *psba ycf35 psby_2 rpl32 rpl21 rpl27 rps6 psbd psbc rps16 psbw rps1 *rpl12 *rpl1 *rpl11 *rpoz ycf33 *rpl19 clpc_1 *rpl9 \| rps4 *rpl28 rbcl *psbv *psbx *psaj *psaf *ycf37 *rpl34 psam \| psal *psb1 *ycf39 *rps2 *rpoc2_1 *rpoc1 *rpob *rps20 rpl33 rps18 ycf3 *rpl20 *rpl35 ycf31 psb28 ycf36 psad *psbz psbk *rps14 *psab *psaa rpl3 rpl4 rpl23 rpl2 rps19 rpl22 rps3 rpl16 rpl29 rps17 rpl14 rpl24 rpl5 rps8 rpl6 rpl18 rps5 rpl36 rps13 rps11 rpoa rpl13 rps9 rpl31 rps12 rps7 tufa rps10 ycf38 psbb psbt *psbn psbh *psae psb30 psa1 *psbj *psbl *psbf *psbe |
| *Guillardia theta – Galdieria sulphuraria* | psac *psak *psba rps6 *rpl34 psbd psbc rps16 psbw *rpl12 *rpl1 *rpl11 *rpoz \| rps4 *rpl28 rbcl *psbv *psbx *psaj *psaf *ycf37 psam \| psal *psb1 *ycf39 *rps2 *rpoc2_1 *rpoc1 *rpob *rps20 rpl33 rps18 ycf3 *rpl20 *rpl35 ycf31 ycf36 psad *psbz psbk *rps14 *psab *psaa rpl3 rpl4 rpl23 rpl2 rps19 rpl22 rps3 rpl16 rpl29 rps17 rpl14 rpl24 rpl5 rps8 rpl6 rpl18 rps5 rpl36 rps13 rps11 rpoa rpl13 rps9 rpl31 rps12 rps7 tufa rps10 ycf38 psbb psbt *psbn psbh *psae ycf33 *rpl19 clpc_1 *rpl9 psb30 ycf35 rpl21 rpl27 psby_2 rpl32 psa1 *psbj *psbl *psbf *psbe |
| *Galdieria sulphuraria* (*l*) | psbd psbc rps16 psbw *rpl12 *rpl1 *rpl11 rpl9 *clpc_1 rpl19 psae *psbh psbn *psbt *psbb *ycf38 *rps10 *tufa *rps7 *rps12 *rpl31 *rps9 *rpl13 *rpoa *rps11 *rps13 *rpl36 *rps5 *rpl18 *rpl6 *rps8 *rpl5 *rpl14 *rps17 *rpl29 *rpl16 *rps3 *rpl22 *rps19 *rpl2 *rpl23 *rpl4 *rpl3 psaa psab rps14 *psbk psbz *psad *ycf36 rpl35 rpl20 *ycf3 *rps18 *rpl33 rps20 rpob rpoc1 rpoc2_1 rps2 ycf39 psb1 *psal psbe psbf psbl psbj *psa1 *psb30 ycf37 psaf psaj psbx psbv *rbcl rpl28 *rps4 *psam rpl21 rpl27 psby_2 rpl32 *rpoz psac *psak *psba rps6 |
| *Guillardia theta – Cryptomonas paramecium* | rps4 rbcl *psbv *psbx *psaj *psaf *ycf37 psac *psak *psba rps6 *rpl34 psam \| psal *psb1 *ycf39 *rps2 *rpoc2_1 *rpoc1 *rpob *rps20 rpl33 rps18 ycf3 *rpl20 *rpl35 ycf31 ycf36 psad *psbz psbk *rps14 *psab *psaa rpl3 rpl4 rpl23 rpl2 rps19 rpl22 rps3 rpl16 rpl29 rps17 rpl14 rpl24 rpl5 rps8 rpl6 rpl18 rps5 rpl36 rps13 rps11 rpoa rpl13 rps9 rpl31 rps12 rps7 tufa rps10 ycf38 psbb psbt *psbn psbh *psae ycf33 *rpl19 clpc_1 rpl11 rpl1 rpl12 *psbw *rps16 *psbc *psbd rpl21 rpl27 psby_2 rpl32 *ycf35 psa1 *psbj *psbl *psbf *psbe |
| *Cryptomonas paramecium* (*l*) | rps4 rbcl *rps2 *rpoc2_1 *rpoc1 *rpob *rps20 rpl33 rps18 *rpl20 *rpl35 *rps14 rpl3 rpl4 rpl23 rpl2 rps19 rpl22 rps3 rpl16 rpl29 rps17 rpl14 rpl24 rpl5 rps8 rpl6 rpl18 rps5 rpl36 rps13 rps11 rpoa rpl13 rps9 rpl31 rps12 rps7 tufa rps10 *rpl19 clpc_1 rpl11 rpl1 rpl12 rpl34 rps16 *rpl27 *rpl21 |
| *Guillardia theta – Rhodomonas salina* | psac *psak *psba rps6 psam rps4 rbcl *psbv *psbx *psaj *psaf *ycf37 psa1 *psbj *psbl *psbf *psbe psal *psb1 *ycf39 *rps2 *rpoc2_1 *rpoc1 *rpob *rps20 rpl33 rps18 ycf3 *rpl20 *rpl35 ycf31 ycf36 psad *psbz psbk *rps14 *psab *psaa rpl3 rpl4 rpl23 rpl2 rps19 rpl22 rps3 rpl16 rpl29 rps17 rpl14 rpl24 rpl5 rps8 rpl6 rpl18 rps5 rpl36 rps13 rps11 rpoa rpl13 rps9 rpl31 rps12 rps7 tufa rps10 psbb psbt *psbn psbh *psae ycf33 *rpl19 clpc_1 rpl11 rpl1 rpl12 *psbw *rps16 *psbc *psbd rpl21 rpl27 psby_2 rpl32 *ycf35 *rpl34 |
| *Guillardia theta* (*l*) | *rpl19 clpc_1 rpl11 rpl1 rpl12 *psbw *rps16 *psbc *psbd rpl21 rpl27 psby_2 rpl32 *ycf35 *rpl34 psac *psak *psba rps6 psam rps4 rbcl *psbv *psbx *psaj *psaf *ycf37 psa1 *psbj *psbl *psbf *psbe psal *psb1 *ycf39 *rps2 *rpoc2_1 *rpoc1 *rpob *rps20 rpl33 rps18 ycf3 *rpl20 *rpl35 ycf31 ycf36 psad *psbz psbk *rps14 *psab *psaa rpl3 rpl4 rpl23 rpl2 rps19 rpl22 rps3 rpl16 rpl29 rps17 rpl14 rpl24 rpl5 rps8 rpl6 rpl18 rps5 rpl36 rps13 rps11 rpoa rpl13 rps9 rpl31 rps12 rps7 tufa rps10 psbb psbt *psbn psbh *psae ycf33 |
| *Rhodomonas salina* (*l*) | *rpl19 clpc_1 rpl11 rpl1 rpl12 *psbw *rps16 *psbc *psbd rpl21 rpl27 psby_2 rpl32 *ycf35 *rpl34 psac *psak *psba rps6 *psam rps4 rbcl *psbv *psbx *psaj *psaf *ycf37 psa1 *psbj *psbl *psbf *psbe psal *psb1 *ycf39 *rps2 *rpoc2_1 *rpoc1 *rpob *rps20 rpl33 rps18 ycf3 *rpl20 *rpl35 ycf36 psad *psbz psbk *rps14 *psab *psaa rpl3 rpl4 rpl23 rpl2 rps19 rpl22 rps3 rpl16 rpl29 rps17 rpl14 rpl24 rpl5 rps8 rpl6 rpl18 rps5 rpl36 rps13 rps11 rpoa rpl13 rps9 rpl31 rps12 rps7 tufa rps10 psbb psbt *psbn psbh *psae ycf33 |
| *Porphyra purpyrea* *–* *Pyropia yezoensis* | psac *psak *rps4 psal *rpl28 rbcl *psb1 *ycf39 *rps2 *rpoc2_1 *rpoc1 *rpob *rps20 rpl33 rps18 ycf3 *rpl20 *rpl35 ycf36 psad psaa psab rps14 *psbk psbz rpl3 rpl4 rpl23 rpl2 rps19 rpl22 rps3 rpl16 rpl29 rps17 rpl14 rpl24 rpl5 rps8 rpl6 rpl18 rps5 rpl36 rps13 rps11 rpoa rpl13 rps9 rpl31 rps12 rps7 tufa rps10 ycf38 psbb psbt *psbn psbh *psae psb28 psb30 psa1 *psbj *psbl *psbf *psbe *psam *rpl34 ycf37 psaf psaj psbx psbv psby_2 rpl32 rpl21 rpl27 *psba ycf35 rps6 psbd psbc rps16 psbw rps1 *rpl12 *rpl1 *rpl11 rpl9 *clpc_1 rpl19 *ycf33 *rpoz |
| *Pyropia yezoensis* (*l*) | *rpl34 ycf37 psaf psaj psbx psbv rpl32 rpl21 rpl27 *psba ycf35 *rps4 psal *rpl28 rbcl *psb1 *ycf39 *rps2 *rpoc2_1 *rpoc1 *rpob *rps20 rpl33 rps18 ycf3 *rpl20 *rpl35 psad psaa psab *rps10 *tufa *rps7 *rps12 rpl31 *rps9 *rpl13 *rpoa rps11 rps13 *rpl36 *rps5 *rpl18 *rpl6 *rps8 *rpl5 *rpl24 *rpl14 *rps17 *rpl29 *rpl16 *rps3 *rpl22 *rps19 *rpl2 *rpl23 *rpl4 *rpl3 *psbz psbk *rps14 psbb psbt *psbn psbh *psae *rpl19 clpc_1 *rpl9 rpl11 rpl1 rpl12 *rps1 *psbw *rps16 *psbc *psbd *rps6 psak *psac psa1 *psbj *psbl *psbf *psbe *psam |
| *Porphyra purpyrea* *– Calliarthron tuberculosum* | rpoz psb28 psb30 psa1 *psbj *psbl *psbf *psbe *psam *rpl34 ycf37 psaf psaj psbx psbv psby_2 rpl32 rpl21 rpl27 *psba ycf35 rps6 psbd psbc rps16 psbw rps1 *rpl12 *rpl1 *rpl11 rpl9 *clpc_1 rpl19 *ycf33 psae *psbh psbn *psbt *psbb *ycf38 *rps10 *tufa *rps7 *rps12 *rpl31 *rps9 *rpl13 *rpoa *rps11 *rps13 *rpl36 *rps5 *rpl18 *rpl6 *rps8 *rpl5 *rpl24 *rpl14 *rps17 *rpl29 *rpl16 *rps3 *rpl22 *rps19 *rpl2 *rpl23 *rpl4 *rpl3 *psbz psbk *rps14 *psab *psaa *psad ycf36 rpl35 rpl20 *ycf3 *rps18 *rpl33 rps20 rpob rpoc1 rpoc2_1 rps2 ycf39 psb1 psal *rpl28 rbcl ycf34 rps4 psak *psac |
| *Porphyra purpyrea* *–* *Pyropia perforata* | psam psbe psbf psbl psbj *psa1 psac *psak rps6 psbd psbc rps16 psbw rps1 *rpl12 *rpl1 *rpl11 rpl9 *clpc_1 rpl19 *ycf33 psae *psbh psbn *psbt *psbb *ycf38 rps14 *psbk psbz rpl3 rpl4 rpl23 rpl2 rps19 rpl22 rps3 rpl16 rpl29 rps17 rpl14 rpl24 rpl5 rps8 rpl6 rpl18 rps5 rpl36 rps13 rps11 rpoa rpl13 rps9 rpl31 rps12 rps7 tufa rps10 *psab *psaa *psad ycf31 ycf36 rpl35 rpl20 *ycf3 *rps18 *rpl33 rps20 rpob rpoc1 rpoc2_1 rps2 ycf39 psb1 *ycf34 *rbcl rpl28 *psal rps4 *ycf35 psba *rpl27 *rpl21 *rpl32 *psby_2 *psbv *psbx *psaj *psaf *ycf37 rpl34 |
| *Porphyra purpyrea* *–* *Pyropia haitanensis* | *rpl34 ycf37 psaf psaj psbx psbv psby_2 rpl32 rpl21 rpl27 *psba ycf35 *rps4 psal *rpl28 rbcl ycf34 *psb1 *ycf39 *rps2 *rpoc2_1 *rpoc1 *rpob *rps20 rpl33 rps18 ycf3 *rpl20 *rpl35 *ycf36 *ycf31 psad psaa psab *rps10 *tufa *rps7 *rps12 *rpl31 *rps9 *rpl13 *rpoa *rps11 *rps13 *rpl36 *rps5 *rpl18 *rpl6 *rps8 *rpl5 *rpl24 *rpl14 *rps17 *rpl29 *rpl16 *rps3 *rpl22 *rps19 *rpl2 *rpl23 *rpl4 *rpl3 *psbz psbk *rps14 ycf38 psbb psbt *psbn psbh *psae ycf33 *rpl19 clpc_1 *rpl9 rpl11 rpl1 rpl12 *rps1 *psbw *rps16 *psbc *psbd *rps6 psak *psac psa1 *psbj *psbl *psbf *psbe *psam |
| *Porphyra purpyrea* (*l*) | *rpl34 ycf37 psaf psaj psbx psbv psby_2 rpl32 rpl21 rpl27 *psba ycf35 *rps4 psal *rpl28 rbcl ycf34 *psb1 *ycf39 *rps2 *rpoc2_1 *rpoc1 *rpob *rps20 rpl33 rps18 ycf3 *rpl20 *rpl35 *ycf36 *ycf31 psad psaa psab *rps10 *tufa *rps7 *rps12 *rpl31 *rps9 *rpl13 *rpoa *rps11 *rps13 *rpl36 *rps5 *rpl18 *rpl6 *rps8 *rpl5 *rpl24 *rpl14 *rps17 *rpl29 *rpl16 *rps3 *rpl22 *rps19 *rpl2 *rpl23 *rpl4 *rpl3 *psbz psbk *rps14 ycf38 psbb psbt *psbn psbh *psae ycf33 *rpl19 clpc_1 *rpl9 rpl11 rpl1 rpl12 *rps1 *psbw *rps16 *psbc *psbd *rps6 psak *psac psa1 *psbj *psbl *psbf *psbe *psam |
| *Pyropia haitanensis* (*l*) | *rpl34 ycf37 psaf psaj psbx psbv psby_2 rpl32 rpl21 rpl27 *psba ycf35 *rps4 psal *rpl28 rbcl ycf34 *psb1 *ycf39 *rps2 *rpoc2_1 *rpoc1 *rpob *rps20 rpl33 rps18 ycf3 *rpl20 *rpl35 *ycf36 *ycf31 psad psaa psab *rps10 *tufa *rps7 *rps12 *rpl31 *rps9 *rpl13 *rpoa *rps11 *rps13 *rpl36 *rps5 *rpl18 *rpl6 *rps8 *rpl5 *rpl24 *rpl14 *rps17 *rpl29 *rpl16 *rps3 *rpl22 *rps19 *rpl2 *rpl23 *rpl4 *rpl3 *psbz psbk *rps14 ycf38 psbb psbt *psbn psbh *psae ycf33 *rpl19 clpc_1 *rpl9 rpl11 rpl1 rpl12 *rps1 *psbw *rps16 *psbc *psbd *rps6 psak *psac psa1 *psbj *psbl *psbf *psbe *psam |
| *Pyropia perforata* (*l*) | *rpl34 ycf37 psaf psaj psbx psbv psby_2 rpl32 rpl21 rpl27 *psba ycf35 *rps4 psal *rpl28 rbcl ycf34 *psb1 *ycf39 *rps2 *rpoc2_1 *rpoc1 *rpob *rps20 rpl33 rps18 ycf3 *rpl20 *rpl35 *ycf36 psad psaa psab *rps10 *tufa *rps7 *rps12 *rpl31 *rps9 *rpl13 *rpoa *rps11 *rps13 *rpl36 *rps5 *rpl18 *rpl6 *rps8 *rpl5 *rpl24 *rpl14 *rps17 *rpl29 *rpl16 *rps3 *rpl22 *rps19 *rpl2 *rpl23 *rpl3 *rpl4 *psbz psbk *rps14 ycf38 psbt *psbn psbh *psae *ycf33 *rpl19 clpc_1 *rpl9 rpl11 rpl1 rpl12 *rps1 *psbw *rps16 *psbc *psbd *rps6 psak *psac psa1 *psbj *psbl *psbf *psbe *psam |
| *Gracilaria tenuistipilata – Calliarthron tuberculosum* | rpoz psb28 psb30 psa1 *psbj *psbl *psbf *psbe *psam *rpl34 ycf37 psaf psaj psbx psbv psby_2 rpl32 rpl21 rpl27 *psba ycf35 rps6 psbd psbc rps16 psbw rps1 *rpl12 *rpl1 *rpl11 rpl9 *clpc_1 rpl19 *ycf33 psae *psbh psbn *psbt *psbb *ycf38 *rps10 *tufa *rps7 *rps12 *rpl31 *rps9 *rpl13 *rpoa *rps11 *rps13 *rpl36 *rps5 *rpl18 *rpl6 *rps8 *rpl5 *rpl24 *rpl14 *rps17 *rpl29 *rpl16 *rps3 *rpl22 *rps19 *rpl2 *rpl23 *rpl4 *rpl3 *psbz psbk *rps14 *psab *psaa *psad ycf36 rpl35 rpl20 *ycf3 *rps18 *rpl33 rps20 rpob rpoc1 rpoc2_1 rps2 ycf39 psb1 psal *rpl28 rbcl ycf34 rps4 psak *psac |
| *Gracilaria tenuistipilata – Gracilaria Salicornia* | psb30 psa1 *psbj *psbl *psbf *psbe *psam *rpl34 ycf37 psaf psaj psbx psbv psby_2 rpl32 rpl21 rpl27 *psba ycf35 rps6 psbd psbc rps16 *rpl9 rpl11 rpl1 rpl12 *rps1 *psbw clpc_1 rpl19 *ycf33 psae *psbh psbn *psbt *psbb *ycf38 *rps10 *tufa *rps7 *rps12 *rpl31 *rps9 *rpl13 *rpoa *rps11 *rps13 *rpl36 *rps5 *rpl18 *rpl6 *rps8 *rpl5 *rpl24 *rpl14 *rps17 *rpl29 *rpl16 *rps3 *rpl22 *rps19 *rpl2 *rpl23 *rpl4 *rpl3 *psbz psbk *rps14 *psab *psaa *psad ycf36 rpl35 rpl20 *ycf3 *rps18 *rpl33 rps20 rpob rpoc1 rpoc2_1 rps2 ycf39 psb1 psal *rpl28 rbcl ycf34 rps4 psak *psac |
| *Gracilaria tenuistipilata* (*l*) | *rpl34 ycf37 psaf psaj psbx psbv psby_2 rpl32 rpl21 rpl27 *psba ycf35 rps6 psbd psbc rps16 *rpl9 rpl11 rpl1 rpl12 *rps1 *psbw clpc_1 rpl19 *ycf33 psae *psbh psbn *psbt *psbb *ycf38 *rps10 *tufa *rps7 *rps12 *rpl31 *rps9 *rpl13 *rpoa *rps11 *rps13 *rpl36 *rps5 *rpl18 *rpl6 *rps8 *rpl5 *rpl24 *rpl14 *rps17 *rpl29 *rpl16 *rps3 *rpl22 *rps19 *rpl2 *rpl23 *rpl4 *rpl3 psbk *rps14 *psab *psaa *psad ycf36 rpl35 rpl20 *ycf3 *rps18 *rpl33 rps20 rpob rpoc1 rpoc2_1 rps2 ycf39 psb1 psal *rpl28 rbcl ycf34 rps4 psak *psac psa1 *psbj *psbl *psbf *psbe *psam |
| *Gracilaria salicornia* (*l*) | *rpl34 ycf37 psaf psaj psbx psbv psby_2 rpl32 rpl21 rpl27 *psba ycf35 rps6 psbd psbc rps16 *rpl9 rpl11 rpl1 rpl12 *rps1 *psbw clpc_1 rpl19 *ycf33 psae *psbh psbn *psbt *psbb *ycf38 *rps10 *tufa *rps7 *rps12 *rpl31 *rps9 *rpl13 *rpoa *rps11 *rps13 *rpl36 *rps5 *rpl18 *rpl6 *rps8 *rpl5 *rpl24 *rpl14 *rps17 *rpl29 *rpl16 *rps3 *rpl22 *rps19 *rpl2 *rpl23 *rpl4 *rpl3 *psbz psbk *rps14 *psab *psaa *psad ycf36 rpl35 rpl20 *ycf3 *rps18 *rpl33 rps20 rpob rpoc1 rpoc2_1 rps2 ycf39 psb1 psal *rpl28 rbcl ycf34 rps4 psak *psac psb30 psa1 *psbj *psbl *psbf *psbe *psam |
| *Chondrus crispus – Calliarthron tuberculosum* | rpoz psb30 psa1 *psbj *psbl *psbf *psbe *psam *rps6 *ycf35 psba *rpl27 *rpl21 *rpl32 *psby_2 *psbv *psbx *psaj *psaf *ycf37 rpl34 psbd psbc rps16 psb28 rps1 *rpl12 *rpl1 *rpl11 rpl9 *clpc_1 rpl19 *ycf33 psae *psbh psbn *psbt *psbb *ycf38 *rps10 *tufa *rps7 *rps12 *rpl31 *rps9 *rpl13 *rpoa *rps11 *rps13 *rpl36 *rps5 *rpl18 *rpl6 *rps8 *rpl5 *rpl24 *rpl14 *rps17 *rpl29 *rpl16 *rps3 *rpl22 *rps19 *rpl2 *rpl23 *rpl4 *rpl3 *psbz psbk *rps14 *psab *psaa *psad ycf36 rpl35 rpl20 *ycf3 *rps18 *rpl33 rps20 rpob rpoc1 rpoc2_1 rps2 ycf39 psb1 psal *rpl28 rbcl ycf34 rps4 psak *psac |
| *Calliarthron tuberculosum* (*l*) | *rps6 psba *rpl27 *rpl21 *rpl32 *psby_2 *psbv *psbx *psaj *psaf *ycf37 rpl34 psbd psbc rps16 psb28 rps1 *rpl12 *rpl1 *rpl11 rpl9 *clpc_1 rpl19 *ycf33 psae *psbh psbn *psbt *psbb *ycf38 *rps10 *tufa *rps7 *rps12 *rpl31 *rps9 *rpl13 *rpoa *rps11 *rps13 *rpl36 *rps5 *rpl18 *rpl6 *rps8 *rpl5 *rpl24 *rpl14 *rps17 *rpl29 *rpl16 *rps3 *rpl22 *rps19 *rpl2 *rpl23 *rpl4 *rpl3 *psbz psbk *rps14 *psab *psaa *psad ycf36 rpl35 rpl20 *ycf3 *rps18 *rpl33 rps20 rpob rpoc1 rpoc2_1 rps2 ycf39 psb1 psal *rpl28 rbcl rps4 psak *psac rpoz psb30 psa1 *psbj *psbl *psbf *psbe *psam |
| *Chondrus crispus – Vertebrata lanosa* | rpoz psb30 psa1 *psbj *psbl *psbf *psbe *psam *rps6 *ycf35 psba *rpl27 *rpl21 *rpl32 *psby_2 *psbv *psbx *psaj *psaf *ycf37 rpl34 psbd psbc rps16 psb28 rps1 *rpl12 *rpl1 *rpl11 rpl9 *clpc_1 rpl19 *ycf33 psae *psbh psbn *psbt *psbb *ycf38 *rps10 *tufa *rps7 *rps12 *rpl31 *rps9 *rpl13 *rpoa *rps11 *rps13 *rpl36 *rps5 *rpl18 *rpl6 *rps8 *rpl5 *rpl24 *rpl14 *rps17 *rpl29 *rpl16 *rps3 *rpl22 *rps19 *rpl2 *rpl23 *rpl4 *rpl3 *psbz psbk *rps14 *psab *psaa *psad ycf36 rpl35 rpl20 *ycf3 *rps18 *rpl33 rps20 rpob rpoc1 rpoc2_1 rps2 ycf39 psb1 psal *rpl28 rbcl ycf34 rps4 psak *psac |
| *Vertebrata lanosa* (*l*) | rpl9 *clpc_1 rpl19 *ycf33 psae *psbh psbn *psbt *psbb *ycf38 *rps10 *tufa *rps7 *rps12 *rpl31 *rps9 *rpl13 *rpoa *rps11 *rps13 *rpl36 *rps5 *rpl18 *rpl6 *rps8 *rpl5 rpl24 *rpl14 *rps17 *rpl29 *rpl16 *rps3 *rpl22 *rps19 *rpl2 *rpl23 *rpl4 *rpl3 *psbz psbk *rps14 *psab *psaa *psad ycf36 rpl35 rpl20 *ycf3 *rps18 *rpl33 rps20 rpob rpoc1 rpoc2_1 rps2 ycf39 psb1 psal *rpl28 rbcl ycf34 rps4 psak *psac rpoz psb30 psa1 *psbj *psbl *psbf *psbe *psam *rps6 *ycf35 psba *rpl27 *rpl21 *rpl32 *psby_2 *psbv *psbx *psaj *psaf rpl34 psbd psbc rps16 psb28 rps1 *rpl12 *rpl1 *rpl11 |
| *Chondrus crispus – Grateloupia taiwanensis* | rpoz psb30 psa1 *psbj *psbl *psbf *psbe *psam *rps6 *ycf35 psba *rpl27 *rpl21 *rpl32 *psby_2 *psbv *psbx *psaj *psaf *ycf37 rpl34 psbd psbc rps16 psb28 rps1 *rpl12 *rpl1 *rpl11 rpl9 *clpc_1 rpl19 *ycf33 psae *psbh psbn *psbt *psbb *ycf38 *rps10 *tufa *rps7 *rps12 *rpl31 *rps9 *rpl13 *rpoa *rps11 *rps13 *rpl36 *rps5 *rpl18 *rpl6 *rps8 *rpl5 *rpl24 *rpl14 *rps17 *rpl29 *rpl16 *rps3 *rpl22 *rps19 *rpl2 *rpl23 *rpl4 *rpl3 *psbz psbk *rps14 *psab *psaa *psad ycf36 rpl35 rpl20 *ycf3 *rps18 *rpl33 rps20 rpob rpoc1 rpoc2_1 rps2 ycf39 psb1 psal *rpl28 rbcl ycf34 rps4 psak *psac |
| *Chondrus crispus* (*l*) | *rpl34 ycf37 psaf psaj psbx psbv psby_2 rpl32 rpl21 rpl27 *psba ycf35 rps6 psbd psbc rps16 psb28 rps1 *rpl12 *rpl1 *rpl11 rpl9 *clpc_1 rpl19 *ycf33 psae *psbh psbn *psbt *psbb *ycf38 *rps10 *tufa *rps7 *rps12 *rpl31 *rps9 *rpl13 *rpoa *rps11 *rps13 *rpl36 *rps5 *rpl18 *rpl6 *rps8 *rpl5 *rpl24 *rpl14 *rps17 *rpl29 *rpl16 *rps3 *rpl22 *rps19 *rpl2 *rpl23 *rpl4 *rpl3 *psbz psbk *rps14 *psab *psaa *psad ycf36 rpl35 rpl20 *ycf3 *rps18 *rpl33 rps20 rpob rpoc1 rpoc2_1 rps2 ycf39 psb1 psal *rpl28 rbcl ycf34 rps4 psak *psac rpoz psb30 psa1 *psbj *psbl *psbf *psbe *psam |
| *Grateloupia taiwanensis* (*l*) | rbcl ycf34 rps4 psak *psac rpoz psa1 *psbj *psbl *psbf *psbe *psam *rps6 *ycf35 psba *rpl27 *rpl21 *rpl32 *psby_2 *psbv *psbx *psaj *psaf ycf37 rpl34 psbd psbc rps16 psb28 rps1 *rpl12 *rpl1 *rpl11 rpl9 clpc_1 rpl19 *ycf33 psae *psbh psbn *psbt *psbb *ycf38 *rps10 *tufa *rps7 *rps12 *rpl31 *rps9 *rpl13 *rpoa *rps11 *rps13 *rpl36 *rps5 *rpl18 *rpl6 *rps8 *rpl5 *rpl24 *rpl14 *rps17 *rpl29 *rpl16 *rps3 *rpl22 *rps19 *rpl2 *rpl23 *rpl4 *rpl3 *psbz psbk *rps14 *psab *psaa *psad ycf36 rpl35 rpl20 *ycf3 *rps18 *rpl33 rps20 rpob rpoc1 rpoc2_1 rps2 ycf39 psb1 psal *rpl28 |
| *Toxoplasma gondii – Vaucheria litorea* | psby_1 rpoc1 rbcl29 psbv_1 psac_2 psaj_2 \| psam rpl33 rps18 ycf3 *rps2 *rpoc2_1 *rpoc1 *rpob *rps20 psaf psaj_1 psbd psbc rps16 rps4 ycf33 *rps1 *psb28 rpl9 rpl11 ycf36 psad psb1 psba *rbcl *psby_2 *rps6 *rpl34 rpl21 rpl27 *psac_1 rpl32 psal psbk *rpl20 *rpl35 ycf37 *ycf35 *tufa *rps12 *rpoa *rps19 *rpl2 *rpl3 *rps14 rpl23 rpl22 rps3 rpl16 rps17 rpl14 rpl5 rps8 rpl6 rps5 psbb psbt *psbn psbh *psbv_2 *clpc_1 *psbz psbm *psb30 psbe psbf psbl psbj *rpl4 *rps7 *rpl31 *rps9 *rps11 *rps13 *rpl36 *psa1 psaa psab \| psbx rps10 *ycf39 rpoz *rpl12 *rpl1 rpl19 |
| *Vaucheria litorea* (*l*) | psam rpl33 rps18 ycf3 *rps2 *rpoc2_1 *rpoc1 *rpob *rps20 psaf psaj_1 psbd psbc rps16 rps4 ycf33 *rps1 *psb28 rpl9 rpl11 rpl1 rpl12 *psae psbb psbt *psbn psbh *rps10 *tufa *rps7 *rps12 *rpl31 *rps9 *rpl13 *rpoa *rps11 *rps13 *rpl36 *rps5 *rpl18 *rpl6 *rps8 *rpl5 *rpl24 *rpl14 *rps17 *rpl29 *rpl16 *rps3 *rpl22 *rps19 *rpl2 *rpl23 *rpl4 *rpl3 *rpl20 *rpl35 *ycf39 *ycf37 *rbcl *psba psb1 *rpl19 clpc_1 *psbv_2 *psbx *psal psbe psbf psbl psbj *psa1 *psbk psbz psac_1 *psad *ycf36 *rpl34 *rpl32 *psby_2 *rpl27 *rpl21 psaa psab rps14 |
| *Toxoplasma gondii – Nannochloropsis gaditana* | psby_1 rpoc1 rbcl29 psbv_1 psac_2 psaj_2 \| ycf36 psad psb1 psba *rbcl *ycf3 *rps18 *rpl33 rps20 rpob rpoc1 rpoc2_1 rps2 *psby_2 *rps6 *rpl34 rpl21 rpl27 *psaj_1 *psac_1 rpl32 psaf psal psbk *rpl20 *rpl35 ycf37 *ycf35 rps16 *psbc *psbd *tufa *rps12 *rpoa *rps19 *rpl2 *rpl3 *rps14 rpl23 rpl22 rps3 rpl16 rps17 rpl14 rpl5 rps8 rpl6 rps5 psbb psbt *psbn psbh rps4 *psbv_2 *clpc_1 *psbz psam psbm *psb30 psbe psbf psbl psbj rpl11 *rpl4 *rps7 *rpl31 *rps9 *rps11 *rps13 *rpl36 *psa1 psaa psab \| psbx rps10 *ycf39 rpoz *rpl12 *rpl1 rpl19 |
| *Babesia bovis – Nannochloropsis gaditana* | psby_1 rpoc1 rbcl29 psbv_1 psac_2 psaj_2 \| ycf36 psad psb1 psba *rbcl *ycf3 *rps18 *rpl33 rps20 rpob rpoc1 rpoc2_1 rps2 *psby_2 *clpc_1 *rps6 *rpl34 rpl21 rpl27 *psaj_1 *psac_1 rpl32 psaf psbx *psbc *psbd *ycf34 rpl3 rpl4 rpl23 rpl2 rps19 rpl22 rps3 rpl16 rpl29 rps17 rpl14 rpl5 rps8 rpl6 rpl18 rps5 rpl36 rps13 rps11 rpoa rpl13 rps9 rpl31 rps12 rps7 tufa rps10 psbb psbt *psbn psbh *psae *rpl12 *rpl1 *rpl11 *psbz psbk *rps14 *psab *psaa *psbv_2 *clpc_3 *rpl19_2 *rpl20 *rpl35 *psal psbe psbf psbl psbj *psa1 psbw *rps4 rps16 |
| *Nannochloropsis gaditana* (*l*) | clpc_3 psbv_2 psaa psab *psbk rpl11 rpl1 rpl12 psae *psbh psbn *psbt *rps10 *tufa *rps7 *rps12 *rpl31 *rps9 *rpl13 *rpoa *rps11 *rps13 *rpl36 *rps5 *rpl18 *rpl6 *rps8 *rpl5 *rpl14 *rps17 *rbcl29 *rpl16 *rps3 *rpl22 *rps19 *rpl2 *rpl23 *rpl4 *rpl3 psbd *psbx *psaf *rpl32 *rpl19_2 *rpl20 *rpl35 *psal psbe psbf psbj *rps4 rps16 ycf36 psad psb1 psba *rbcl *ycf3 *rps18 *rpl33 rps20 rpob rpoc1 rpoc2_1 rps2 *psby_1 *clpc_1 *rps6 *rpl34 rpl21 rpl27 *psaj_1 *psac_1 |
| *Babesia bovis – Nannochloropsis limnetica* | ycf36 psad psb1 psba *rbcl *ycf3 *rps18 *rpl33 rps20 rpob rpoc1 rpoc2_1 rps2 *psby_2 *clpc_1 *rps6 *rpl34 rpl21 rpl27 *psaj_1 *psac_1 clpc_2 psbv_1 psac_2 psaj_2 rpl32 psaf psbx *psbc *psbd *ycf34 rpl3 rpl4 rpl23 rpl2 rps19 rpl22 rps3 rpl16 rpl29 rps17 rpl14 rpl5 rps8 rpl6 rpl18 rps5 rpl36 rps13 rps11 rpoa rpl13 rps9 rpl31 rps12 rps7 tufa rps10 psbb psbt *psbn psbh *psae *rpl12 *rpl1 *rpl11 *psbz psbk *rps14 *psab *psaa *psbv_2 *clpc_3 *rpl19_2 *rpl20 *rpl35 *psal psbe psbf psbl psbj *psa1 psbw *rps4 rps16 |
| *Babesia bovis – Trachydiscus minutus* | psbh *psae *rpl12 *rpl1 *rpl11 psbv_1 *psbz psbk *rps14 *psab *psaa *psbv_2 rpl3 rpl4 rpl23 rpl2 rps19 rpl22 rps3 rpl16 rpl29 rps17 rpl14 rpl5 rps8 rpl6 rpl18 rps5 rpl36 rps13 rps11 rpoa rpl13 rps9 rpl31 rps12 rps7 tufa rps10 psbb psbt *psbn \| clpc_3 ycf34 psbd psbc *psbx *psaf *rpl32 *clpc_2 psac_1 psaj_1 *rpl19_1 *rpl27 *rpl21 rpl34 rps6 clpc_1 psby_2 *rbcl *ycf3 *rps18 *rpl33 rps20 rpob rpoc1 rpoc2_1 rps2 *psba *psb1 *psad *rps16 rps4 *psbw psa1 *psbj *psbl *psbf *psbe psal rpl35 rpl20 rpl19_2 *psaj_2 *psac_2 |
| *Babesia bovis* (*l*) | *rps2 *rpoc2_1 *rpoc1 *rpob *clpc_2 *clpc_1 *tufa *rps12 *rps11 *rps13 *rps8 *rpl14 *rpl16 *rps3 *rpl2 |
| *Trachydiscus minutus* (*l*) | *psae *rpl12 *rpl1 *rpl11 ycf34 psbd psbc psam *psbx *psaf *rpl32 *psbz psbk *rps14 *psab *psaa *psbv_2 *clpc_2 psac_1 psaj_1 *rpl19_1 *rpl27 *rpl21 rpl34 rps6 clpc_1 psby_2 *rbcl *rps2 *rpoc2_1 *rpoc1 *rpob *rps20 rpl33 rps18 ycf3 *psba *psb1 *psad *rps16 rps4 *psbw psa1 *psbj *psbl *psbf *psbe psal rpl35 rpl20 rpl19_2 *psaj_2 *psac_2 clpc_3 psbv_1 rpl3 rpl4 rpl23 rpl2 rps19 rpl22 rps3 rpl16 rpl29 rps17 rpl14 rpl5 rps8 rpl6 rpl18 rps5 rpl36 rps13 rps11 rpoa rpl13 rps9 rpl31 rps12 rps7 tufa rps10 psbb psbt *psbn psbh |
| *Nannochloropsis granulata – Nannochloropsis limnetica* | ycf36 psad psb1 psba *rbcl *ycf3 *rps18 *rpl33 rps20 rpob rpoc1 rpoc2_1 rps2 *psby_2 *clpc_1 *rps6 *rpl34 rpl21 rpl27 *psaj_1 *psac_1 clpc_2 psbv_1 psac_2 psaj_2 rpl32 psaf psbx *psbc *psbd *ycf34 rpl3 rpl4 rpl23 rpl2 rps19 rpl22 rps3 rpl16 rpl29 rps17 rpl14 rpl5 rps8 rpl6 rpl18 rps5 rpl36 rps13 rps11 rpoa rpl13 rps9 rpl31 rps12 rps7 tufa rps10 psbb psbt *psbn psbh *psae *rpl12 *rpl1 *rpl11 *psbz psbk *rps14 *psab *psaa *psbv_2 *clpc_3 *rpl19_2 *rpl20 *rpl35 *psal psbe psbf psbl psbj *psa1 psbw *rps4 rps16 |
| *Nannochloropsis granulata – Nannochloropsis oceanica* | psaa psab rps14 *psbk psbz rpl11 rpl1 rpl12 psae *psbh psbn *psbt *psbb *rps10 *tufa *rps7 *rps12 *rpl31 *rps9 *rpl13 *rpoa *rps11 *rps13 *rpl36 *rps5 *rpl18 *rpl6 *rps8 *rpl5 *rpl14 *rps17 *rpl29 *rpl16 *rps3 *rpl22 *rps19 *rpl2 *rpl23 *rpl4 *rpl3 ycf34 psbd psbc *psbx *psaf *rpl32 *psbv_1 *clpc_2 psac_1 psaj_1 *rpl27 *rpl21 rpl34 rps6 clpc_1 psby_2 *rps2 *rpoc2_1 *rpoc1 *rpob *rps20 rpl33 rps18 ycf3 rbcl *psba *psb1 *psad *ycf36 *rps16 rps4 *psbw psa1 *psbj *psbl *psbf *psbe psal rpl35 rpl20 rpl19_2 clpc_3 psbv_2 |
| *Nannochloropsis granulata – Nannochloropsis oculata* | psaa psab rps14 *psbk psbz rpl11 rpl1 rpl12 psae *psbh psbn *psbt *psbb *rps10 *tufa *rps7 *rps12 *rpl31 *rps9 *rpl13 *rpoa *rps11 *rps13 *rpl36 *rps5 *rpl18 *rpl6 *rps8 *rpl5 *rpl14 *rps17 *rpl29 *rpl16 *rps3 *rpl22 *rps19 *rpl2 *rpl23 *rpl4 *rpl3 ycf34 psbd psbc *psbx *psaf *rpl32 *psbv_1 *clpc_2 psac_1 psaj_1 *rpl27 *rpl21 rpl34 rps6 clpc_1 psby_2 *rps2 *rpoc2_1 *rpoc1 *rpob *rps20 rpl33 rps18 ycf3 rbcl *psba *psb1 *psad *ycf36 *rps16 rps4 *psbw psa1 *psbj *psbl *psbf *psbe psal rpl35 rpl20 rpl19_2 clpc_3 psbv_2 |
| *Nannochloropsis granulate* (*l*) | psaa psab rps14 *psbk psbz rpl11 rpl1 rpl12 psae *psbh psbn *psbt *psbb *rps10 *tufa *rps7 *rps12 *rpl31 *rps9 *rpl13 *rpoa *rps11 *rps13 *rpl36 *rps5 *rpl18 *rpl6 *rps8 *rpl5 *rpl14 *rps17 *rpl29 *rpl16 *rps3 *rpl22 *rps19 *rpl2 *rpl23 *rpl4 *rpl3 ycf34 psbd psbc *psbx *psaf *rpl32 *psbv_1 *clpc_2 psac_1 psaj_1 *rpl27 *rpl21 rpl34 rps6 clpc_1 psby_2 *rps2 *rpoc2_1 *rpoc1 *rpob *rps20 rpl33 rps18 ycf3 rbcl *psba *psb1 *psad *ycf36 *rps16 rps4 *psbw psa1 *psbj *psbl *psbf *psbe psal rpl35 rpl20 rpl19_2 clpc_3 psbv_2 |
| *Nannochloropsis oculata* (*l*) | psaa psab rps14 *psbk psbz rpl11 rpl1 rpl12 psae *psbh psbn *psbt *psbb *rps10 *tufa *rps7 *rps12 *rpl31 *rps9 *rpl13 *rpoa *rps11 *rps13 *rpl36 *rps5 *rpl18 *rpl6 *rps8 *rpl5 *rpl14 *rps17 *rpl29 *rpl16 *rps3 *rpl22 *rps19 *rpl2 *rpl23 *rpl4 *rpl3 ycf34 psbd psbc *psbx *psaf *rpl32 *psbv_1 *clpc_2 psac_1 psaj_1 *rpl27 *rpl21 rpl34 rps6 clpc_1 psby_2 *rps2 *rpoc2_1 *rpoc1 *rpob *rps20 rpl33 rps18 ycf3 rbcl *psba *psb1 *psad *ycf36 *rps16 rps4 *psbw psa1 *psbj *psbl *psbf *psbe psal rpl35 rpl20 rpl19_2 clpc_3 psbv_2 |
| *Nannochloropsis oceanica* (*l*) | psaa psab rps14 *psbk psbz rpl11 rpl1 rpl12 psae *psbh psbn *psbt *psbb *rps10 *tufa *rps7 *rps12 *rpl31 *rps9 *rpl13 *rpoa *rps11 *rps13 *rpl36 *rps5 *rpl18 *rpl6 *rps8 *rpl5 *rpl14 *rps17 *rpl29 *rpl16 *rps3 *rpl22 *rps19 *rpl2 *rpl23 *rpl4 *rpl3 ycf34 psbd psbc *psbx *psaf *rpl32 *psbv_1 *clpc_2 psac_1 psaj_1 *rpl27 *rpl21 rpl34 rps6 clpc_1 psby_2 *rps2 *rpoc2_1 *rpoc1 *rpob *rps20 rpl33 rps18 ycf3 rbcl *psba *psb1 *psad *ycf36 *rps16 rps4 *psbw psa1 *psbj *psbl *psbf *psbe psal rpl35 rpl20 rpl19_2 clpc_3 psbv_2 |
| *Nannochloropsis salina – Nannochloropsis limnetica* | ycf36 psad psb1 psba *rbcl *ycf3 *rps18 *rpl33 rps20 rpob rpoc1 rpoc2_1 rps2 *psby_2 *clpc_1 *rps6 *rpl34 rpl21 rpl27 *psaj_1 *psac_1 clpc_2 rpl32 psaf psbx *psbc *psbd *ycf34 rpl3 rpl4 rpl23 rpl2 rps19 rpl22 rps3 rpl16 rpl29 rps17 rpl14 rpl5 rps8 rpl6 rpl18 rps5 rpl36 rps13 rps11 rpoa rpl13 rps9 rpl31 rps12 rps7 tufa rps10 psbb psbt *psbn psbh *psae *rpl12 *rpl1 *rpl11 *psbz psbk *rps14 *psab *psaa *psbv_2 *clpc_3 *rpl19_2 *rpl20 *rpl35 *psal psbe psbf psbl psbj *psa1 psbw *rps4 rps16 |
| *Nannochloropsis salina* (*l*) | psaa psab rps14 *psbk psbz rpl11 rpl1 rpl12 psae *psbh psbn *psbt *psbb *rps10 *tufa *rps7 *rps12 *rpl31 *rps9 *rpl13 *rpoa *rps11 *rps13 *rpl36 *rps5 *rpl18 *rpl6 *rps8 *rpl5 *rpl14 *rps17 *rpl29 *rpl16 *rps3 *rpl22 *rps19 *rpl2 *rpl23 *rpl4 *rpl3 ycf34 psbd psbc *psbx *psaf *rpl32 psac_1 psaj_1 *rpl27 *rpl21 rpl34 rps6 clpc_1 psby_2 *rps2 *rpoc2_1 *rpoc1 *rpob *rps20 rpl33 rps18 ycf3 rbcl *psba *psb1 *psad *ycf36 *rps16 rps4 *psbw psa1 *psbj *psbl *psbf *psbe psal rpl35 rpl20 rpl19_2 clpc_3 psbv_2 |
| *Nannochloropsis limnetica* (*l*) | psaa psab rps14 *psbk psbz rpl11 rpl1 rpl12 psae *psbh psbn *psbt *psbb *rps10 *tufa *rps7 *rps12 *rpl31 *rps9 *rpl13 *rpoa *rps11 *rps13 *rpl36 *rps5 *rpl18 *rpl6 *rps8 *rpl5 *rpl14 *rps17 *rpl29 *rpl16 *rps3 *rpl22 *rps19 *rpl2 *rpl23 *rpl4 *rpl3 ycf34 psbd psbc *psbx *psaf *rpl32 *clpc_2 psac_1 psaj_1 *rpl27 *rpl21 rpl34 rps6 clpc_1 psby_2 *rps2 *rpoc2_1 *rpoc1 *rpob *rps20 rpl33 rps18 ycf3 rbcl *psba *psb1 *psad *ycf36 *rps16 rps4 *psbw psa1 *psbj *psbl *psbf *psbe psal rpl35 rpl20 rpl19_2 clpc_3 psbv_2 |
| *Toxoplasma gondii – Aureoumbra lagunensis* | psal psbk *rpl20 *rpl35 ycf37 *ycf35 rpob rpoc1 rpoc2_1 rps2 rps16 *psad *psbc *psbd *tufa *rps12 *rpoa *rps19 *rpl2 *rpl3 *rps14 rpl27 *psac rpl23 rpl22 rps3 rpl16 rps17 rpl14 rpl5 rps8 rpl6 rps5 rbcl psbb psbt *psbn psbh rps4 *psbv *clpc_1 *psbz psam psbm ycf3 psaf psaj *psb30 psbe rps6 psbf psbl psbj psby_1 *rpl34 rpl11 *rpl4 *rps7 *rpl31 *rps9 *rps11 *rps13 *rpl36 *psa1 psba *rpl21 psaa psab \| psbx rps10 *ycf39 psb1 rpoz *rpl12 *rpl1 rpl19 rpl33 rps18 |
| *Aureococcus anophagefferens – Aureoumbra lagunensis* | psal psbk *rpl20 *rpl35 ycf37 *ycf35 rpob rpoc1 rpoc2_1 rps2 rps16 *psad *psbc *psbd psaj rpl3 rpl23 rpl2 rps19 rpl22 rps3 rpl16 rps17 rpl14 rpl24 rpl5 rps8 rpl6 rps5 rpl36 rps13 rps11 rpoa rpl13 rps9 rpl31 rps12 rps7 tufa rps10 psbb psbt *psbn psbh psbx psbv ycf33 *psbj *psbl *psbf *psbe psaa psab rps14 rbcl ycf39 psb1 *psam psaf *rpl27 *rpl21 *ycf30 *rpl34 *rps6 *rpl1 *rpl11 *rps4 *ycf3 *rps18 *rpl33 psac *clpc_1 psba |
| *Aureococcus anophagefferens* (*l*) | psal psbk *rpl20 *rpl35 ycf37 *ycf35 rpob rpoc1 rpoc2_1 rps2 rps16 *psad *psbc *psbd psaj rpl3 rpl23 rpl2 rps19 rpl22 rps3 rpl16 rps17 rpl14 rpl24 rpl5 rps8 rpl6 rps5 rpl36 rps13 rps11 rpoa rpl13 rps9 rpl31 rps12 rps7 tufa rps10 psbb psbt *psbn psbh psbx psbv ycf33 *psbj *psbl *psbf *psbe psaa psab rps14 rbcl ycf39 psb1 *psam rps4 rpl11 rpl1 rps6 rpl34 ycf30 rpl21 rpl27 *psac rpl33 rps18 ycf3 *clpc_1 psba |
| *Aureoumbra lagunensis* (*l*) | rps16 *psad *psbc *psbd psaf psaj rpl3 rpl23 rpl2 rps19 rpl22 rps3 rpl16 rps17 rpl14 rpl24 rpl5 rps8 rpl6 rps5 rpl36 rps13 rps11 rpoa rpl13 rps9 rpl31 rps12 rps7 tufa rps10 psbb psbt *psbn psbh psbx psbv ycf33 *psbj *psbl *psbf *psbe psaa psab rps14 rbcl ycf39 psb1 *psam *rpl27 *rpl21 *ycf30 *rpl34 *rps6 *rpl1 *rpl11 *rps4 *ycf3 *rps18 *rpl33 rpob rpoc1 rpoc2_1 rps2 psac ycf37 *ycf35 *psbk *psal rpl35 rpl20 *clpc_1 psba |
| *Toxoplasma gondii – Choreocolax polysiphoniae* | *tufa *rps12 *rpoa *rps19 *rpl2 *rpl3 *rps14 rpl20 rpoc1 rpl27 *psac rpl23 rpl22 rps3 rpl16 rps17 rpl14 rpl5 rps8 rpl6 rps5 rbcl psbb psbt *psbn psbh rps4 *psbv *clpc_1 *psbz psbk psam psbm ycf3 psbd psbc *rps2 *rpoc2_1 *rpob psaf psaj psal *psb30 psbe rps6 psbf psbl psbj psby_1 *rps16 *rpl34 rpl11 *rpl4 *rps7 *rpl31 *rps9 *rps11 *rps13 *rpl36 *psad *psa1 psba *rpl21 psaa psab \| psbx *ycf35 rps10 *ycf39 psb1 rpoz *rpl12 *rpl1 rpl19 rpl33 rps18 |
| *Choreocolax polysiphoniae* (*l*) | *tufa *rps12 *rpoa *rps19 *rpl2 *rpl3 *rps14 rpl20 rpoc1 rpl27 *rpl12 *rpl1 *clpc_1 rpl19 *rps10 *rps7 *rpl31 *rps9 *rpl13 *rps11 *rps13 *rpl36 *rps5 *rpl18 *rpl6 *rps8 *rpl5 *rpl14 *rpl29 *rpl16 *rps3 *rpl22 *rpl23 *rpl4 rpob rpoc2_1 rps2 rps4 *rps6 *rpl21 rps16 *rpl11 |
| *Toxoplasma gondii – Pavlova lutheri* | *psac rpl3 rpl23 rpl2 rps19 rpl22 rps3 rpl16 rps17 rpl14 rpl5 rps8 rpl6 rps5 rbcl psbb psbt *psbn psbh rps4 *psbv *clpc_1 *psbz psbk psam psbm ycf3 psbd psbc *rps2 *rpoc2_1 *rpoc1 *rpob psaf psaj psal *psb30 psbe rps6 psbf psbl psbj psby_1 *rps16 *rpl34 *rps14 rpl11 *rpl4 *rps7 *rps12 *rpl31 *rps9 *rpoa *rps11 *rps13 *rpl36 *psad *psa1 *rpl20 psba *rpl21 psaa psab *rpl27 *tufa \| psbx *ycf35 rps10 *ycf39 psb1 rpoz *rpl19 rpl33 rps18 |
| *Pavlova lutheri* (*l*) | *psac rpl3 rpl23 rpl2 rps19 rpl22 rps3 rpl16 rps17 rpl14 rpl5 rps8 rpl6 rps5 rbcl psbb psbt *psbn psbh rps4 *psbv *clpc_1 *psbz psbk psam rpoz *rpl19 *rps10 *tufa *rps7 *rps12 *rpl31 *rps9 *rpoa *rps11 *rps13 *rpl36 *rps6 psaf psaj *rps16 *psad *rpl27 *rpl21 psal *psbc *psbd *rps2 *rpoc2_1 *rpoc1 *rpob *psby_2 rpl20 ycf3 psa1 *psbj *psbl *psbf *psbe *psb1 *psbx rpl33 rps18 ycf39 rps14 psba psaa psab |
| *Toxoplasma gondii – Lepidodinium chlorophorum* | psbm ycf3 psbd psbc *rps2 *rpoc2_1 *rpoc1 *rpob psaf psaj psal *psb30 psbe rps6 psbf psbl psbj psby_1 *rps16 *rpl34 *rps14 rpl11 *rpl4 *rps4 *rps7 *rps12 *rpl31 *rps9 *rpoa *rps11 *rps13 *rpl36 *rps5 *rpl6 *rps8 *rpl5 *rpl14 *rps17 *rpl16 *rps3 *rpl22 *rps19 *rpl2 *rpl23 *rpl3 *psbv *psad psam *psa1 psac *psbh psbn *psbt *psbb *psbz psbk *rpl20 psba *rpl21 psaa psab rbcl *rpl27 *clpc_1 *tufa \| psbx *ycf35 rps10 *ycf39 psb1 *rpl19 rpl33 rps18 |
| *Lepidodinium chlorophorum* (*l*) | psaa psab rpl23 rpl2 rps19 rps3 rpl16 rpl14 rpl5 rpl36 rps11 rpoa rps9 psbd psbc rpob *psba *rps14 *psac *rpoc2_1 *psb1 *psbz *psbj *psbl *psbf *psbe *ycf3 *psbm tufa rpl19 rps2 rbcl psbb psbt psbn psbh psbk *rpl20 rps18 psaj rps12 rps7 rps4 psa1 |
| *Toxoplasma gondii – Phaeocystis globosa* | psbm rpl11 *rpl4 *rps4 \| psby_1 *rps16 *rpl34 *rps14 ycf3 psbd psbc *rps2 *rpoc2_1 *rpoc1 *rpob psaf psaj psal *psb30 psbe rps6 psbf psbl psbj \| psbx *ycf35 rps10 *ycf39 psb1 rpl21 rpl20 *psba rpl27 *rbcl *psab *psaa *rpl19 rpl33 rps18 \| rpl31 rps12 rps7 tufa clpc_1 *psbk psbz psbb psbt *psbn psbh *psac psa1 *psam psad psbv rpl3 rpl23 rpl2 rps19 rpl22 rps3 rpl16 rps17 rpl14 rpl5 rps8 rpl6 rps5 rpl36 rps13 rps11 rpoa rps9 |
| *Phaeocystis antarctica – Phaeocystis globosa* | psby_1 *rps16 *rpl34 *rps14 ycf3 psbd psbc *rps2 *rps4 *rpoc2_1 *rpoc1 *rpob *psaj *psaf rbcl *rpl27 *rpl20 *rpl21 ycf39 psb1 *rpl19 rpl33 rps18 psbx *ycf35 rps6 *psba *psam psad psbv rpl3 rpl23 rpl2 rps19 rpl22 rps3 rpl16 rps17 rpl14 rpl5 rps8 rpl6 rps5 rpl36 rps13 rps11 rpoa rps9 rpl31 rps12 rps7 tufa rps10 psaa psab psal *psb30 clpc_1 *psbk psbz psbb psbt *psbn psbh psac psa1 psbe psbf psbl psbj |
| *Phaeocystis antarctica* (*l*) | ycf39 psb1 *rpl19 rpl33 rps18 psbx *ycf35 rps6 *psba *psbv *psad psam rpl3 rpl23 rpl2 rps19 rpl22 rps3 rpl16 rps17 rpl14 rpl5 rps8 rpl6 rps5 rpl36 rps13 rps11 rpoa rps9 rpl31 rps12 rps7 tufa rps10 psaa psab psal *psb30 clpc_1 *psbk psbz psbb psbt *psbn psbh psac psa1 psbe psbf psbl psbj psby_1 *rps16 *rpl34 *rps14 ycf3 psbd psbc *rps2 *rps4 *rpoc2_1 *rpoc1 *rpob *psaj *psaf rbcl *rpl27 *rpl20 *rpl21 |
| *Phaeocystis globosa* (*l*) | ycf39 psb1 *rpl19 rpl33 rps18 psbx *ycf35 rps6 *psba *psam psad psbv rpl3 rpl23 rpl2 rps19 rpl22 rps3 rpl16 rps17 rpl14 rpl5 rps8 rpl6 rps5 rpl36 rps13 rps11 rpoa rps9 rpl31 rps12 rps7 tufa rps10 psaa psab psal *psb30 clpc_1 *psbk psbz psbb psbt *psbn psbh psac psa1 psbe psbf psbl psbj psby_1 *rps16 *rpl34 *rps14 ycf3 psbd psbc *rps2 *rps4 *rpoc2_1 *rpoc1 *rpob *psaj *psaf rbcl *rpl27 *rpl20 *rpl21 |
| *Toxoplasma gondii – Emiliania huxleyi* | psbm rpl11 *rpl4 *rps4 \| ycf39 *rps10 ycf35 *rps18 *rpl33 rpl19 psb1 rpl21 rpl20 *psba *rbcl *psab *psaa \| rpl31 rps12 rps7 tufa clpc_1 *psbk psbz psbb psbt *psbn psbh *psac psaf psaj psal *rpl27 rpob rpoc1 rpoc2_1 rps2 *psbc *psbd *ycf3 rps14 rpl34 rps16 *psbj *psbl *psbf *psbe rps6 psa1 *psam psad psbv rpl3 rpl23 rpl2 rps19 rpl22 rps3 rpl16 rps17 rpl14 rpl5 rps8 rpl6 rps5 rpl36 rps13 rps11 rpoa rps9 |
| *Emiliania huxleyi* (*l*) | psaa psab rbcl psba *rpl20 *rpl21 *psb1 *rpl19 rpl33 rps18 *ycf35 clpc_1 *psbk psbz psbb psbt *psbn psbh *psac psaf psaj psal *rpl27 rpob rpoc1 rpoc2_1 rps4 rps2 *psbc *psbd *ycf3 rps14 rpl34 rps16 *psbj *psbl *psbf *psbe rps6 psa1 *psam psad psbv rpl3 rpl23 rpl2 rps19 rpl22 rps3 rpl16 rps17 rpl14 rpl5 rps8 rpl6 rps5 rpl36 rps13 rps11 rpoa rps9 rpl31 rps12 rps7 tufa rps10 *ycf39 |
| *Toxoplasma gondii – Theileria parva* | rpob rpoc1 rpoc2_1 rpoc2_2 rps2 rps4 rpl4 *clpc_1 *tufa *rps7 *rps12 *rps11 *rpl36 *rps5 *rpl6 *rps8 *rpl14 *rps17 *rpl16 *rps3 *rps19 *rpl2 *rpl23 |
| *Theileria parva* (*l*) | rps4 rpl4 rpl2 rps19 rps3 rpl16 rpl14 rps8 rpl6 rps5 rpl36 rps11 rps12 rps7 tufa clpc_1 clpc_2 rpob rpoc1 rpoc2_1 rpoc2_2 rps2 |
| *Toxoplasma gondii – Plasmodium chabaudi* | rpoc2_2 rps2 *clpc_1 *tufa *rps7 *rps12 *rps11 *rpl36 *rps5 *rpl6 *rps8 *rpl14 *rps17 *rpl16 *rps3 *rps19 *rpl2 *rpl23 *rpl4 *rps4 rpob rpoc1 rpoc2_1 |
| *Leucocytozoon caulleryi – Plasmodium chabaudi* | rps4 rpl4 rpl23 rpl2 rps19 rps3 rpl16 rps17 rpl14 rps8 rpl6 rps5 rpl36 rps11 rps12 rps7 tufa clpc_1 *rps2 *rpoc2_2 *rpoc2_1 *rpoc1 *rpob |
| *Leucocytozoon caulleryi* (*l*) | rps4 rpl4 rpl23 rpl2 rps19 rps3 rpl16 rps17 rpl14 rps8 rpl6 rps5 rpl36 rps11 rps12 rps7 tufa clpc_1 *rps2 *rpoc2_2 *rpoc2_1 *rpoc1 *rpob |
| *Plasmodium chabaudi* (*l*) | rps4 rpl4 rpl23 rpl2 rps19 rps3 rpl16 rps17 rpl14 rps8 rpl6 rps5 rpl36 rps11 rps12 rps7 tufa clpc_1 *rps2 *rpoc2_2 *rpoc2_1 *rpoc1 *rpob |
| *Toxoplasma gondii – Eimeria tenella* | rps2 *clpc_1 *rpl11 *tufa *rps7 *rps12 *rps11 *rpl36 *rps5 *rpl6 *rps8 *rpl14 *rps17 *rpl16 *rps3 *rps19 *rpl2 *rpl4 *rps4 rpob rpoc1 rpoc2_1 |
| *Toxoplasma gondii* (*l*) | rps4 rpl4 rpl2 rps19 rps3 rpl16 rps17 rpl14 rpl6 rps5 rpl36 rps11 rps12 rps7 tufa *rpl11 clpc_1 *rps2 *rpoc1 *rpob |
| *Eimeria tenella* (*l*) | rps4 rpl4 rpl2 rps19 rps3 rpl16 rps17 rpl14 rps8 rpl6 rps5 rpl36 rps11 rps12 rps7 tufa rpl11 clpc_1 *rps2 *rpoc2_1 *rpoc1 *rpob |
